# Supplementary material for: The lipid transfer protein STARD7 controls intestinal tumor development in a context-dependent manner
Source: EMBO Mol Med. 2026 Mar 30;18(5):1771–811. doi: 10.1038/s44321-026-00409-5 (PMC13179355; doi:10.1038/s44321-026-00409-5)
Supplement: Supplementary file 1 — Table EV1 [file 44321_2026_409_MOESM1_ESM.docx]

| **ShRNAs** | **Company** | **TRC number** | **Clone ID** | **Sequence** |
| --- | --- | --- | --- | --- |
| ShRNAbeta-Catenin#1 (human) | MERCK | TRCN0000314920 | NM_001904 | GCTTGGAATGAGACTGCTGAT |
| ShRNAbeta-Catenin #2 (human) | MERCK | TRCN0000314921 | NM_001904 | TCTAACCTCACTTGCAATAAT |
| shRNA Stard7 #2 (human) | MERCK | TRCN0000280817 | NM_020151 | GCTGGACACAGAGTATAGAAA |
| shRNA Stard7 #4 (human) | MERCK | TRCN0000155648 | NM_020151 | GCTGGACACAGAGTATAGAAA |
| shRNA Stard7 #2 (mouse) | MERCK | TRCN0000105121 | NM_139308 | CGGTTGGAAGAAATGTCAAAT |
| shRNA Stard7 #3 (mouse) | MERCK | TRCN0000105122 | NM_139308 | CTGAGGTTCTTCATTGGGTAA |
|  |  |  |  |  |

**Table EV1: List of ShRNAs used in this study.**
