# Supplementary material for: The lipid transfer protein STARD7 controls intestinal tumor development in a context-dependent manner
Source: EMBO Mol Med. 2026 Mar 30;18(5):1771–811. doi: 10.1038/s44321-026-00409-5 (PMC13179355; doi:10.1038/s44321-026-00409-5)
Supplement: Supplementary file 2 — Table EV2 [file 44321_2026_409_MOESM2_ESM.docx]

| **Name** | **Sequence** |
| --- | --- |
| Gapdh For | TGATGACATCAAGAAGGTGGTGAAG |
| Gapdh Rev | TCC TTG GAG GCC ATG TAG GCC AT |
| Lgr-5 For | GAGCCTGATACCATCTGCAA |
| Lgr-5 Rev | GCCGTCGTCTTTATTCCATT |
| Olfm4 For | AATTGACCACACCTCCAACA |
| Olfm4 Rev | CCACAGACCCAGTGAAATTG |
| Bmi1 For | GAAGACCGAGGAGAAGTTGC |
| Bmi1 Rev | ACTTTCCGATCCAATCTGCT |
| CD44 For | CTCCTGGCACTGGCTCTGA |
| CD44 Rev | CTGCCCACACCTTCTCCTACTATT |
| c-Myc For | ATCTCTGGACATCCGAAAGC |
| c-Myc Rev | TTATGCTGCTGTTGGTGGAT |
| EphB2 For | CAGTTCGCCTCTGTGAACAT |
| EphB2 Rev | CAGCTCGTAGTCCAGGATCA |
| Sox9 For | ACTCCCCACATTCCTCCTC |
| Sox9 Rev | TCGCTTCAGATCAACTTTGC |
| Stard7 For | CCAATTACAGGCACCCACCT |
| Stard7 Rev | TTGATCACAAGGGCATCCCA |
| IL-17alpha For | GCCCTCAGACTACCTCAACC |
| IL-17alpha Rev | CAGCTTTCCCTCCGCATTGA |
| TNF For | CCCTCACACTCAGATCATCTTCT |
| TNF Rev | GCTACGACGTGGGCTACAG |
| IL-6 For | TAGTCCTTCCTACCCCAATTTCC |
| IL-6 Rev | TTGGTCCTTAGCCACTCCTTC |
| IL-1beta For | GCAACTGTTCCTGAACTCAACT |
| IL-1beta Rev | ATCTTTTGGGGTCCGTCAACT |
| PSPH For | CCACATCTGACTCCTGGCATAAG |
| PSPH Rev | AGCTTTGCAGCAACGTGCTCCA |
| ASNS For | GACTCTAAGGTGGGAAGCGG |
| ASNS Rev | CAGGCACTCTGAGCACTAGC |
| PSAT1 For | CATTGGCAACGCCAAAGGAGAC |
| PSAT1 Rev | GTGACAGCGTTATACAGAGAGGC |
| Phgdh For | CCTCCTTTGGTGTTCAGCAGCT |
| Phgdh Rev | CGCACACCTTTCTTGCACTGAG |
| Mthfd2 For | TTCCTTGTTGTCTGCGTTGG |
| Mthfd2 Rev | TGACAACGGCTTCATTTCGC |
| hACTB For | AGAGCTACGAGCTGCCTGAC |
| hACTB Rev | AGCACTGTGTTGGCGTACAG |
| hSTARD7 For | ATTCAGAGGGCAAAGAGCAA |
| hSTARD7 Rev | AAGGTGGGTGCCTGTAATTG |
| hMyc For | CCGCTTCTCTGAAAGGCTCT |
| hMyc Rev | CTAACGTTGAGGGGCATCGT |
| hSox9 For | GGAGACTTCTGAACGAGAGCG |
| hSox9 Rev | CCGTTCTTCACCGACTTCCTC |

**Table EV2: List of primers used in this study.**
