# Supplementary material for: The lipid transfer protein STARD7 controls intestinal tumor development in a context-dependent manner
Source: EMBO Mol Med. 2026 Mar 30;18(5):1771–811. doi: 10.1038/s44321-026-00409-5 (PMC13179355; doi:10.1038/s44321-026-00409-5)
Supplement: Supplementary file 3 — Table EV3 [file 44321_2026_409_MOESM3_ESM.pdf]

Metabolites	Mice numbers and corresponding genotypes					
	1794	9806	9808	1782	9846	1904
	WT APCmin	WT APCmin	WT APCmin	KO APCmin	KO APCmin	KO APCmin
**3-hydroxybutyric acid**	1,19E+08	9,94E+07	1,03E+08	9,63E+07	9,93E+07	1,19E+08
**5-Methylcytosine**	2,50E+05	4,23E+05	2,39E+05	1,47E+05	7,30E+05	1,37E+06
**5-oxo-L-Proline**	2,45E+07	1,32E+07	1,05E+07	5,17E+07	2,87E+07	6,44E+07
**Acetyl CoA**	2,35E+05	7,34E+04	1,44E+05	1,57E+06	5,29E+04	9,22E+05
**acetyl-aspartate (N)**	2,38E+06	2,84E+06	3,93E+06	7,14E+06	4,32E+06	7,23E+06
**acetyl-carnitine**	6,80E+08	5,30E+08	7,68E+08	1,80E+09	6,29E+08	1,13E+09
**acetyl-glutamine**	6,85E+05	1,25E+06	1,22E+06	1,16E+06	1,08E+06	1,32E+06
**acetyl-lysine**	1,60E+06	4,21E+06	6,74E+06	5,39E+06	4,55E+06	4,81E+06
**Adenine**	4,19E+07	4,42E+07	3,26E+07	2,68E+07	5,37E+07	2,96E+07
**Adenosine**	1,53E+08	7,46E+07	5,86E+07	1,60E+08	1,45E+08	1,25E+08
**ADP**	3,89E+07	4,45E+07	4,20E+07	5,06E+07	3,60E+07	2,73E+07
**aKG**	4,39E+06	1,39E+07	1,18E+07	3,81E+06	2,19E+07	3,32E+06
**Aminoadipate**	2,26E+07	1,52E+07	1,33E+07	1,83E+07	2,77E+07	1,65E+07
**AMP**	2,95E+08	3,19E+08	4,56E+08	5,50E+08	3,35E+08	4,05E+08
**Arachidonic acid**	2,13E+09	3,82E+09	4,49E+09	7,22E+08	3,61E+09	1,38E+10
**Arginine**	1,48E+08	1,04E+08	1,23E+08	1,94E+08	1,26E+08	1,93E+08
**Argininosuccinate**	3,97E+04	1,99E+05	3,27E+05	4,44E+05	2,69E+05	3,09E+05
**Asparagine**	8,63E+06	8,39E+06	1,77E+07	3,55E+07	1,99E+07	3,28E+07
**Aspartate**	8,05E+07	9,20E+07	1,47E+08	2,36E+08	1,20E+08	1,88E+08
**ATP**	4,28E+06	5,20E+06	3,10E+06	7,18E+06	2,38E+06	1,63E+06
**Behenic acid**	5,74E+07	9,62E+07	8,28E+07	1,15E+07	7,87E+07	9,99E+07
**beta-Alanine**	3,25E+06	6,80E+06	1,27E+07	1,80E+07	9,82E+06	9,87E+06
**betaine**	5,34E+09	3,95E+09	5,45E+09	8,79E+09	5,15E+09	8,71E+09
**Butyric acid**	1,73E+07	1,99E+07	2,38E+07	1,98E+07	1,98E+07	1,95E+07
**Butyryl-carnitine**	1,87E+07	6,44E+07	7,68E+07	1,17E+08	7,93E+07	6,39E+07
**carnitine**	1,21E+09	8,64E+08	1,42E+09	3,95E+09	1,31E+09	2,91E+09
**CDP**	1,73E+05	3,21E+05	2,44E+05	3,95E+05	3,39E+05	2,01E+04
**CDP-ethanolamine**	2,50E+06	2,75E+06	4,80E+06	8,90E+06	3,29E+06	5,09E+06
**cis-aconitate**	1,37E+07	1,84E+07	2,56E+07	3,69E+07	1,51E+07	1,25E+07
**Citrate**	2,23E+08	2,35E+08	2,84E+08	7,12E+08	1,54E+08	1,30E+08
**Citrulline**	2,81E+07	7,33E+07	8,55E+07	4,70E+07	5,93E+07	7,34E+07
**CMP**	4,06E+06	5,10E+06	8,81E+06	1,17E+07	6,11E+06	8,45E+06
**Coanzyme A**	2,81E+06	1,01E+06	3,36E+06	4,03E+06	9,74E+05	2,07E+06
**Coenzyme Q9**	0,00E+00	0,00E+00	0,00E+00	0,00E+00	0,00E+00	1,05E+06
**Creatine**	7,18E+07	7,90E+07	1,19E+08	2,56E+08	9,01E+07	1,32E+08
**Creatinine**	5,12E+07	2,33E+07	4,08E+07	4,61E+07	2,34E+07	3,45E+07
**Cystathionine**	1,12E+06	2,83E+06	4,37E+06	3,40E+06	1,85E+06	3,15E+06
**Cysteinesulfinic acid**	2,33E+06	2,36E+06	3,38E+06	2,63E+06	4,17E+06	5,90E+06
**Cystine**	9,10E+05	1,49E+06	2,35E+06	4,76E+06	3,16E+06	3,32E+06
**Cytidine**	1,04E+07	7,89E+06	1,13E+07	2,75E+07	1,10E+07	3,36E+07

**Cytidine diphosphate-choline**	5,66E+05	7,69E+05	1,21E+06	2,11E+06	8,71E+05	3,08E+06
**Decanoic acid**	8,97E+06	4,28E+07	3,37E+07	2,98E+07	9,11E+06	2,07E+07
**Dephospho-CoA**	4,73E+05	2,90E+05	4,71E+05	2,71E+06	5,10E+05	1,96E+06
**dihydroxyacetone phosphate (DHAP)**	2,32E+07	2,52E+07	2,45E+07	7,32E+07	2,52E+07	1,84E+07
**Docosahexaenoic acid**	4,95E+08	6,99E+08	7,18E+08	2,64E+08	6,15E+08	2,68E+09
**Dodecanoic acid/Lauric acid**	1,50E+07	2,96E+07	2,88E+07	2,45E+07	4,06E+06	1,45E+07
**Eicosapentaenoic acid**	6,66E+07	1,57E+08	1,95E+08	2,09E+07	1,17E+08	5,55E+08
**FAD**	1,36E+07	1,19E+07	1,26E+07	1,57E+07	1,05E+07	1,17E+07
**Flavin mononucleotide**	1,41E+06	1,06E+06	8,01E+05	2,42E+05	4,51E+05	4,07E+05
**Fructose**	1,47E+07	9,59E+06	9,73E+06	1,32E+07	8,84E+06	8,99E+06
**fructose 1,6-diphosphate**	3,60E+06	3,21E+06	2,09E+06	9,47E+06	2,91E+06	3,50E+06
**Fumarate**	8,24E+06	1,30E+07	2,06E+07	3,43E+07	1,34E+07	1,89E+07
**G6P**	1,97E+07	1,14E+07	1,77E+07	3,66E+07	1,58E+07	2,87E+07
**GDP**	4,53E+06	3,94E+06	2,56E+06	2,50E+06	3,02E+06	1,44E+06
**GLN**	3,09E+08	4,02E+08	4,09E+08	7,62E+08	4,33E+08	5,89E+08
**GLU**	3,61E+08	6,12E+08	6,56E+08	6,91E+08	5,89E+08	7,65E+08
**Glucose**	1,01E+08	7,41E+07	6,10E+07	5,07E+07	5,48E+07	8,43E+07
**Glyceraldehyde 3-phosphate**	8,33E+01	5,97E+04	1,18E+05	3,65E+05	2,99E+05	2,04E+05
**Glycerol**	8,15E+06	7,41E+06	7,17E+06	5,44E+06	8,10E+06	8,59E+06
**Glycerol 3-phosphate**	1,28E+07	2,75E+07	1,31E+07	2,33E+07	2,85E+07	4,53E+07
**Glycine**	2,49E+07	1,33E+07	2,01E+07	2,58E+07	1,84E+07	2,73E+07
**GMP**	2,36E+07	2,36E+07	3,52E+07	4,61E+07	2,74E+07	4,21E+07
**GSH**	5,22E+08	7,49E+08	5,33E+08	7,32E+08	5,60E+08	7,78E+08
**GSSG**	8,83E+06	1,15E+07	4,25E+07	2,90E+07	1,58E+07	2,52E+07
**guanine**	4,59E+06	3,72E+06	2,64E+06	4,95E+06	4,43E+06	4,36E+06
**guanosine**	1,78E+06	1,41E+06	1,09E+06	4,46E+06	1,97E+06	3,51E+06
**Hexanoic acid**	4,14E+07	3,68E+07	2,24E+07	2,26E+07	2,68E+07	2,77E+07
**Hexanoyl-carnitine**	6,62E+05	3,11E+06	3,35E+06	3,36E+06	2,73E+06	3,25E+06
**Histidine**	4,71E+07	4,23E+07	8,53E+07	1,24E+08	6,56E+07	1,18E+08
**Hydroxy-L-proline**	1,00E+07	8,74E+06	7,44E+06	2,04E+07	3,67E+06	7,60E+06
**hypoxanthine**	5,22E+08	2,83E+08	2,95E+08	7,93E+08	3,09E+08	8,92E+08
**IMP**	1,02E+07	1,65E+07	3,04E+07	4,53E+07	2,14E+07	4,97E+07
**Inosine**	3,38E+07	5,39E+06	4,23E+06	2,91E+07	6,17E+06	4,14E+07
**IsoLeucine**	1,93E+08	1,47E+08	2,54E+08	2,97E+08	1,48E+08	3,19E+08
**Itaconic acid**	3,85E+06	5,15E+06	6,77E+06	9,78E+06	4,07E+06	3,25E+06
**Kynurenic acid**	3,03E+06	2,18E+05	1,79E+05	3,86E+04	2,80E+05	0,00E+00
**L-(?)-Fucose**	2,48E+06	3,15E+06	4,06E+06	3,04E+06	2,70E+06	3,17E+06
**Lactate**	1,94E+08	2,74E+08	4,18E+08	3,85E+08	3,11E+08	7,43E+08
**L-Alanine**	1,72E+08	1,94E+08	2,61E+08	3,38E+08	2,06E+08	2,25E+08
**Leucine**	9,32E+07	8,01E+07	1,37E+08	1,84E+08	7,62E+07	1,69E+08
**Linolenic acid**	1,86E+08	1,89E+08	1,83E+08	4,95E+07	1,63E+08	5,90E+08
**L-Kynurenine**	1,07E+07	4,81E+07	9,02E+07	3,81E+07	5,28E+07	2,34E+07
**L-Sarcosine**	6,37E+06	7,48E+06	1,07E+07	1,41E+07	8,04E+06	3,48E+07

**Lysine**	2,81E+07	1,85E+07	2,43E+07	4,21E+07	2,37E+07	3,93E+07
**Malate**	8,68E+07	1,42E+08	2,37E+08	4,19E+08	1,45E+08	2,25E+08
**Malonic acid**	3,87E+07	2,63E+06	2,81E+06	3,14E+06	1,09E+06	2,60E+06
**Methionine**	1,15E+08	8,30E+07	1,22E+08	1,74E+08	9,64E+07	2,03E+08
**methyl-lysine (N)**	2,61E+06	2,90E+06	4,21E+06	1,02E+07	4,49E+06	7,12E+06
**Methylmalonic acid**	1,03E+07	8,73E+06	9,68E+06	2,25E+07	9,98E+06	2,62E+07
**Myristic acid**	1,42E+08	1,83E+08	2,14E+08	6,53E+07	1,83E+08	9,30E+08
**Myristoyl-carnitine**	7,23E+06	8,18E+06	6,38E+06	8,05E+06	7,89E+06	4,16E+07
**NAD+**	1,19E+06	4,14E+06	2,90E+06	4,12E+06	2,82E+06	2,19E+06
**NADH**	0,00E+00	1,35E+05	1,14E+05	3,97E+05	1,11E+05	0,00E+00
**NADP+**	2,11E+05	4,37E+05	2,67E+05	1,95E+05	1,92E+05	1,39E+05
**N-carbamoyl-L-aspartic acid**	4,18E+04	6,41E+04	1,87E+05	5,04E+05	6,74E+04	1,33E+05
**niacin / nicotinate**	4,11E+05	2,32E+06	1,94E+06	7,91E+05	2,49E+06	1,45E+06
**nicotinamide**	5,63E+08	5,37E+08	6,70E+08	9,60E+08	5,40E+08	6,49E+08
**nicotinamide N-oxide**	3,38E+07	1,86E+07	7,05E+06	1,31E+08	2,89E+07	1,12E+08
**Octadecanamide**	3,64E+05	2,19E+05	1,72E+05	4,46E+05	3,29E+05	4,82E+05
**Octanoic acid**	1,69E+07	1,76E+07	1,83E+07	1,17E+07	1,48E+07	1,35E+07
**Oleamide**	2,28E+06	3,08E+06	2,86E+06	2,97E+06	6,48E+06	7,98E+06
**Oleic acid**	6,67E+09	7,17E+09	7,80E+09	1,39E+09	6,29E+09	1,83E+10
**O-Phosphoethanolamine**	9,52E+06	9,37E+06	1,65E+07	3,60E+07	1,91E+07	2,69E+07
**Ornithine**	1,29E+07	9,58E+06	1,07E+07	2,07E+07	1,20E+07	3,22E+07
**Orotic acid**	1,74E+06	1,37E+06	3,01E+06	7,79E+06	1,90E+06	3,41E+06
**oxypurinol**	4,27E+07	4,44E+07	5,29E+07	1,29E+08	5,11E+07	1,32E+08
**Palmitic acid**	7,50E+09	8,06E+09	8,29E+09	2,10E+09	6,79E+09	1,76E+10
**Palmitoleic acid**	7,16E+08	1,03E+09	9,74E+08	2,06E+08	6,58E+08	3,73E+09
**Palmitoyl-carnitine**	2,46E+07	2,42E+07	2,57E+07	9,83E+07	3,64E+07	1,01E+08
**Pantetheine 4'-phosphate**	1,45E+06	3,36E+05	4,47E+05	3,35E+06	3,17E+05	1,17E+06
**Pantothenate**	6,27E+05	8,70E+05	1,09E+06	3,25E+06	1,38E+06	2,14E+06
**PEP**	3,08E+07	2,73E+07	2,22E+07	4,68E+07	3,17E+07	3,73E+07
**Phenylalanine**	1,02E+08	7,59E+07	1,18E+08	1,76E+08	1,00E+08	1,97E+08
**Phosphocholine**	4,83E+08	3,40E+08	7,19E+08	1,41E+09	4,92E+08	1,07E+09
**phosphocreatine**	4,49E+06	3,81E+06	4,32E+06	1,71E+07	4,23E+06	3,29E+06
**Phytanic acid**	7,36E+07	9,28E+07	8,81E+07	1,93E+07	8,67E+07	1,24E+08
**Proline**	5,18E+08	6,57E+08	1,02E+09	1,54E+09	8,62E+08	1,77E+09
**Propionyl-carnitine**	6,90E+07	1,69E+08	1,93E+08	5,51E+08	2,59E+08	2,85E+08
**Pyruvate**	2,62E+07	2,75E+07	4,17E+07	1,71E+08	5,78E+07	9,25E+07
**Quinolinic acid**	3,07E+05	5,32E+04	1,82E+05	3,98E+05	1,98E+05	2,80E+05
**riboflavin**	1,76E+05	2,37E+05	5,09E+05	5,47E+05	2,94E+05	7,78E+05
**Ribose phosphate**	2,86E+07	1,74E+07	2,51E+07	5,27E+07	2,41E+07	2,88E+07
**S-Adenosyl-L-Homocysteine**	3,35E+06	2,26E+06	3,06E+06	4,22E+06	1,73E+06	2,62E+06
**S-Adenosyl-L-methionine**	2,14E+06	2,52E+06	3,12E+06	4,08E+06	2,34E+06	3,81E+06
**Sedoheptulose-7-phosphate (D)**	2,49E+06	1,62E+06	2,60E+06	4,04E+06	2,17E+06	2,57E+06
**Serine**	2,43E+07	2,29E+07	2,91E+07	5,51E+07	2,97E+07	4,39E+07

**Serotonin**	6,57E+07	7,05E+07	6,91E+07	1,76E+08	3,82E+07	2,87E+07
**Stearic acid**	3,30E+09	4,00E+09	4,24E+09	1,10E+09	3,93E+09	7,63E+09
**Succinic acid**	9,19E+06	7,81E+06	9,09E+06	2,20E+07	9,89E+06	2,39E+07
**Taurine**	1,01E+09	1,48E+09	2,06E+09	1,59E+09	1,23E+09	1,70E+09
**Threonine**	8,09E+07	1,04E+08	1,59E+08	1,94E+08	9,17E+07	1,90E+08
**Thymidine**	5,90E+04	9,07E+05	1,59E+05	4,82E+04	5,79E+04	9,23E+04
**Tryptophan**	1,60E+07	7,45E+06	8,54E+06	2,93E+07	6,62E+06	3,37E+07
**Tyrosine**	9,38E+07	8,59E+07	1,46E+08	1,77E+08	1,23E+08	1,90E+08
**UDP**	4,22E+06	7,00E+06	5,14E+06	5,73E+06	6,03E+06	2,01E+06
**UDP-GlcNAc**	1,34E+07	2,97E+07	2,96E+07	3,89E+07	2,36E+07	2,20E+07
**UMP**	7,58E+07	8,63E+07	7,30E+07	9,36E+07	6,80E+07	5,88E+07
**urate**	4,82E+07	7,27E+07	4,50E+04	1,43E+08	7,70E+07	1,14E+08
**UTP**	1,58E+05	4,73E+05	1,16E+05	4,93E+05	9,05E+04	0,00E+00
**Valine**	6,55E+07	7,93E+07	1,33E+08	1,40E+08	6,11E+07	1,54E+08
**Xanthine**	4,83E+07	4,97E+07	5,85E+07	1,48E+08	5,70E+07	1,50E+08
**Xanthosine**	3,98E+05	4,73E+05	6,48E+05	1,34E+06	4,98E+05	1,24E+06
**XMP**	3,44E+05	7,22E+05	8,27E+05	8,24E+05	7,21E+05	5,86E+05

Table EV3: Relative metabolite levels in WT versus KO Stard7 mice (Apc+/Min background)
