# Supplementary material for: The lipid transfer protein STARD7 controls intestinal tumor development in a context-dependent manner
Source: EMBO Mol Med. 2026 Mar 30;18(5):1771–811. doi: 10.1038/s44321-026-00409-5 (PMC13179355; doi:10.1038/s44321-026-00409-5)
Supplement: Supplementary file 4 — Appendix [file 44321_2026_409_MOESM4_ESM.pdf]

Densitometry analyses of western blots

Figure number	Page
1A	2
1G	2
1l	3
1J	4
3B	5
3C	5
3D	6
5E	7
6C	8
6E	10
7B	11
7C	12
EV1E	13
EV2B	13
EV3B	14
EV4D	15
EV4E	15
EV4F	16
EV4G	17
EV4H	17
EV5A	18
EV8A	19
EV9	20

Figure 1A

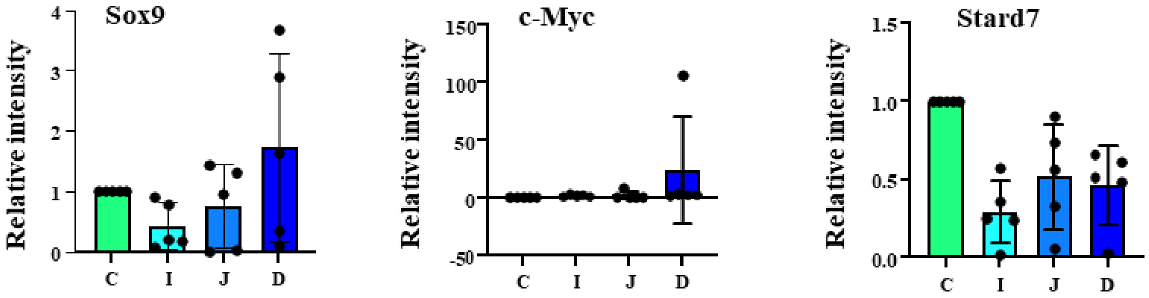

Figure 1G

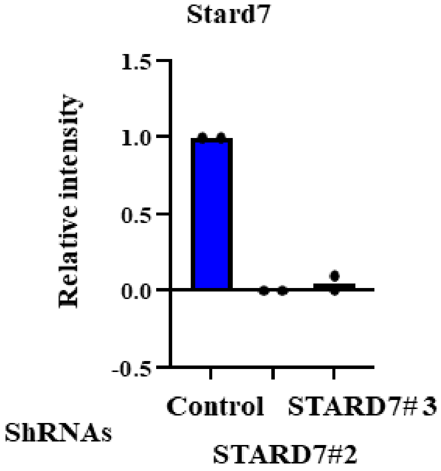

Figure 1I

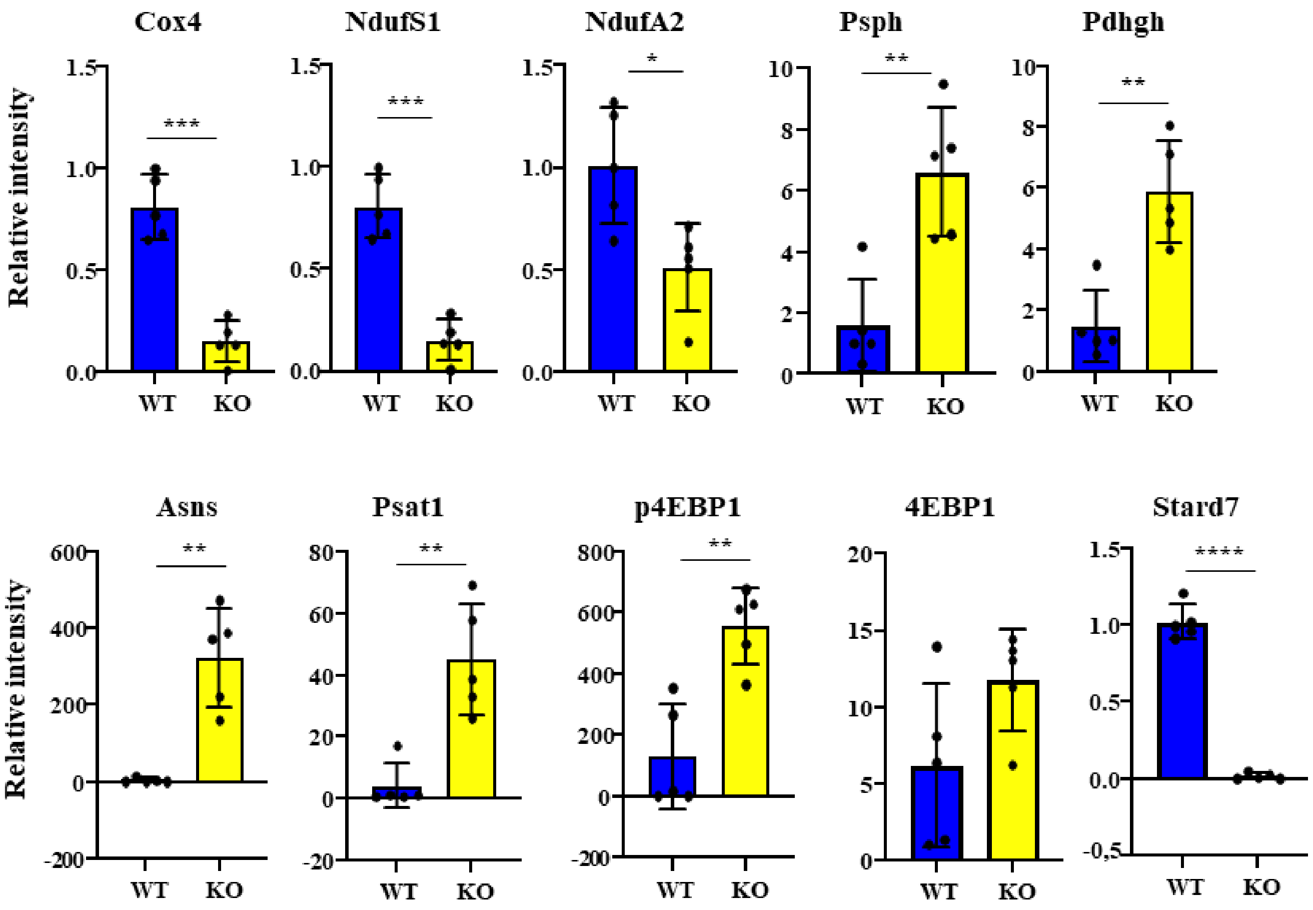
Cox4
NdufS1
NdufA2
Psph
Pdhgh
Relative intensity
WT
KO
***
***
*
**
**
Asns
Psat1
p4EBP1
4EBP1
Stard7
**
**
**
****

Figure 1J

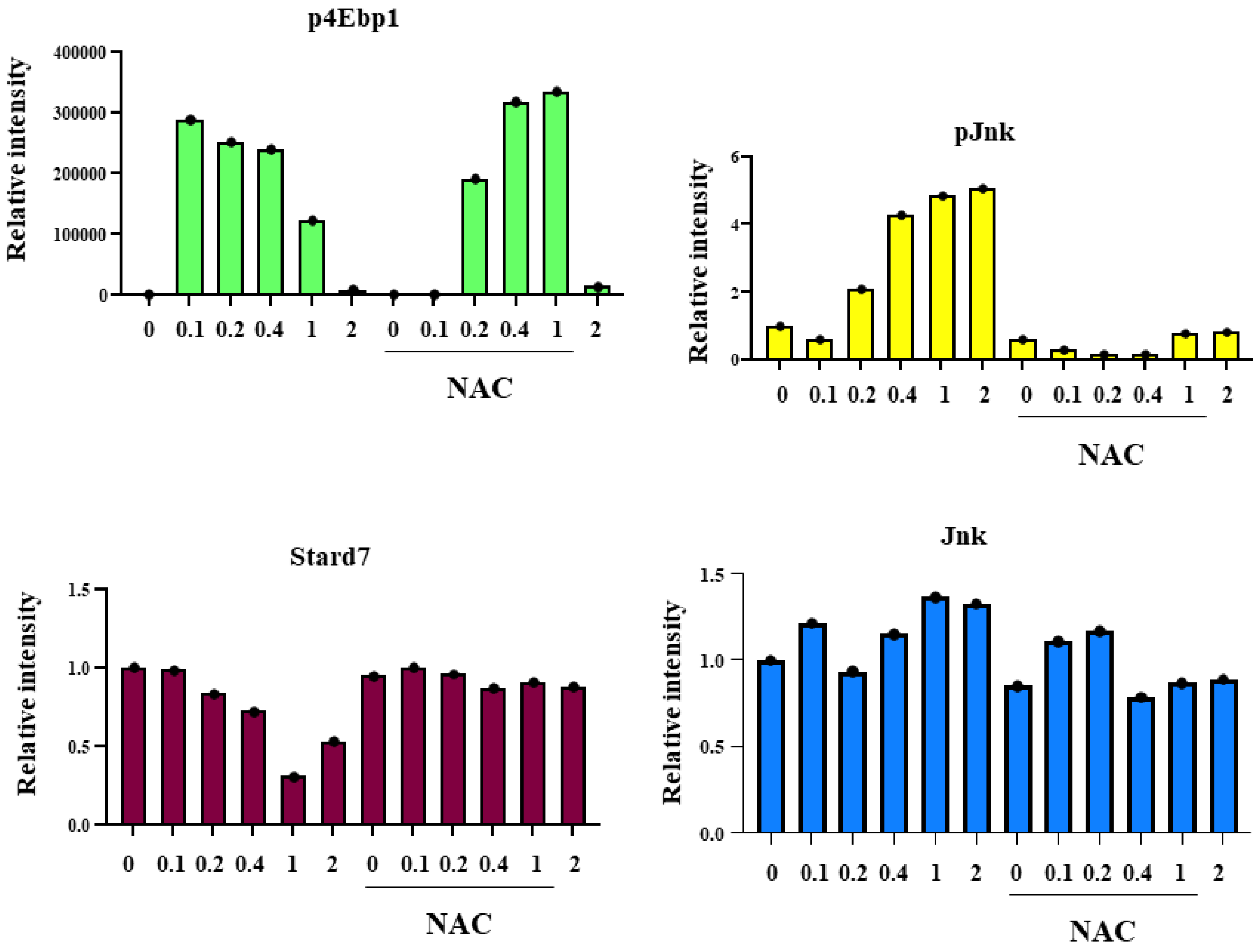
p4Ebp1
Relative intensity
400000
300000
200000
100000
0
0 0.1 0.2 0.4 1 2 0 0.1 0.2 0.4 1 2
NAC
pJnk
Relative intensity
6
4
2
0
0 0.1 0.2 0.4 1 2 0 0.1 0.2 0.4 1 2
NAC
Stard7
Relative intensity
1.5
1.0
0.5
0.0
0 0.1 0.2 0.4 1 2 0 0.1 0.2 0.4 1 2
NAC
Jnk
Relative intensity
1.5
1.0
0.5
0.0
0 0.1 0.2 0.4 1 2 0 0.1 0.2 0.4 1 2
NAC

Figure 3B

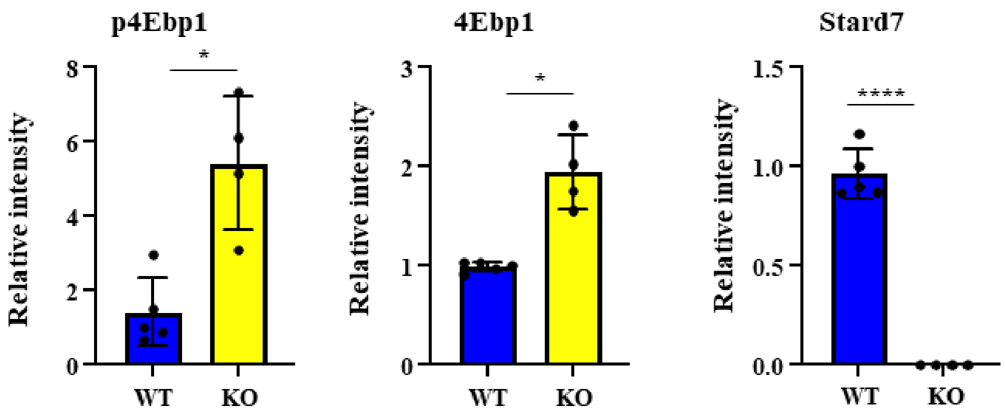

Figure 3C

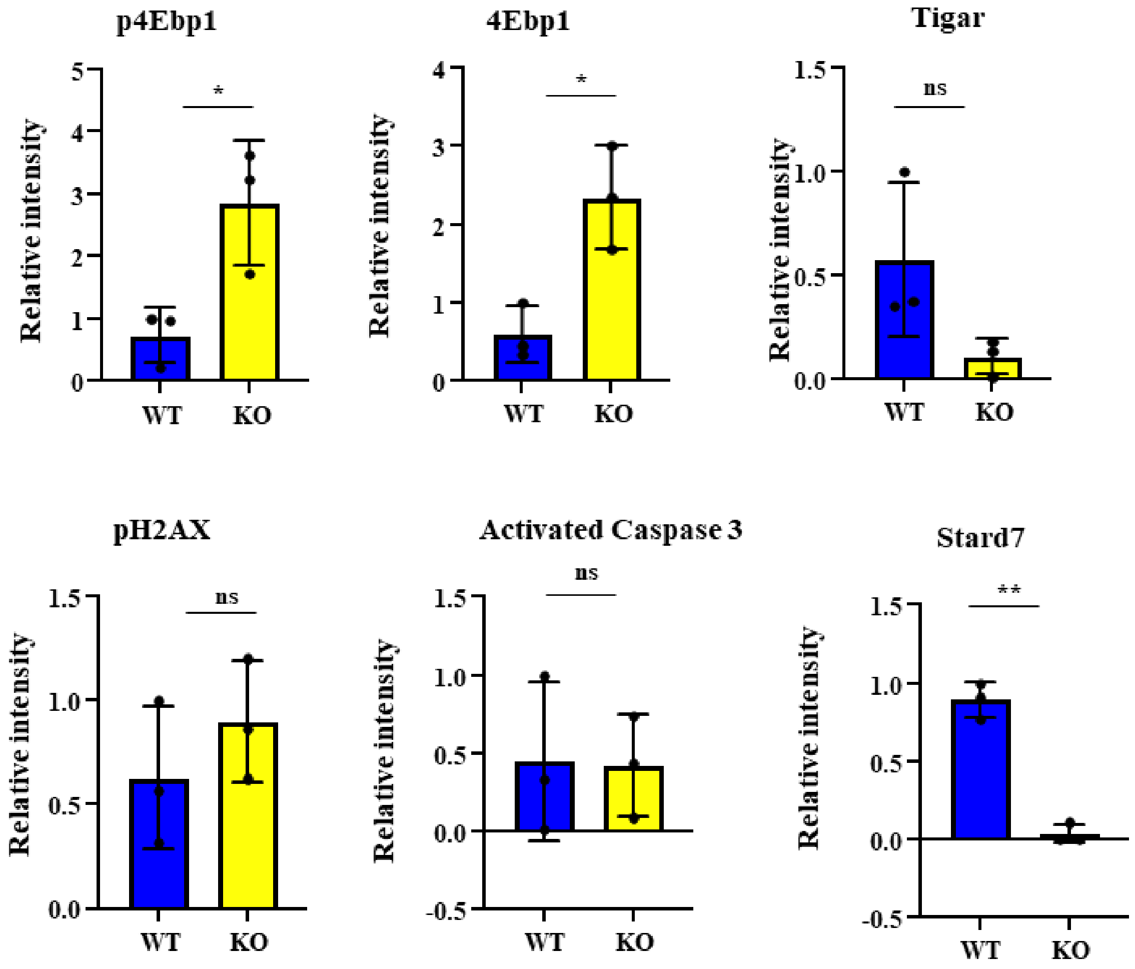

Figure 3D

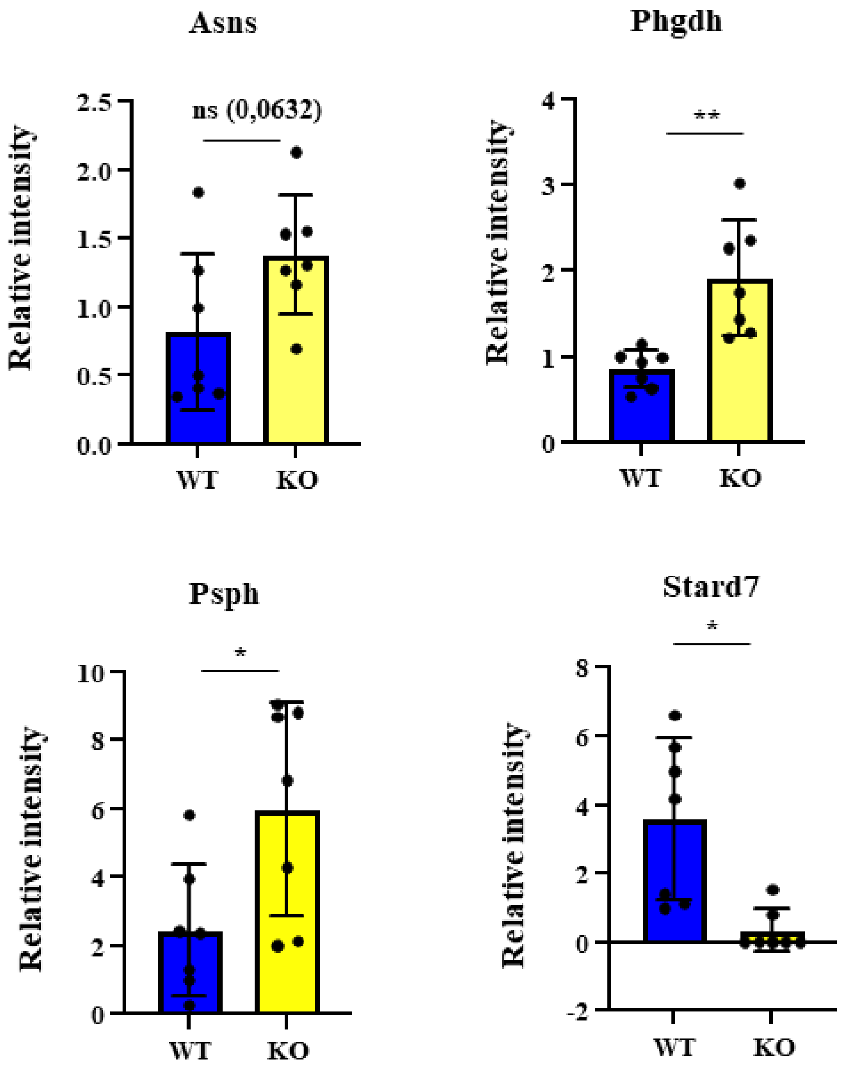
Asns
ns (0,0632)
Relative intensity
2.5
2.0
1.5
1.0
0.5
0.0
WT
KO
Phgdh
**
Relative intensity
4
3
2
1
0
WT
KO
Psph
*
Relative intensity
10
8
6
4
2
0
WT
KO
Stard7
*
Relative intensity
8
6
4
2
0
-2
WT
KO

Figure 5E

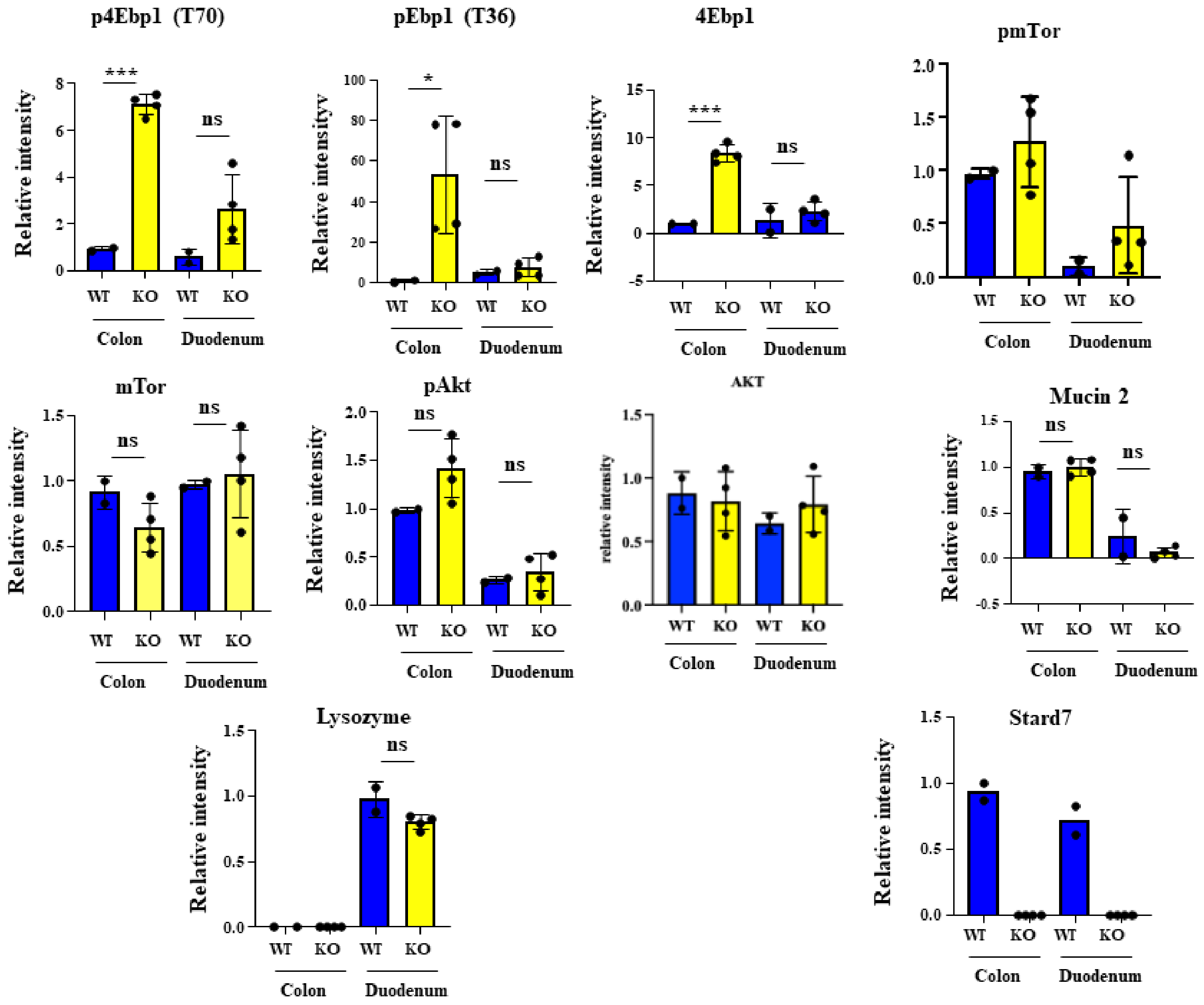

p4Ebp1 (T70)
pEbp1 (T36)
4Ebp1
pmTor
mTor
pAkt
AKT
Mucin 2
Lysozyme
Stard7
Relative intensity
Relative intensityy
relative intensity
***
*
ns
WT
KO
Colon
Duodenum

Figure 5F

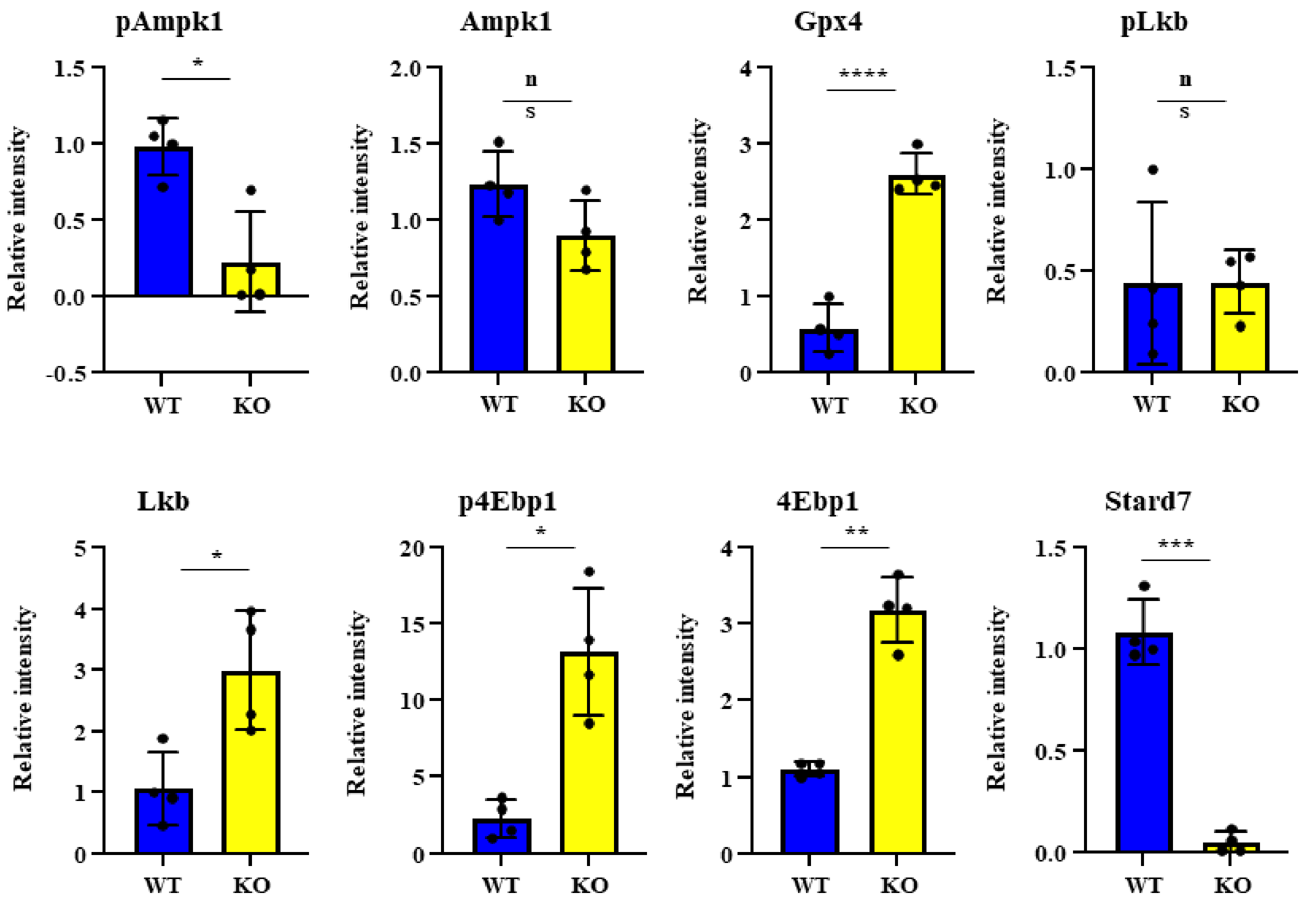
pAmpk1
Ampk1
Gpx4
pLkb
Lkb
p4Ebp1
4Ebp1
Stard7
Relative intensity
WT
KO
*
ns
****
**
***

Figure 6C

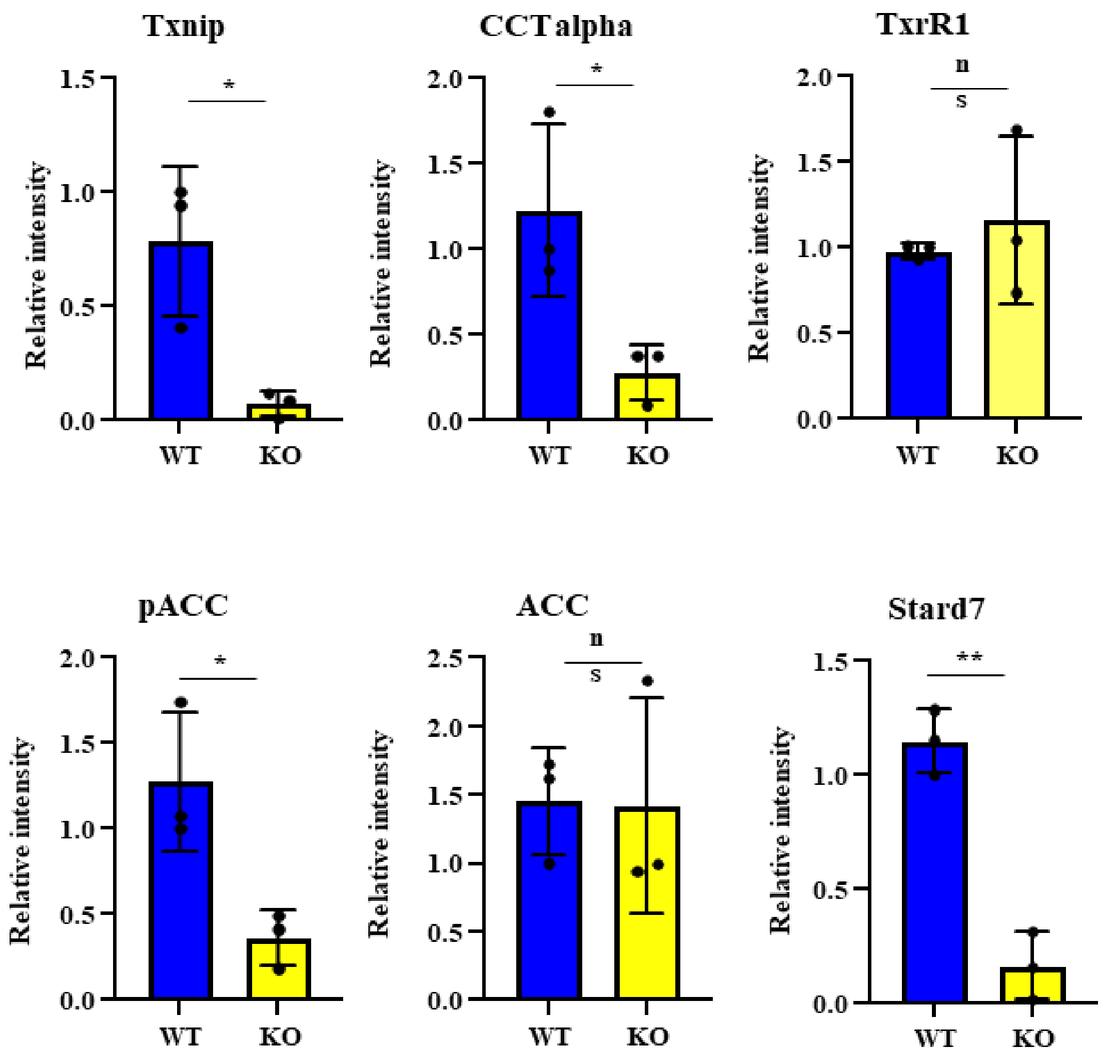

Txnip
CCTalpha
TxrR1
pACC
ACC
Stard7
Relative intensity
*
**
n
s
WT
KO
0.0
0.5
1.0
1.5
2.0
2.5

s

Figure 6E

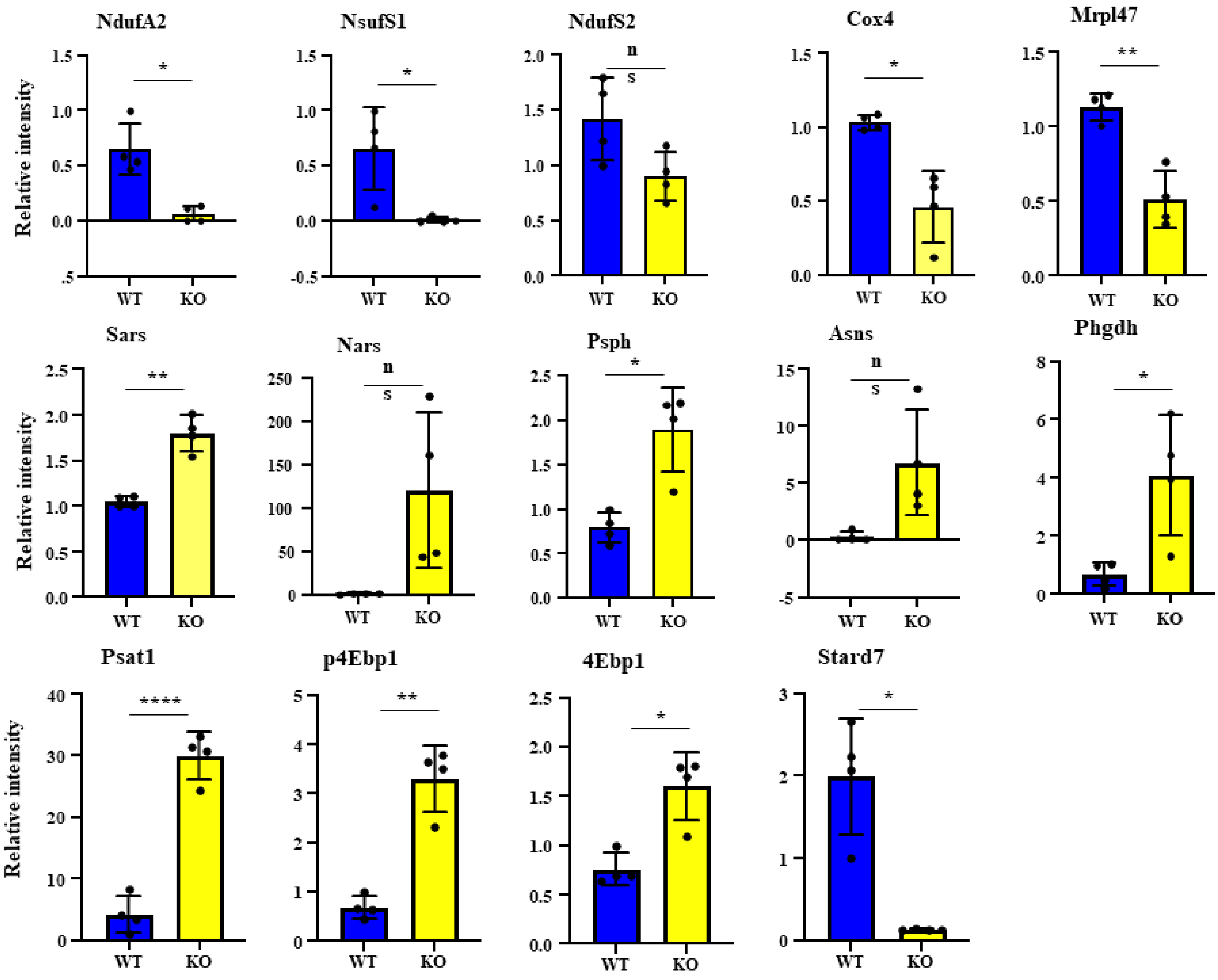
NdufA2
NsufS1
NdufS2
Cox4
Mrpl47
Sars
Nars
Psph
Asns
Phgdh
Psat1
p4Ebp1
4Ebp1
Stard7
Relative intensity
WT
KO

Figure 7B

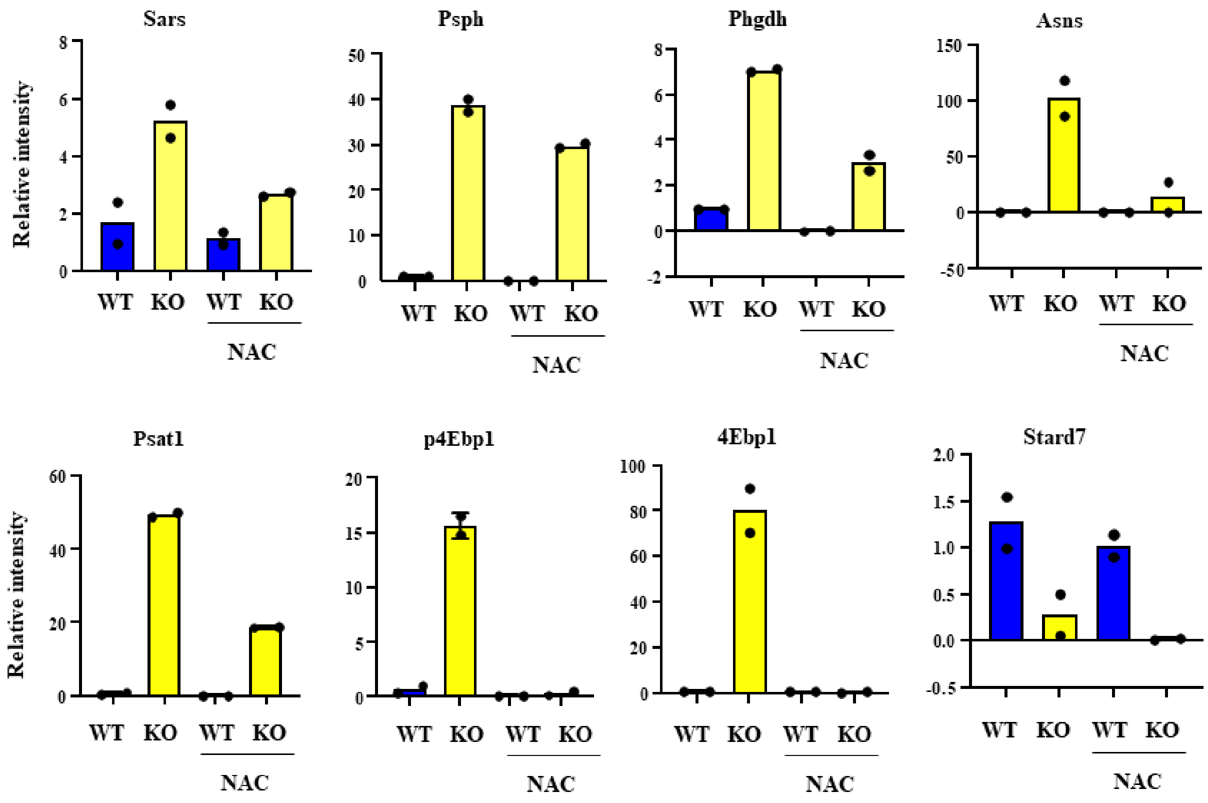
Sars
Psph
Phgdh
Asns
Psat1
p4Ebp1
4Ebp1
Stard7
Relative intensity
WT
KO
WT
KO
NAC

Figure 7C

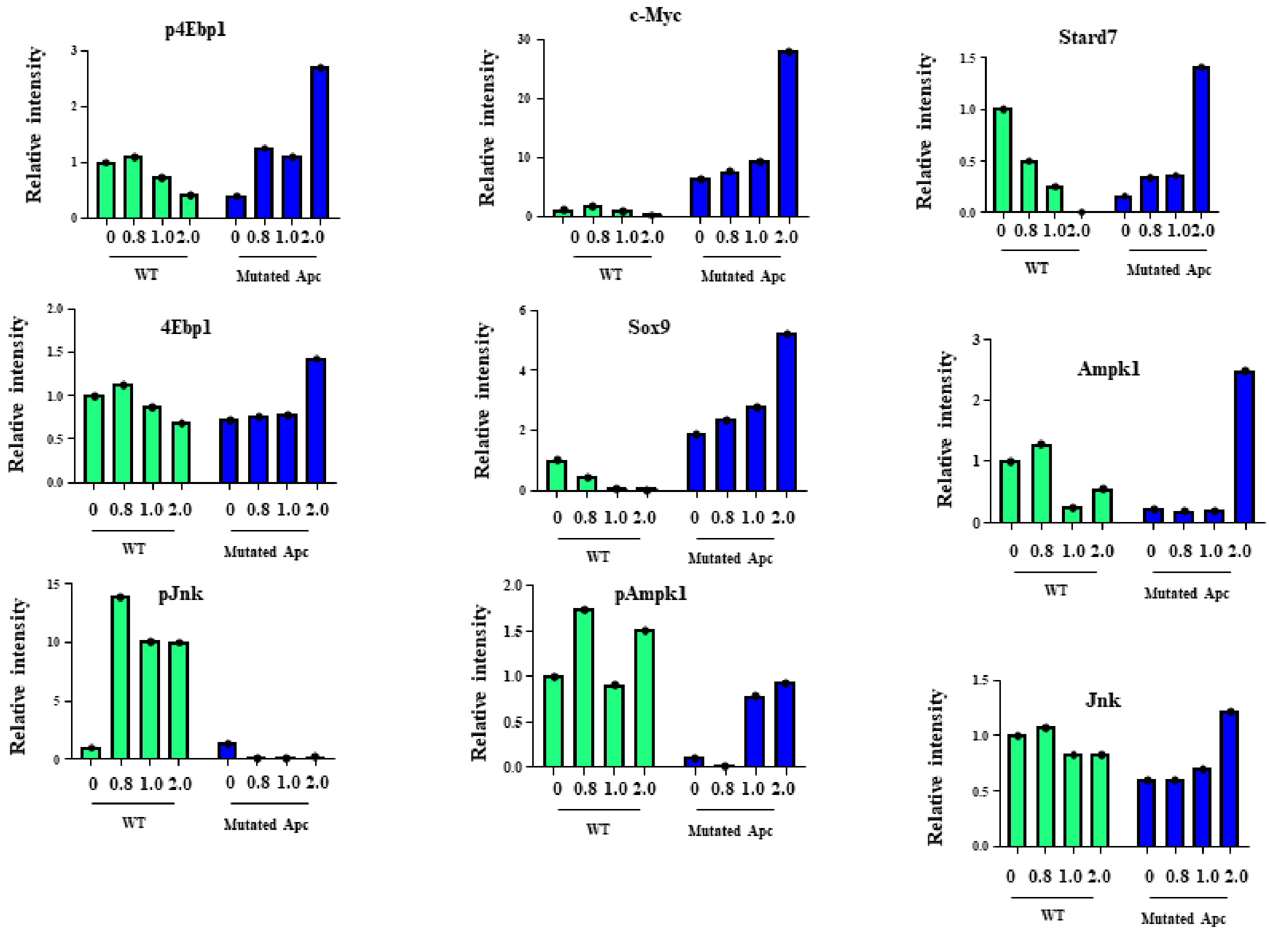
p4Ebp1
c-Myc
Stard7
4Ebp1
Sox9
Ampk1
pJnk
pAmpk1
Jnk
Relative intensity
0 0.8 1.0 2.0
WT
Mutated Apc

Figure EV1E

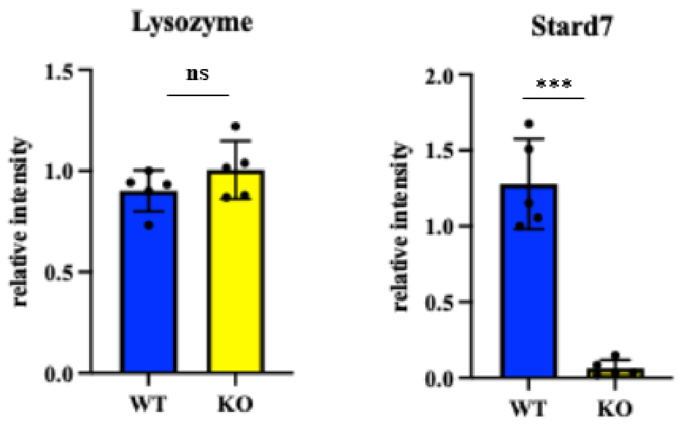

Figure EV2B

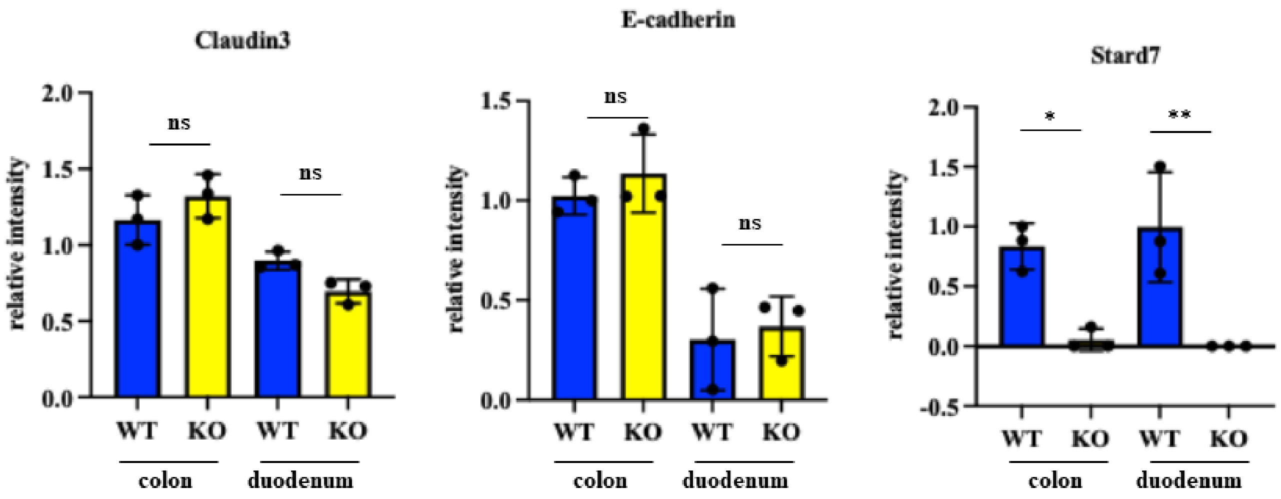

Figure EV3B

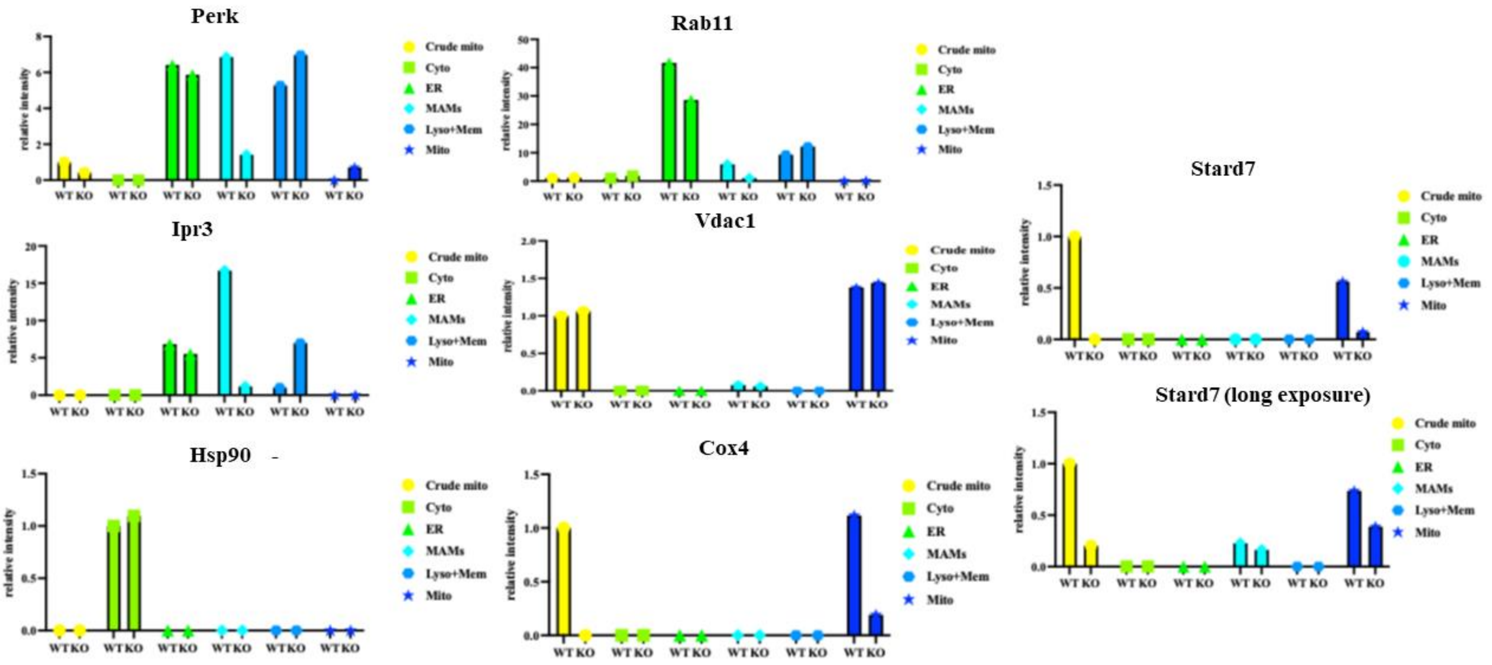

Perk
Rab11
Stard7
Ipr3
Vdac1
Stard7 (long exposure)
Hsp90
Cox4
relative intensity
WT KO
Crude mito
Cyto
ER
MAMs
Lyso+Mem
Mito

Figure EV4D

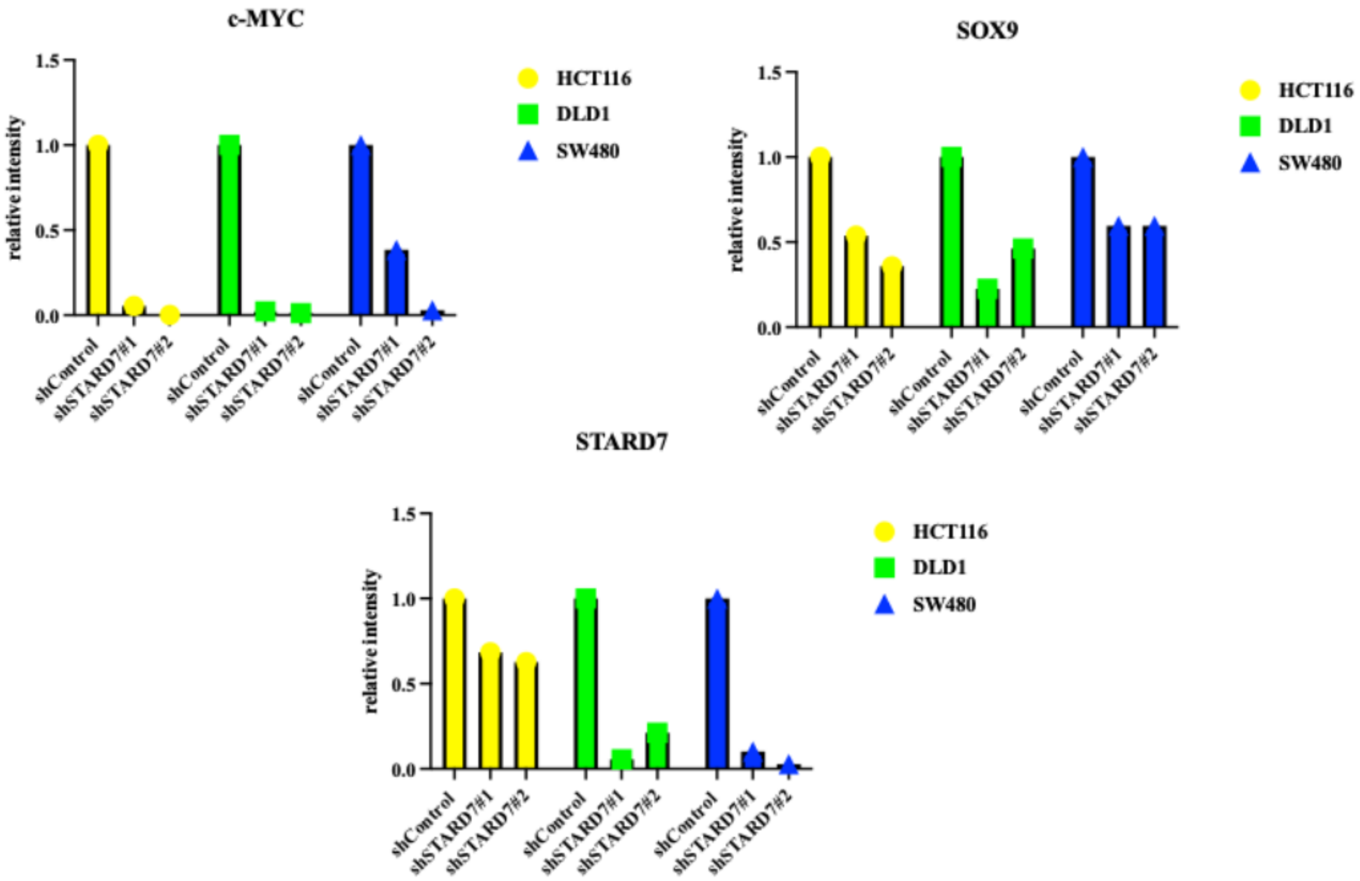

Figure EV4E

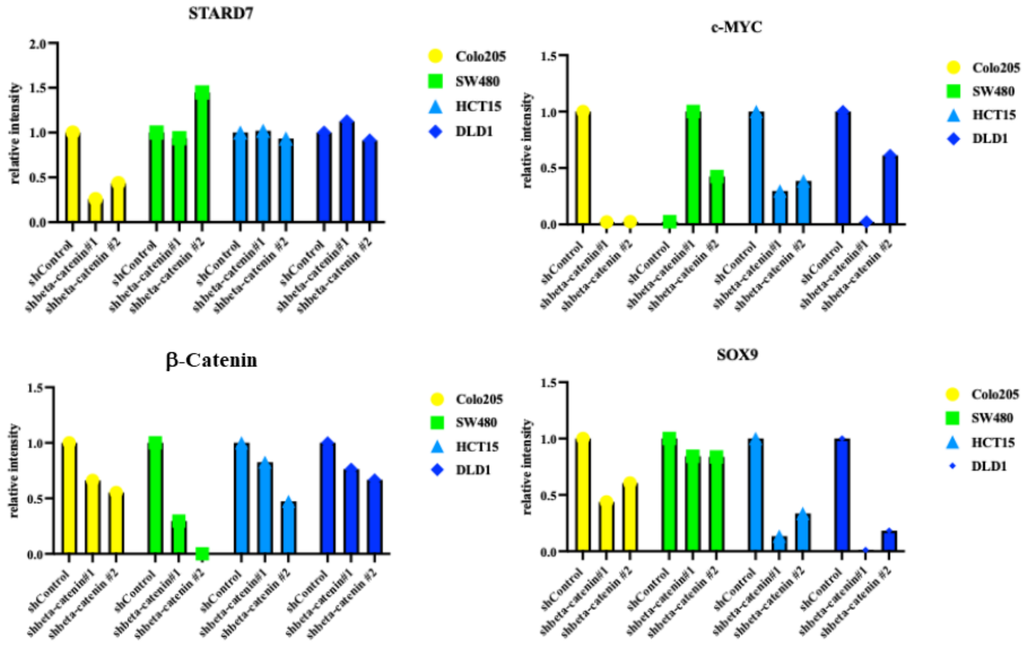

Figure EV4F

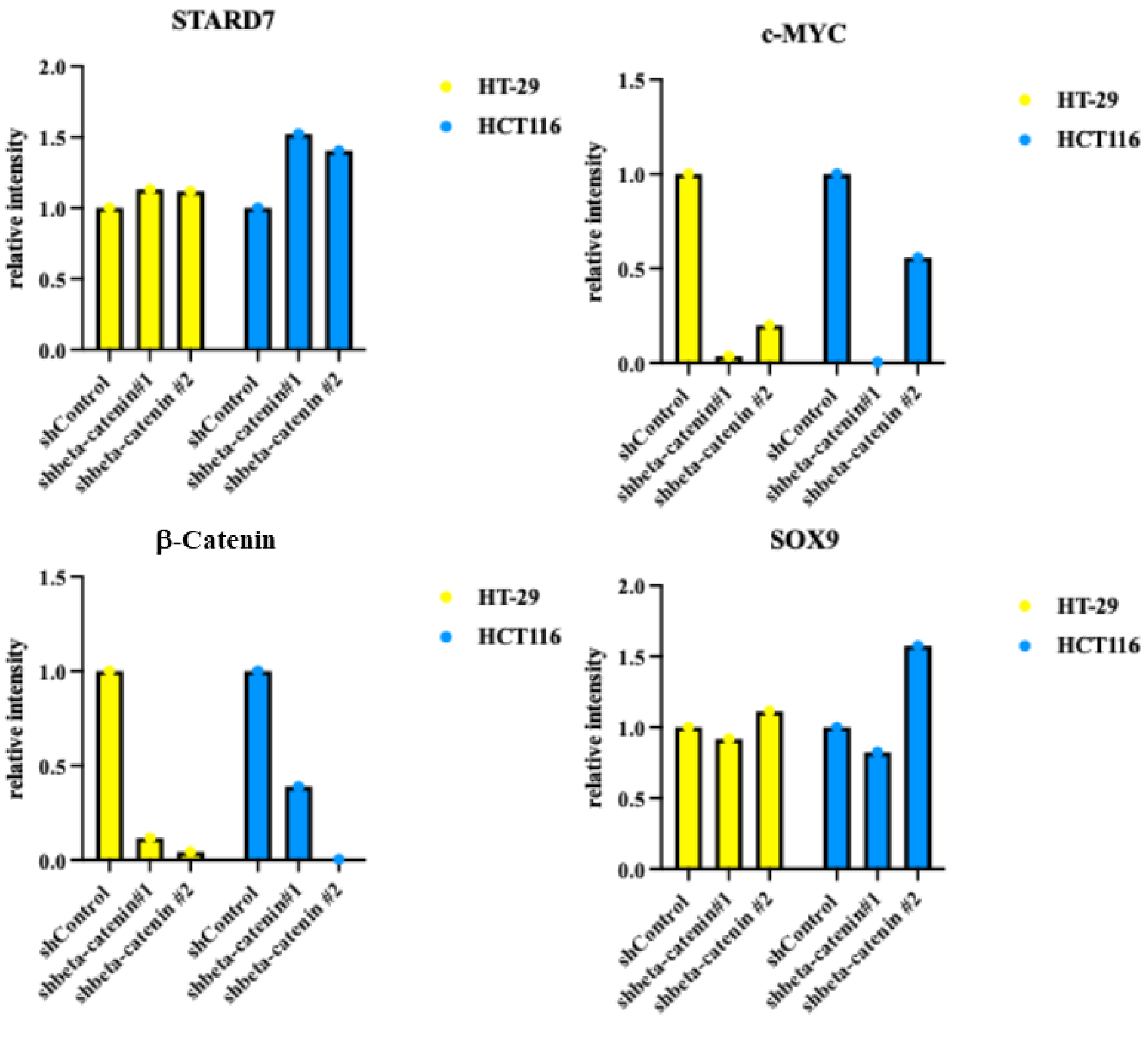

STARD7
relative intensity
2.0
1.5
1.0
0.5
0.0
shControl
shbeta-catenin#1
shbeta-catenin #2
shControl
shbeta-catenin#1
shbeta-catenin #2
HT-29
HCT116
c-MYC
relative intensity
1.5
1.0
0.5
0.0
shControl
shbeta-catenin#1
shbeta-catenin #2
shControl
shbeta-catenin#1
shbeta-catenin #2
HT-29
HCT116
β-Catenin
relative intensity
1.5
1.0
0.5
0.0
shControl
shbeta-catenin#1
shbeta-catenin #2
shControl
shbeta-catenin#1
shbeta-catenin #2
HT-29
HCT116
SOX9
relative intensity
2.0
1.5
1.0
0.5
0.0
shControl
shbeta-catenin#1
shbeta-catenin #2
shControl
shbeta-catenin#1
shbeta-catenin #2
HT-29
HCT116

Figure EV4G

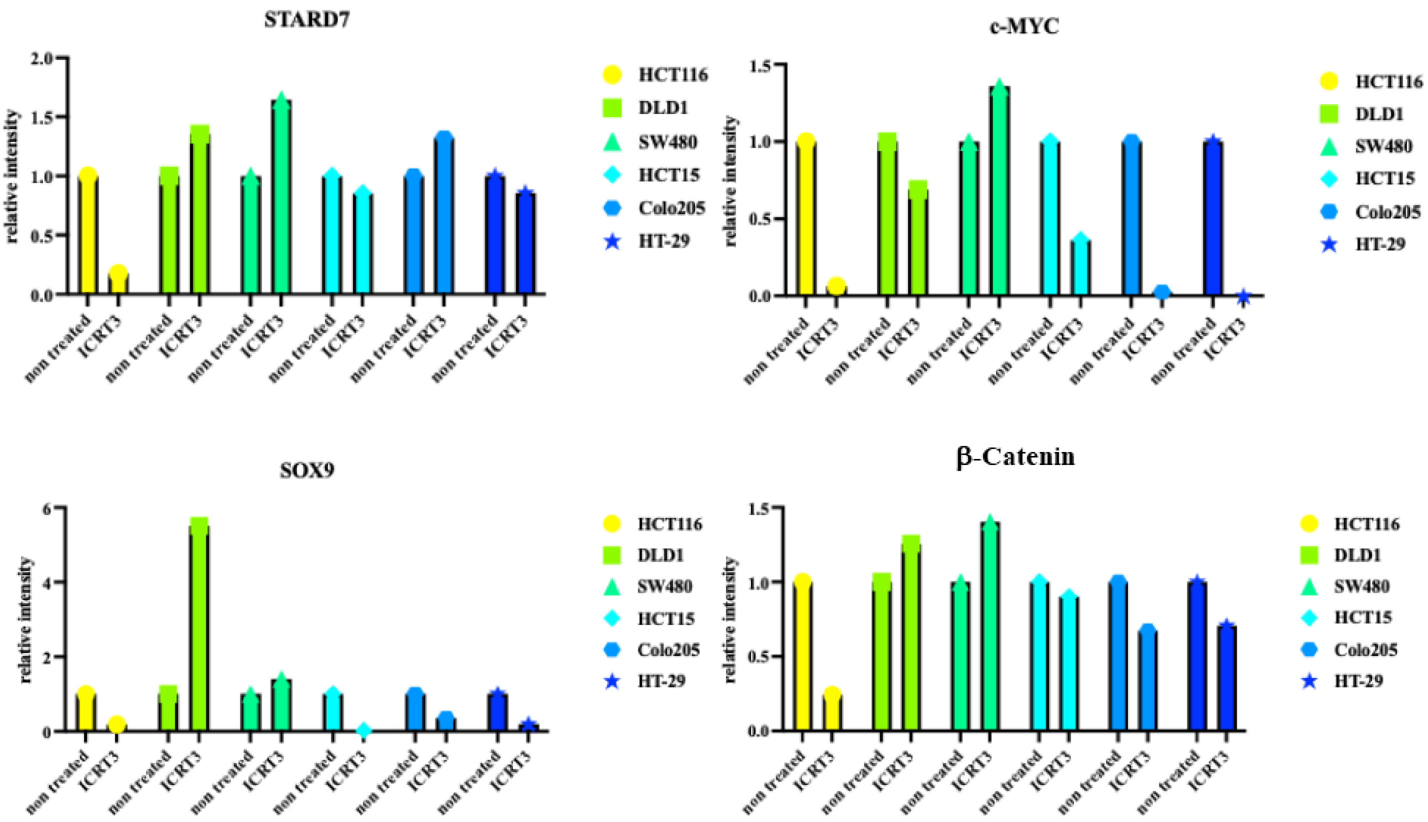

Figure EV4H

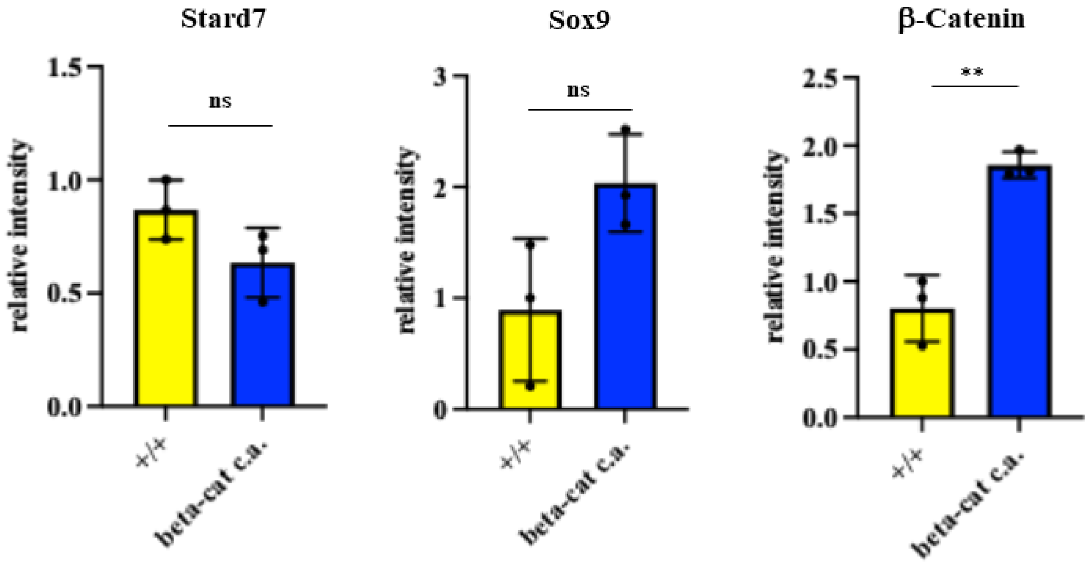

Figure EV5A

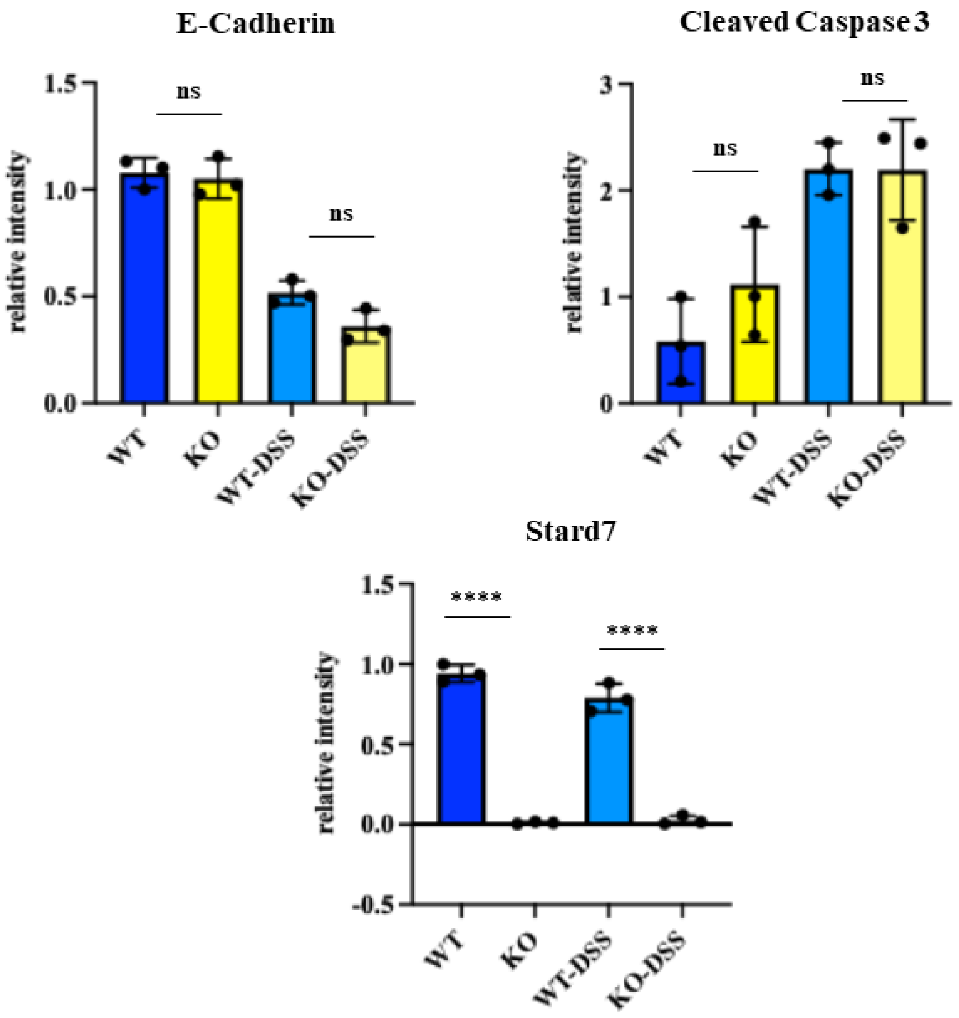

E-Cadherin
relative intensity
1.5
1.0
0.5
0.0
ns
ns
WT
KO
WT-DSS
KO-DSS
Cleaved Caspase 3
relative intensity
3
2
1
0
ns
ns
WT
KO
WT-DSS
KO-DSS
Stard7
relative intensity
1.5
1.0
0.5
0.0
-0.5
****
****
WT
KO
WT-DSS
KO-DSS

Figure EV8A

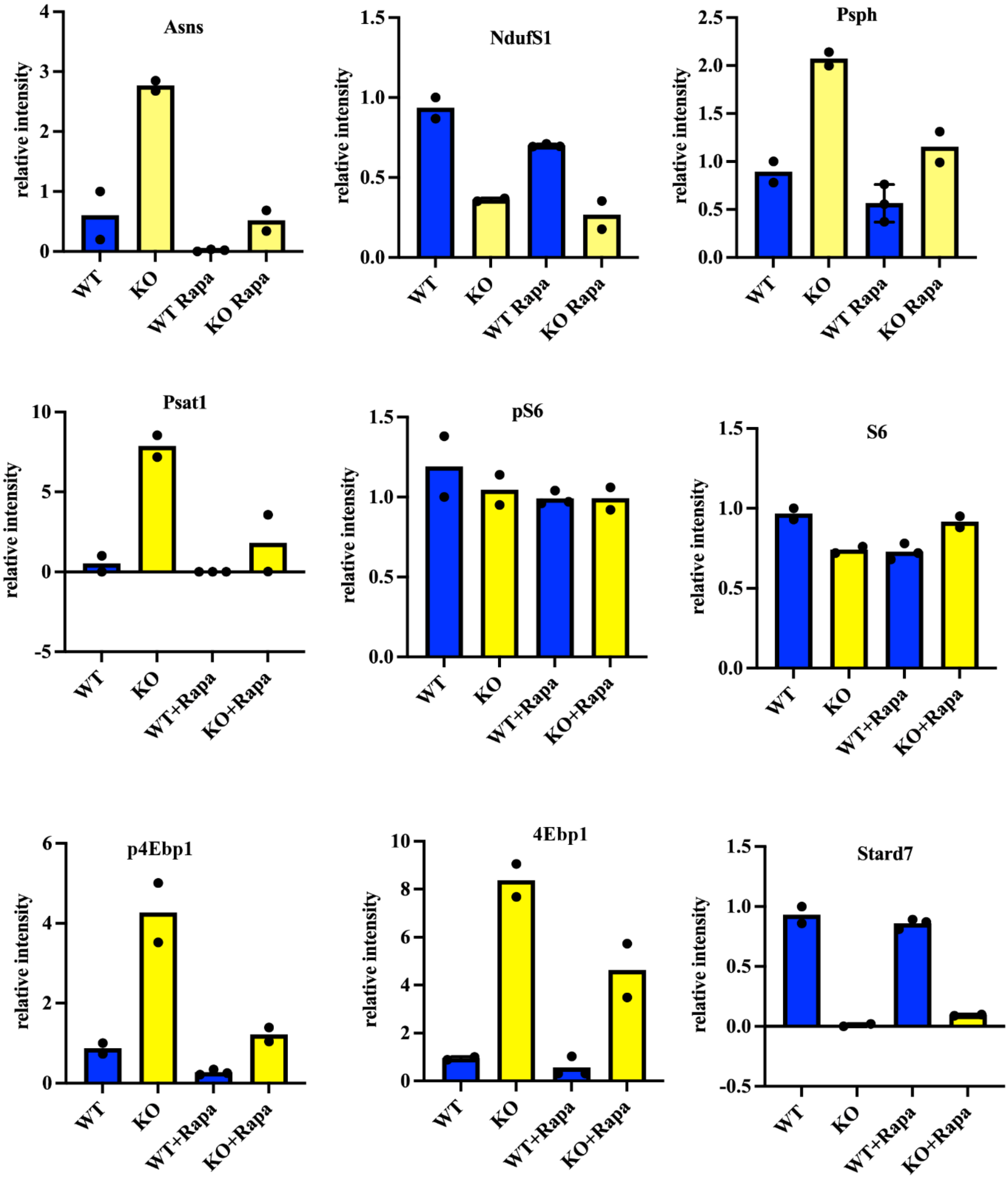
Asns
NdufS1
Psph
Psat1
pS6
S6
p4Ebp1
4Ebp1
Stard7
relative intensity
WT
KO
WT Rapa
KO Rapa
WT+Rapa
KO+Rapa

Figure EV9

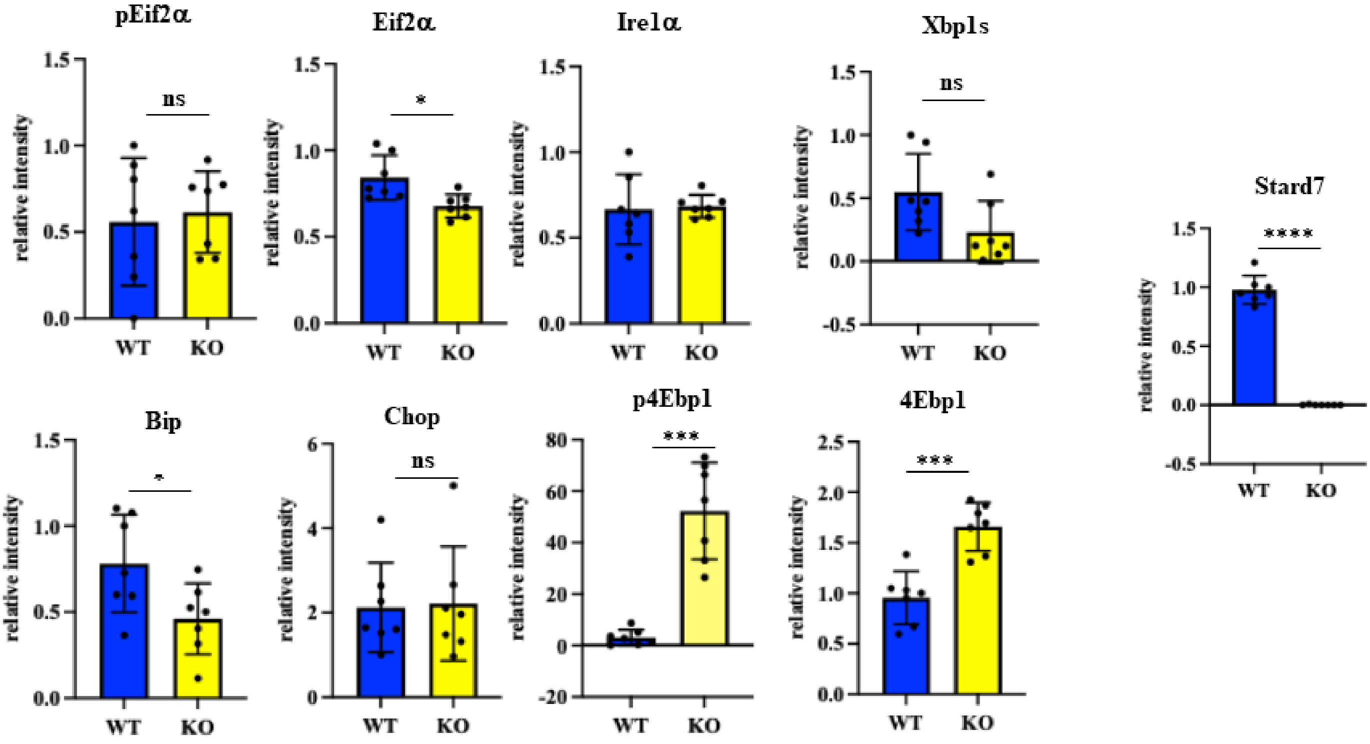

pEif2α
Eif2α
Ire1α
Xbp1s
Stard7
Bip
Chop
p4Ebp1
4Ebp1
relative intensity
WT
KO
ns
*
***
****
