# Supplementary material for: The lipid transfer protein STARD7 controls intestinal tumor development in a context-dependent manner
Source: EMBO Mol Med. 2026 Mar 30;18(5):1771–811. doi: 10.1038/s44321-026-00409-5 (PMC13179355; doi:10.1038/s44321-026-00409-5)
Supplement: Supplementary file 6 — Source data Fig. 1 [file 44321_2026_409_MOESM6_ESM.zip › Fig1/Fig1I/Fig1I.pptx]

## Slide 1
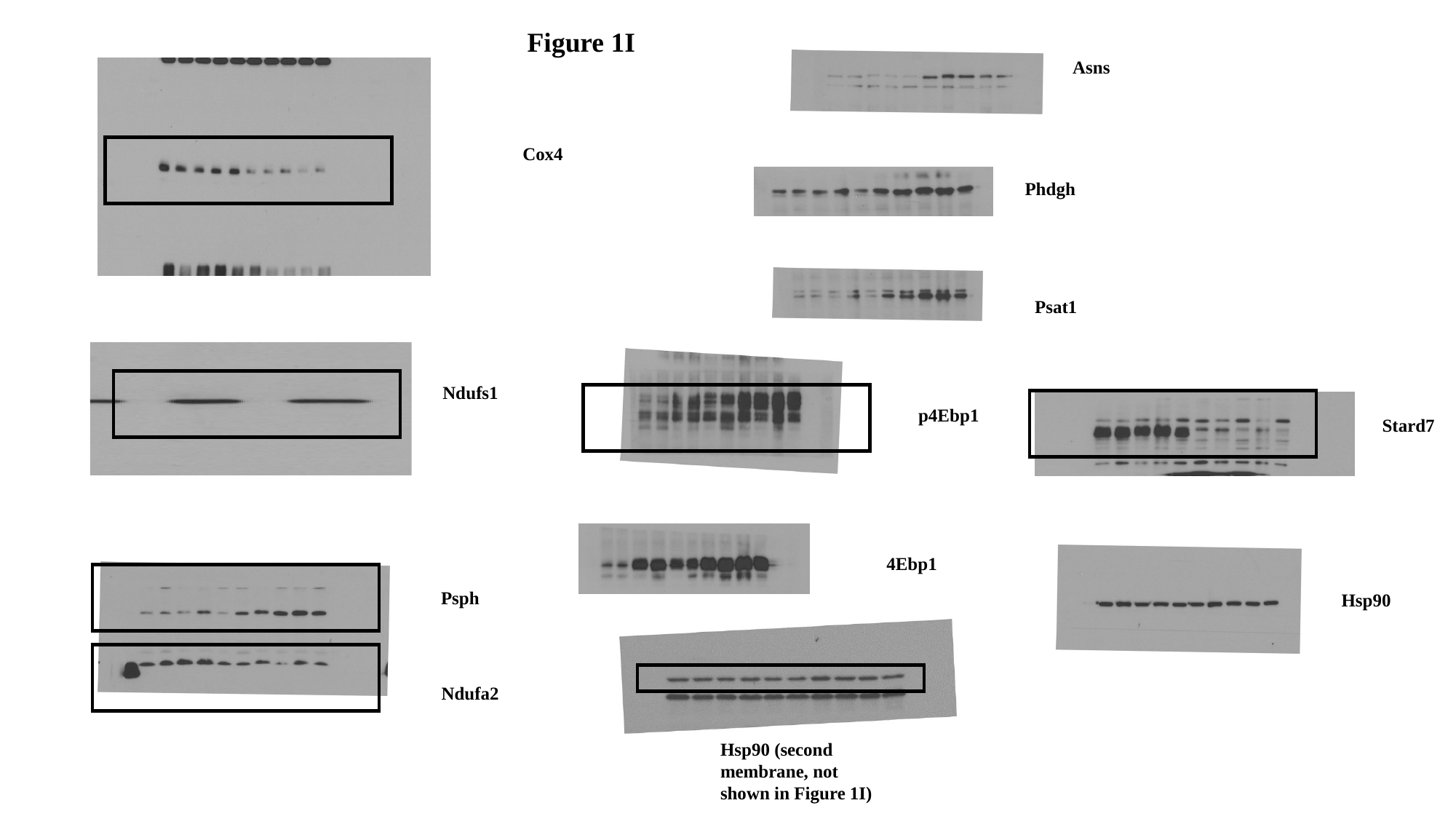

Figure 1I
Asns
Cox4
Phdgh
Psat1
Ndufs1
p4Ebp1
Stard7
4Ebp1
Psph
Hsp90
Ndufa2
Hsp90 (second membrane, not shown in Figure 1I)
