# Supplementary figures and images for: The lipid transfer protein STARD7 controls intestinal tumor development in a context-dependent manner
Source: EMBO Mol Med. 2026 Mar 30;18(5):1771–811. doi: 10.1038/s44321-026-00409-5 (PMC13179355; doi:10.1038/s44321-026-00409-5)

## Slide 1
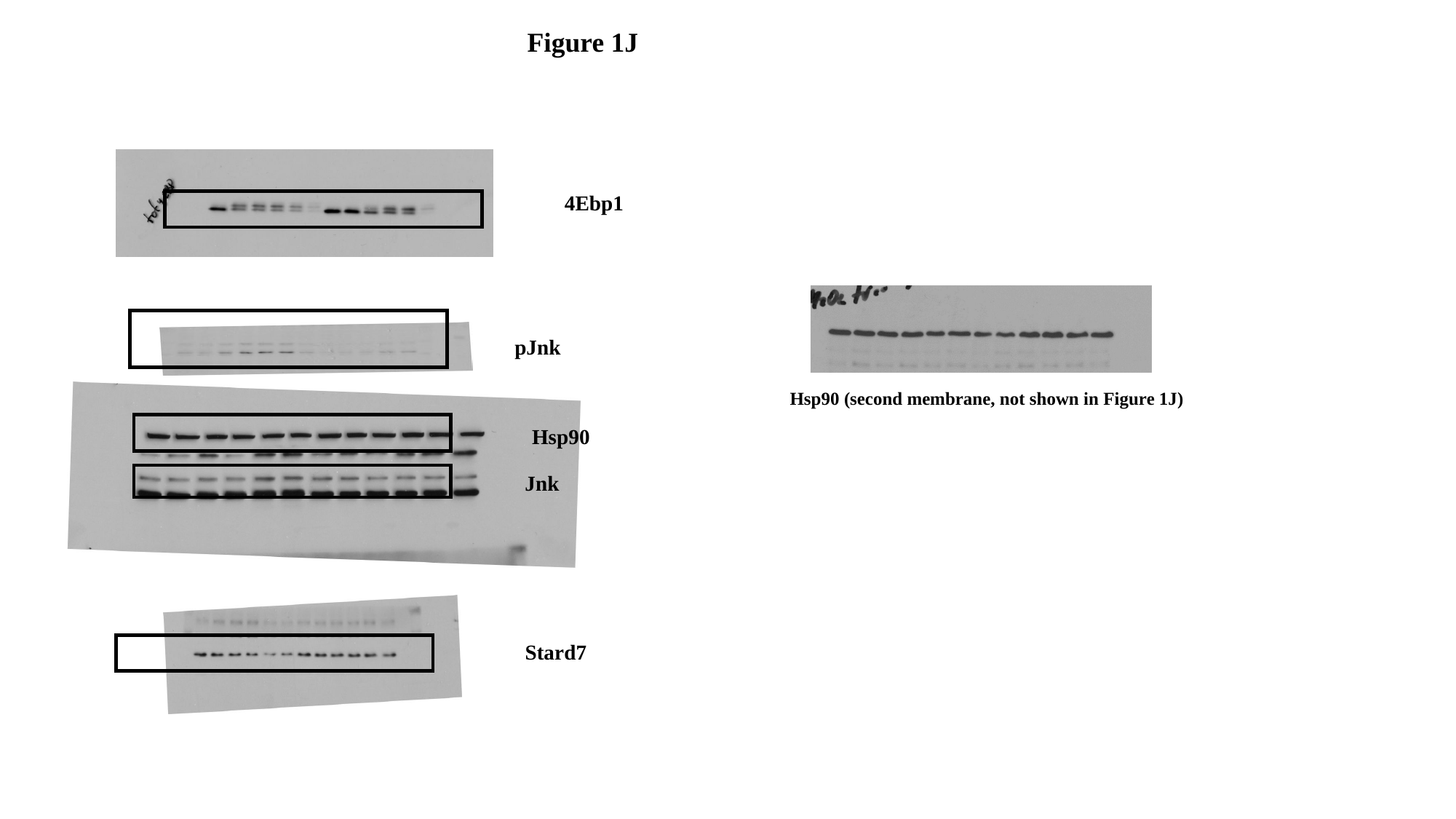

Figure 1J
4Ebp1
pJnk
Hsp90 (second membrane, not shown in Figure 1J)
Hsp90
Jnk
Stard7

Supplement: Supplementary file 6 — Source data Fig. 1 [file 44321_2026_409_MOESM6_ESM.zip › Fig1/Fig1J/Fig1J.pptx]

## Slide 1
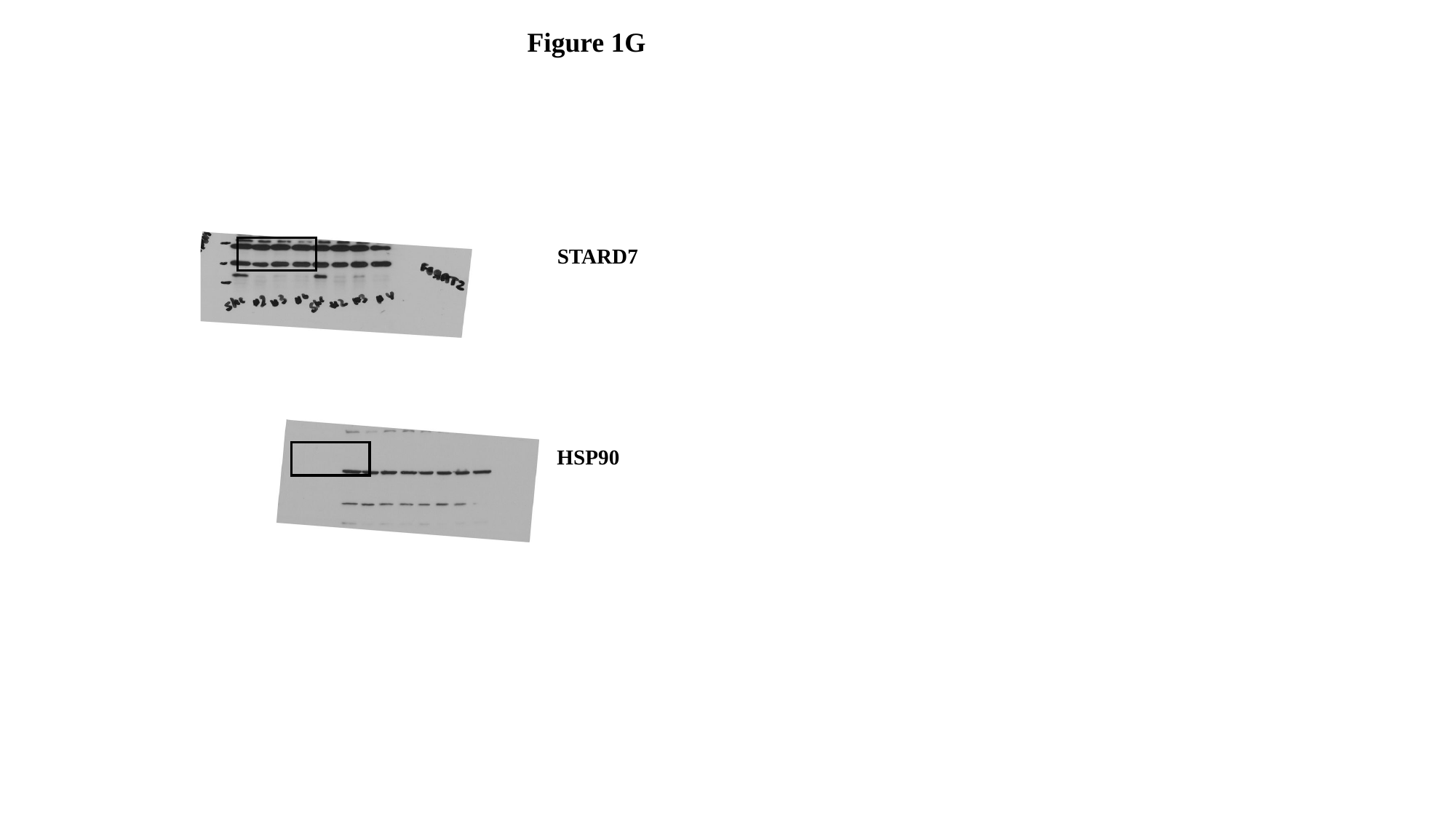

Figure 1G
STARD7
HSP90

Supplement: Supplementary file 6 — Source data Fig. 1 [file 44321_2026_409_MOESM6_ESM.zip › Fig1/Fig1G/Fig1G.pptx]

## Slide 1
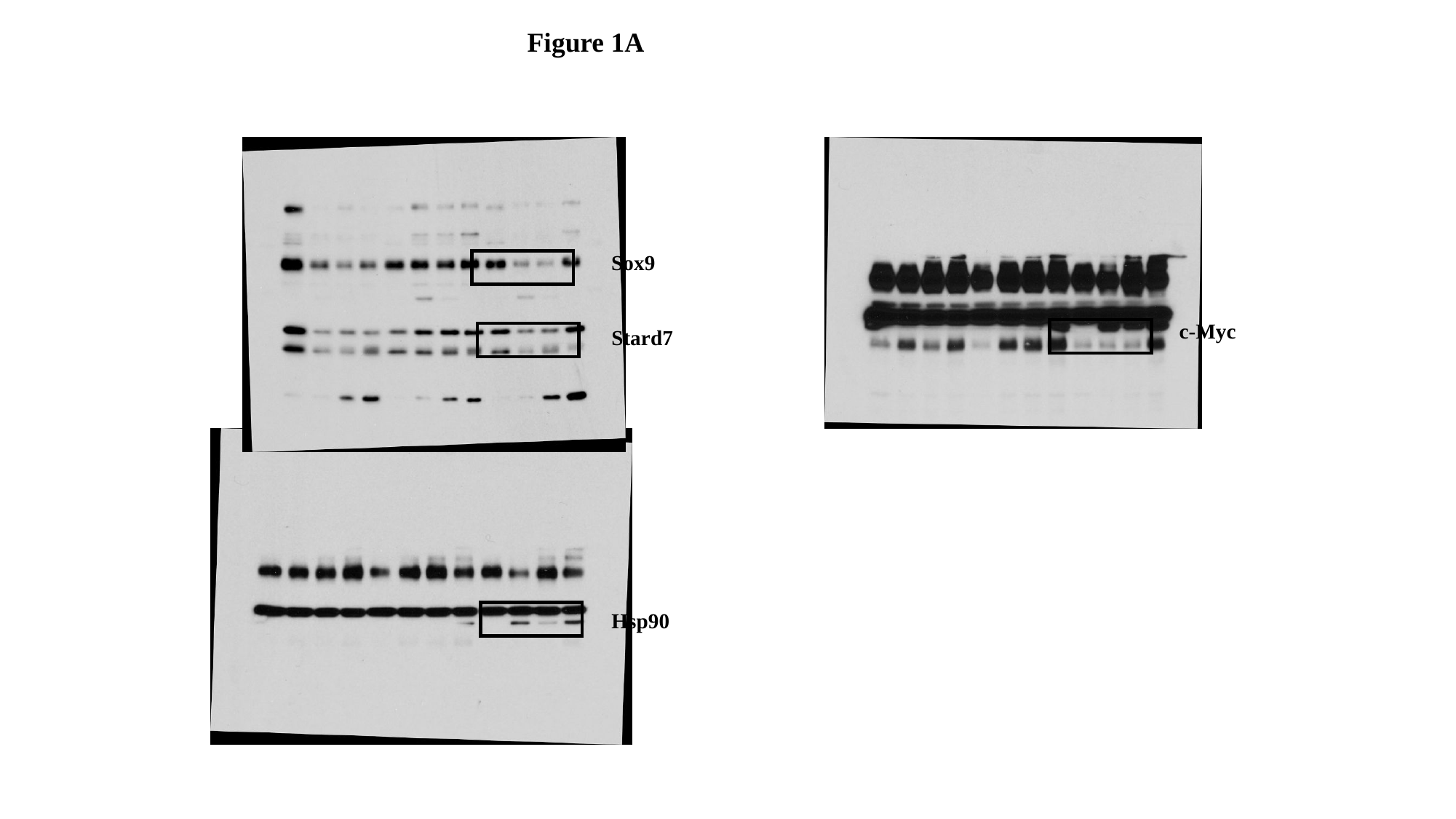

Figure 1A
Sox9
c-Myc
Stard7
Hsp90

Supplement: Supplementary file 6 — Source data Fig. 1 [file 44321_2026_409_MOESM6_ESM.zip › Fig1/Fig1A/Fig1A.pptx]

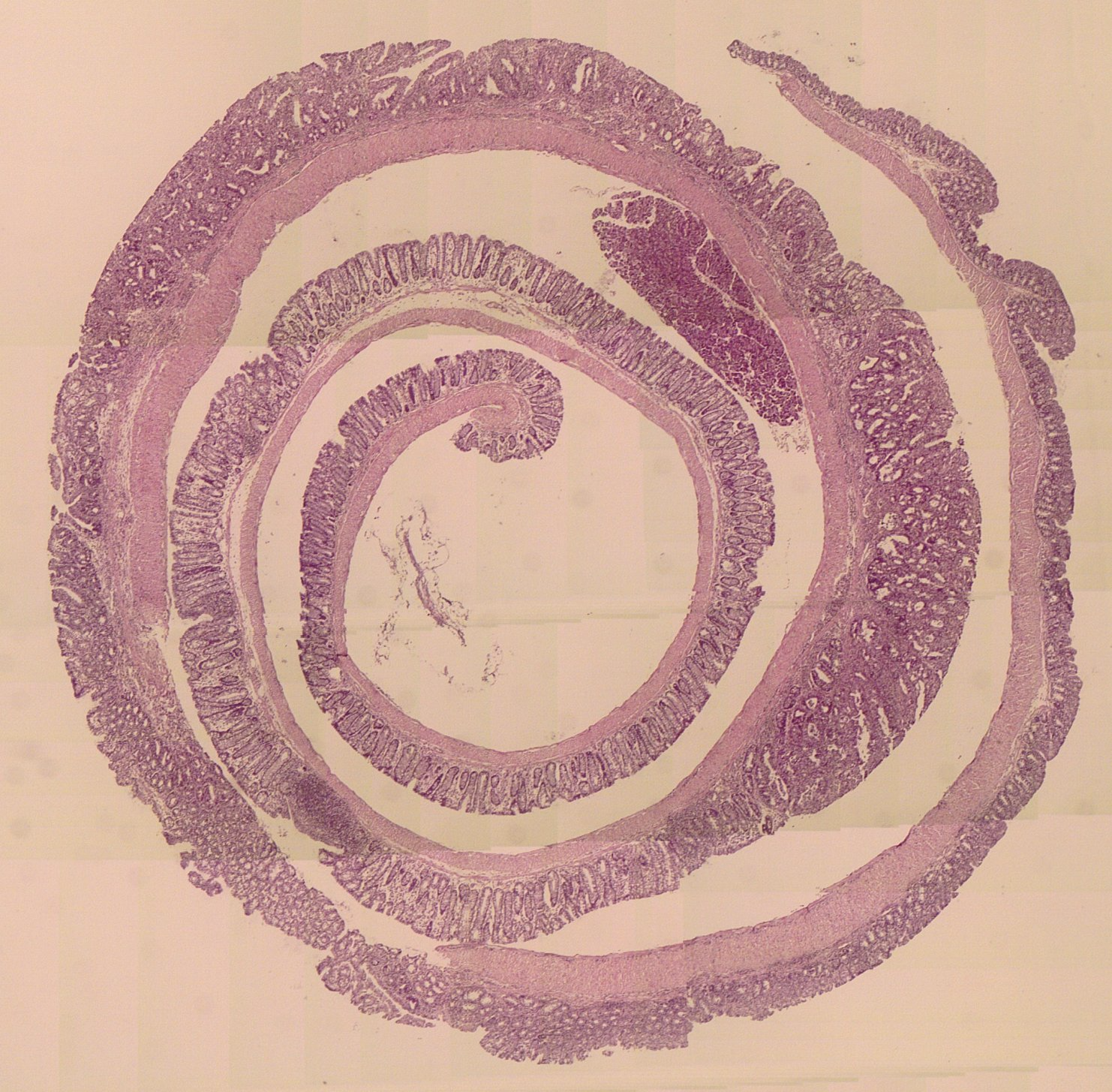

Supplement: Supplementary file 7 — Source data Fig. 2 [file 44321_2026_409_MOESM7_ESM.zip › Fig2/Fig2A/Fig2A IHC/Figure 2A KO.tiff]

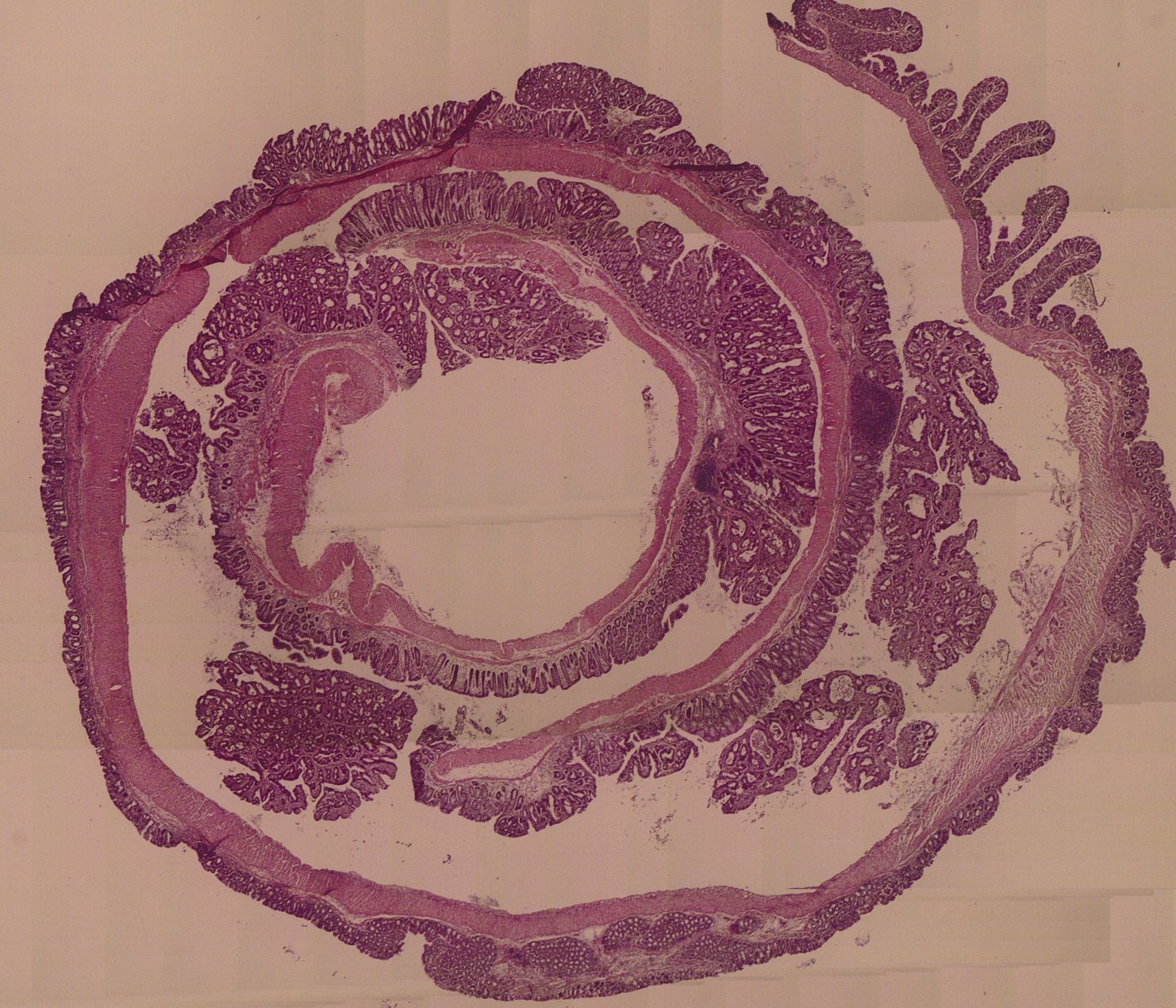

Supplement: Supplementary file 7 — Source data Fig. 2 [file 44321_2026_409_MOESM7_ESM.zip › Fig2/Fig2A/Fig2A IHC/Figure 2A WT.tiff]

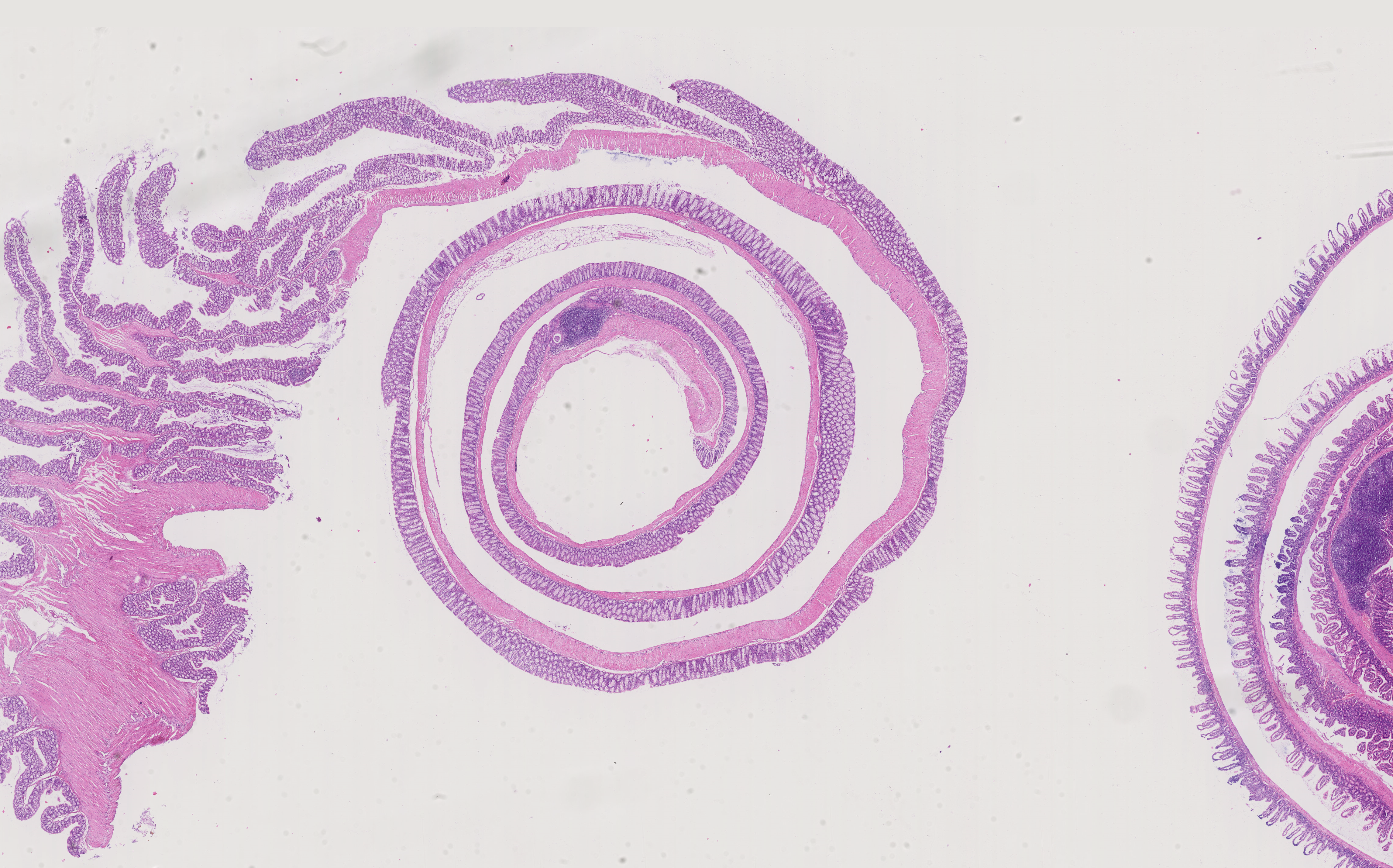

Supplement: Supplementary file 7 — Source data Fig. 2 [file 44321_2026_409_MOESM7_ESM.zip › Fig2/Fig2B/Fig 2B IHC/Fig2B KO.tif]

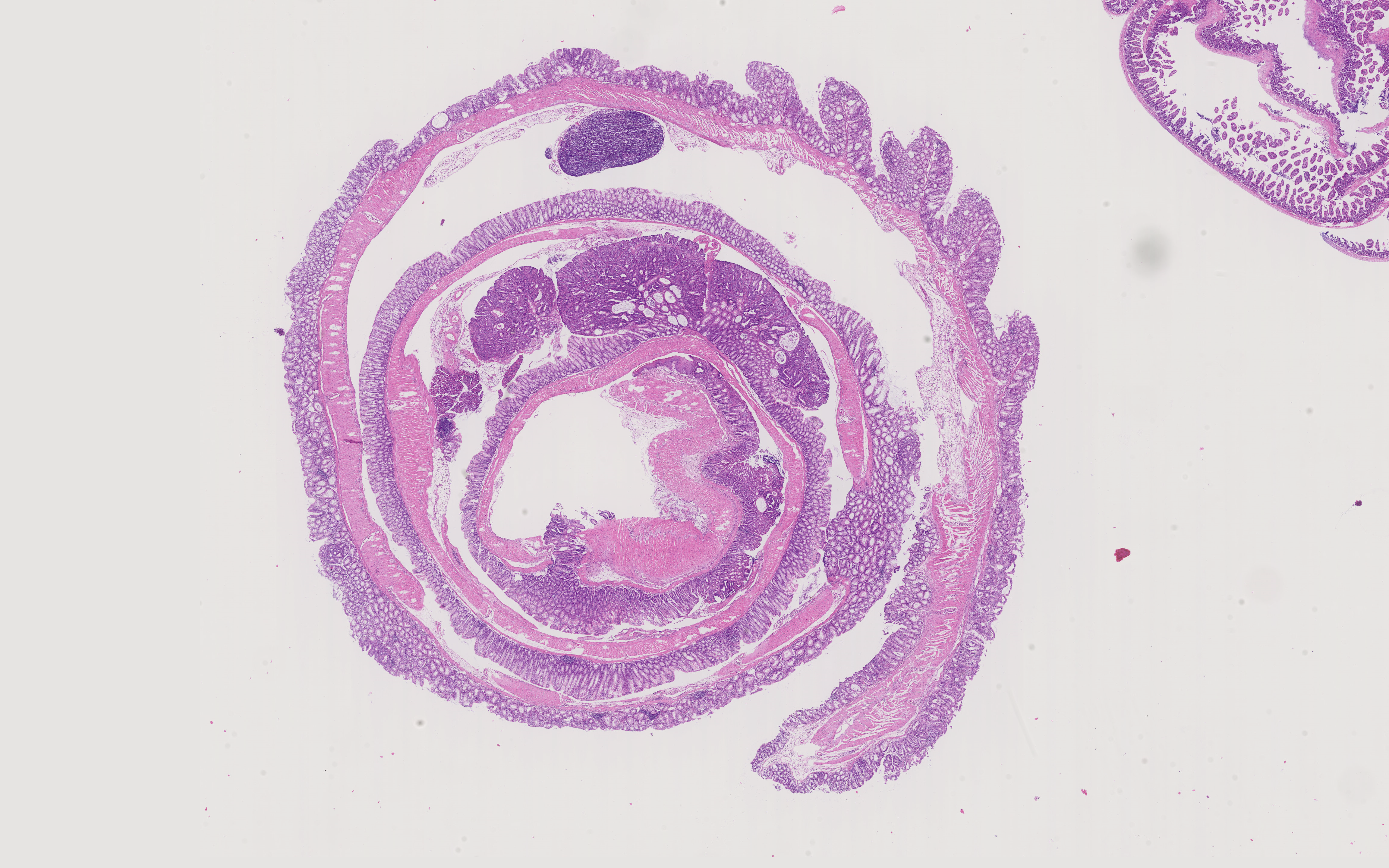

Supplement: Supplementary file 7 — Source data Fig. 2 [file 44321_2026_409_MOESM7_ESM.zip › Fig2/Fig2B/Fig 2B IHC/Fig2B WT.tif]

## Slide 1
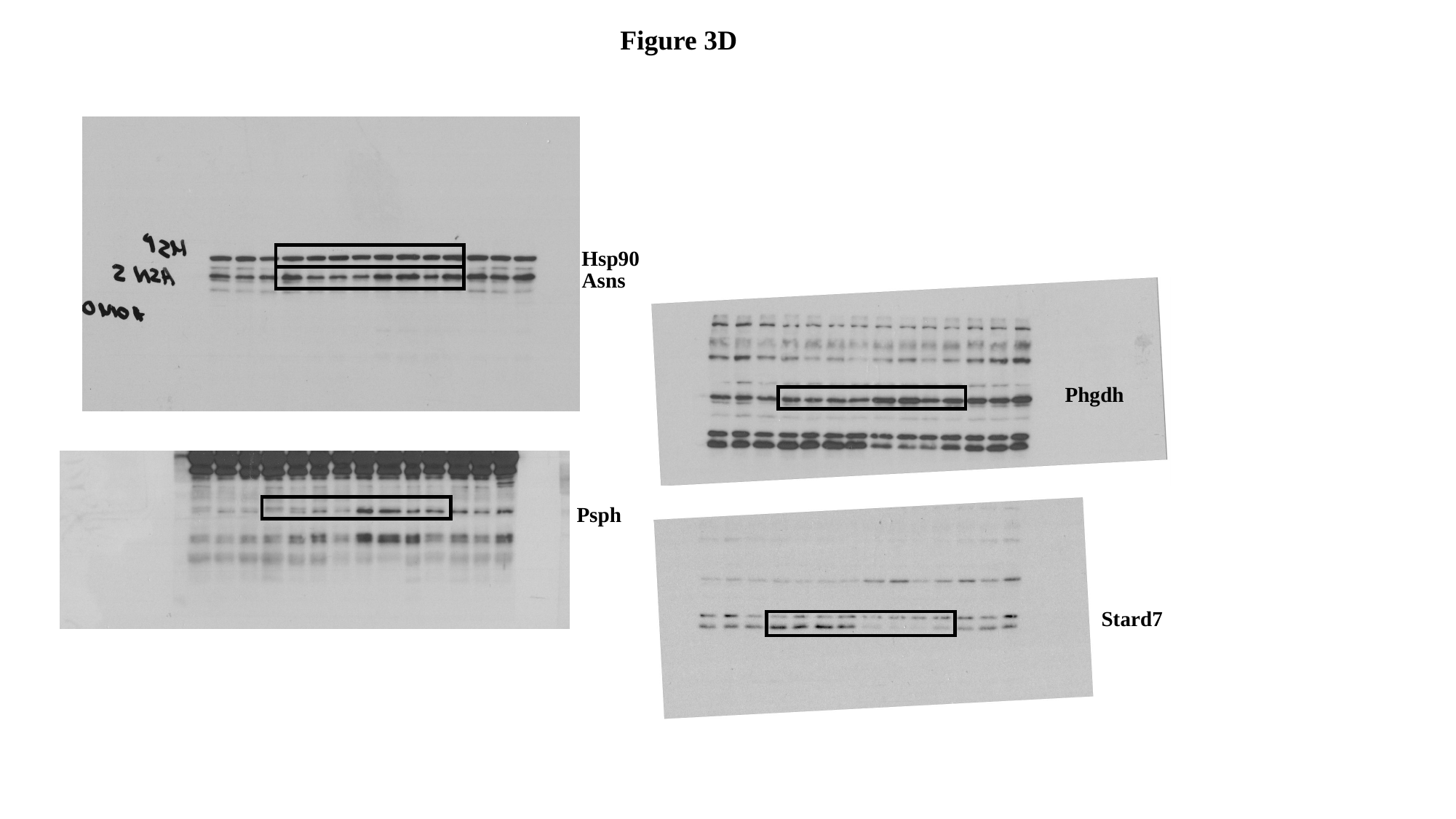

Figure 3D
Hsp90
Asns
Phgdh
Psph
Stard7

Supplement: Supplementary file 8 — Source data Fig. 3 [file 44321_2026_409_MOESM8_ESM.zip › Fig3/Fig3D/Fig3D.pptx]

## Slide 1
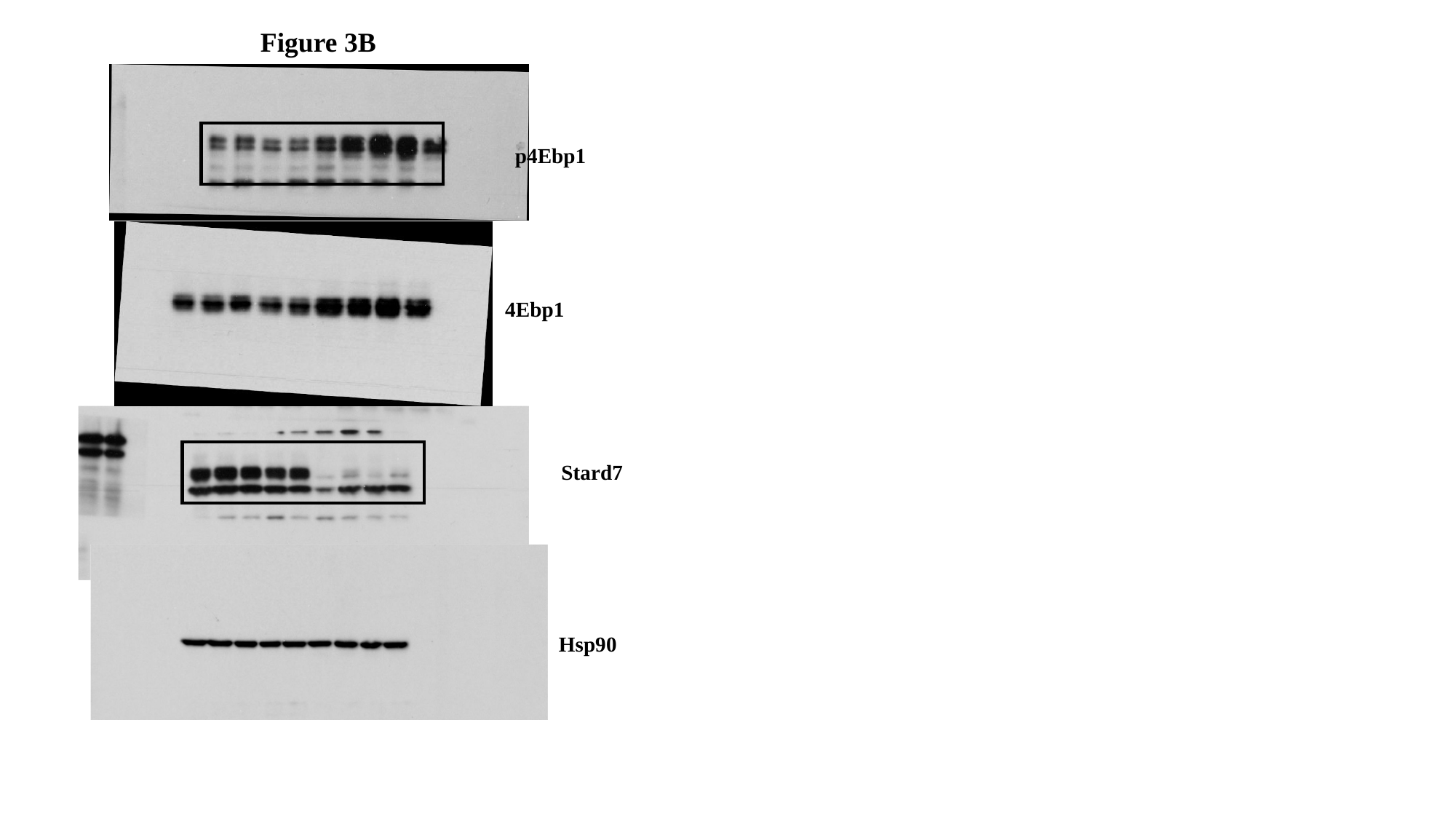

Figure 3B
p4Ebp1
4Ebp1
Stard7
Hsp90

Supplement: Supplementary file 8 — Source data Fig. 3 [file 44321_2026_409_MOESM8_ESM.zip › Fig3/Fig3B/Fig3B.pptx]

## Slide 1
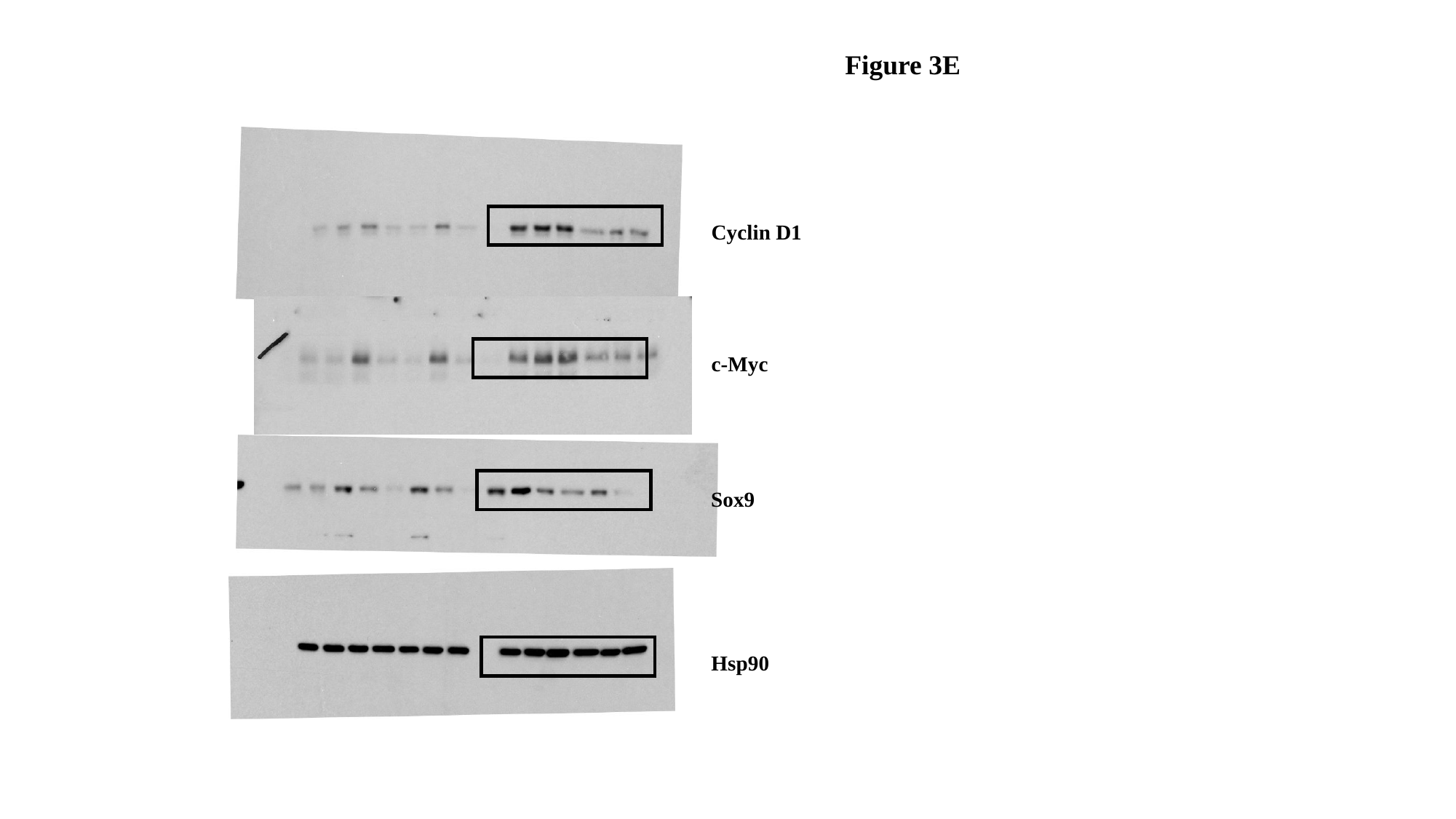

Figure 3E
Cyclin D1
c-Myc
Sox9
Hsp90

Supplement: Supplementary file 8 — Source data Fig. 3 [file 44321_2026_409_MOESM8_ESM.zip › Fig3/Fig3E/Fig3E.pptx]

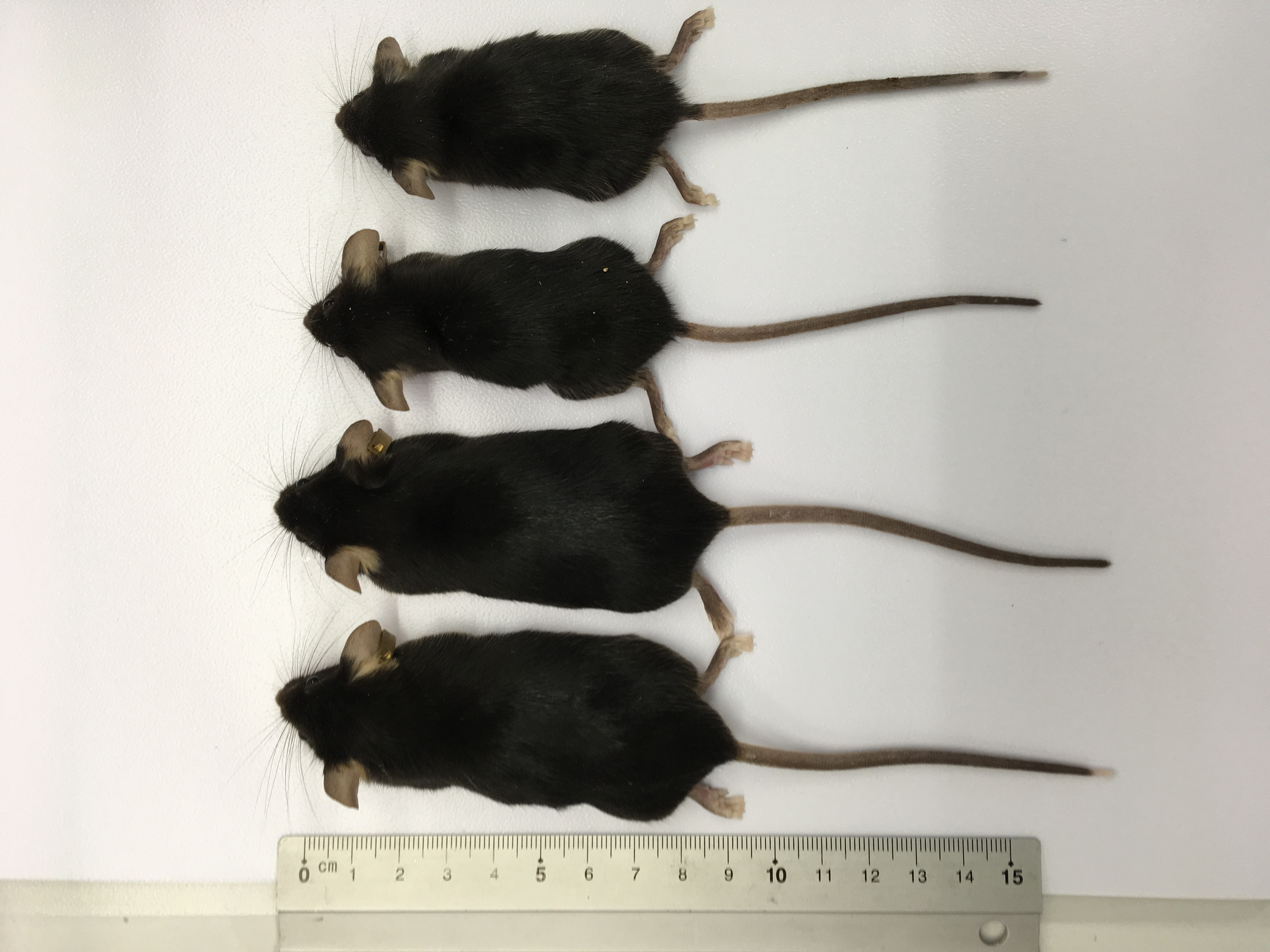

Supplement: Supplementary file 9 — Source data Fig. 4 [file 44321_2026_409_MOESM9_ESM.zip › Fig4/Fig4A/Picture 4 mice Figure 4A.JPG]

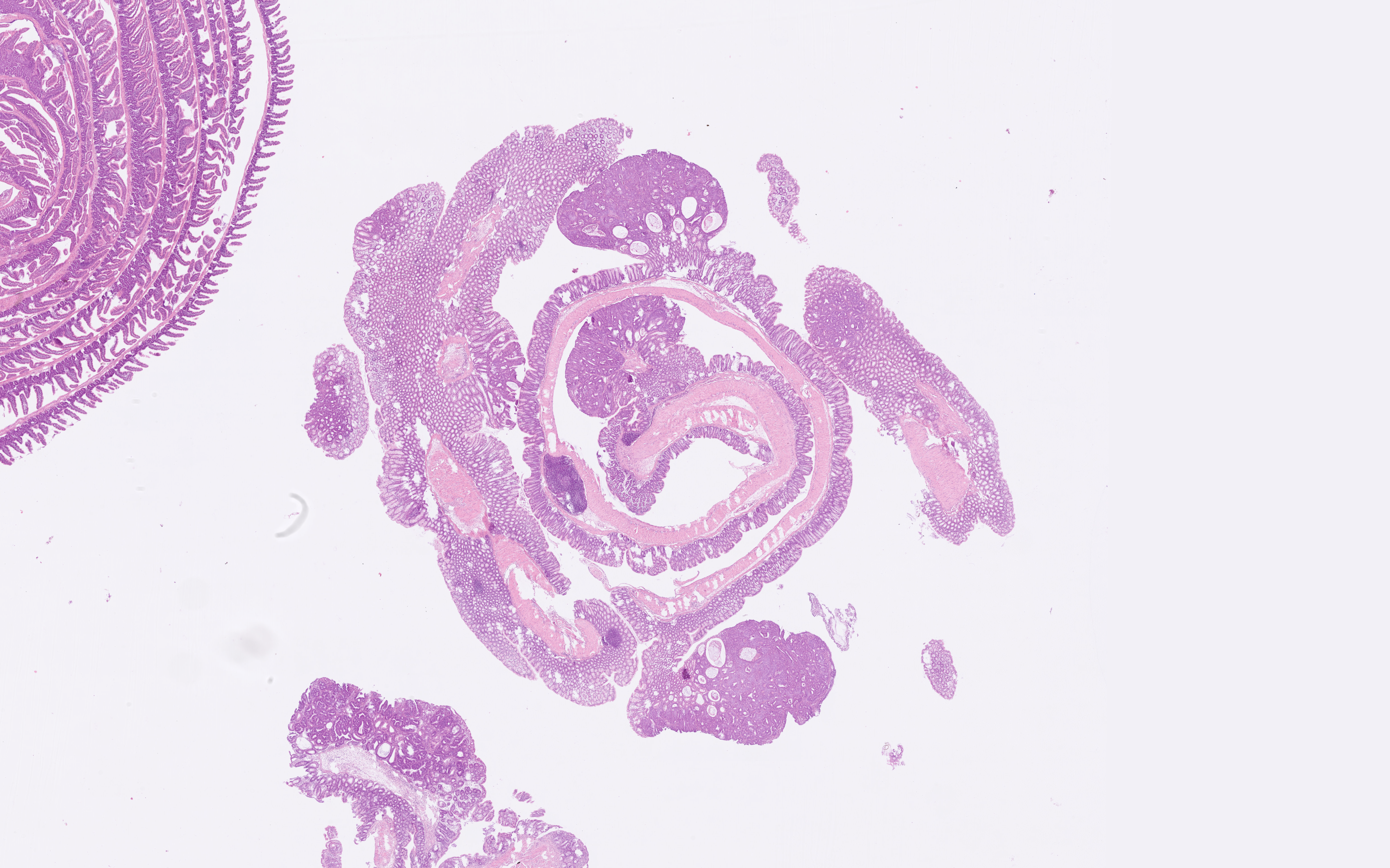

Supplement: Supplementary file 9 — Source data Fig. 4 [file 44321_2026_409_MOESM9_ESM.zip › Fig4/Fig4E/Fig4E IHC/Fig4E KO HE.tif]

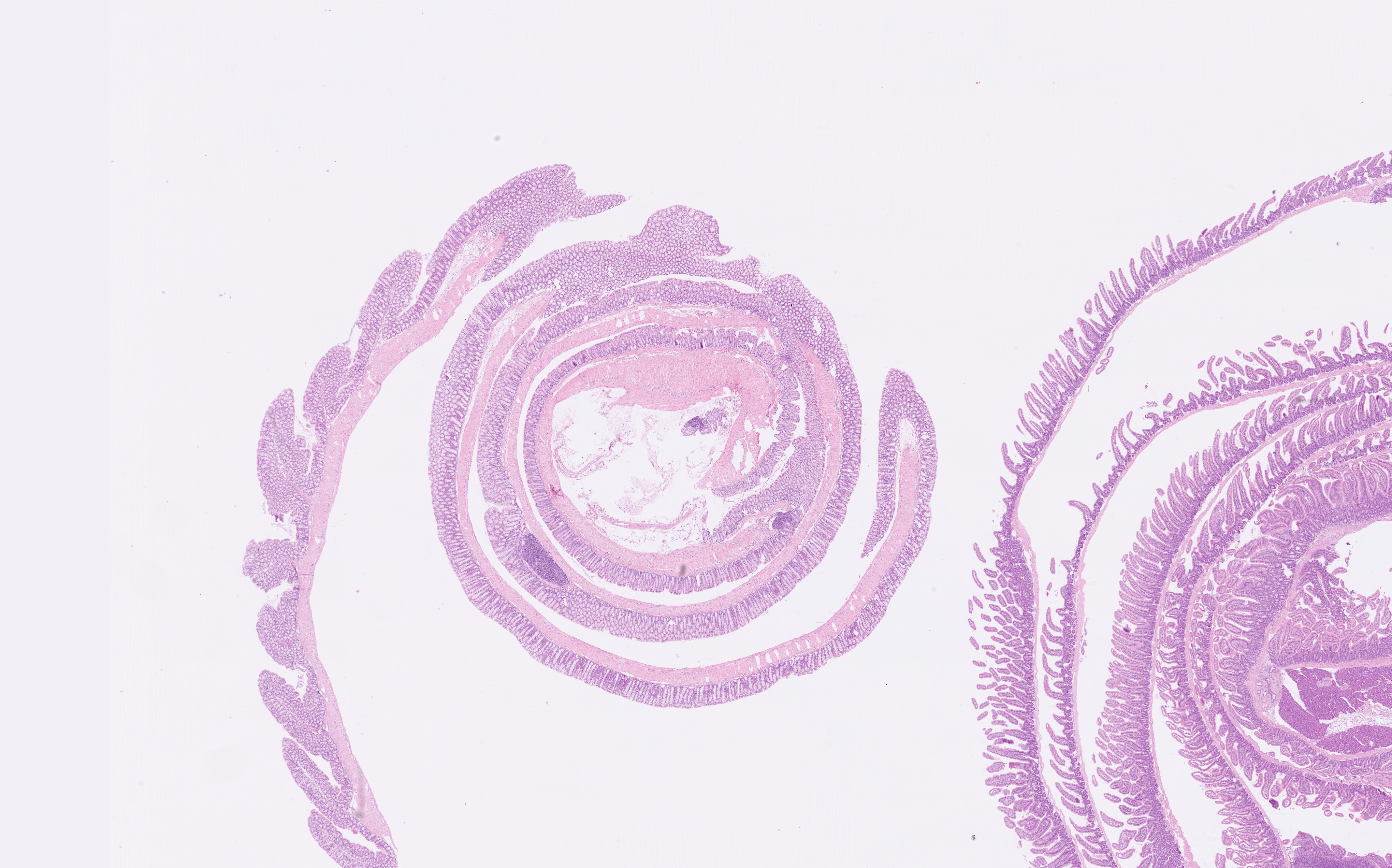

Supplement: Supplementary file 9 — Source data Fig. 4 [file 44321_2026_409_MOESM9_ESM.zip › Fig4/Fig4E/Fig4E IHC/Fig4E WT HE.tif]

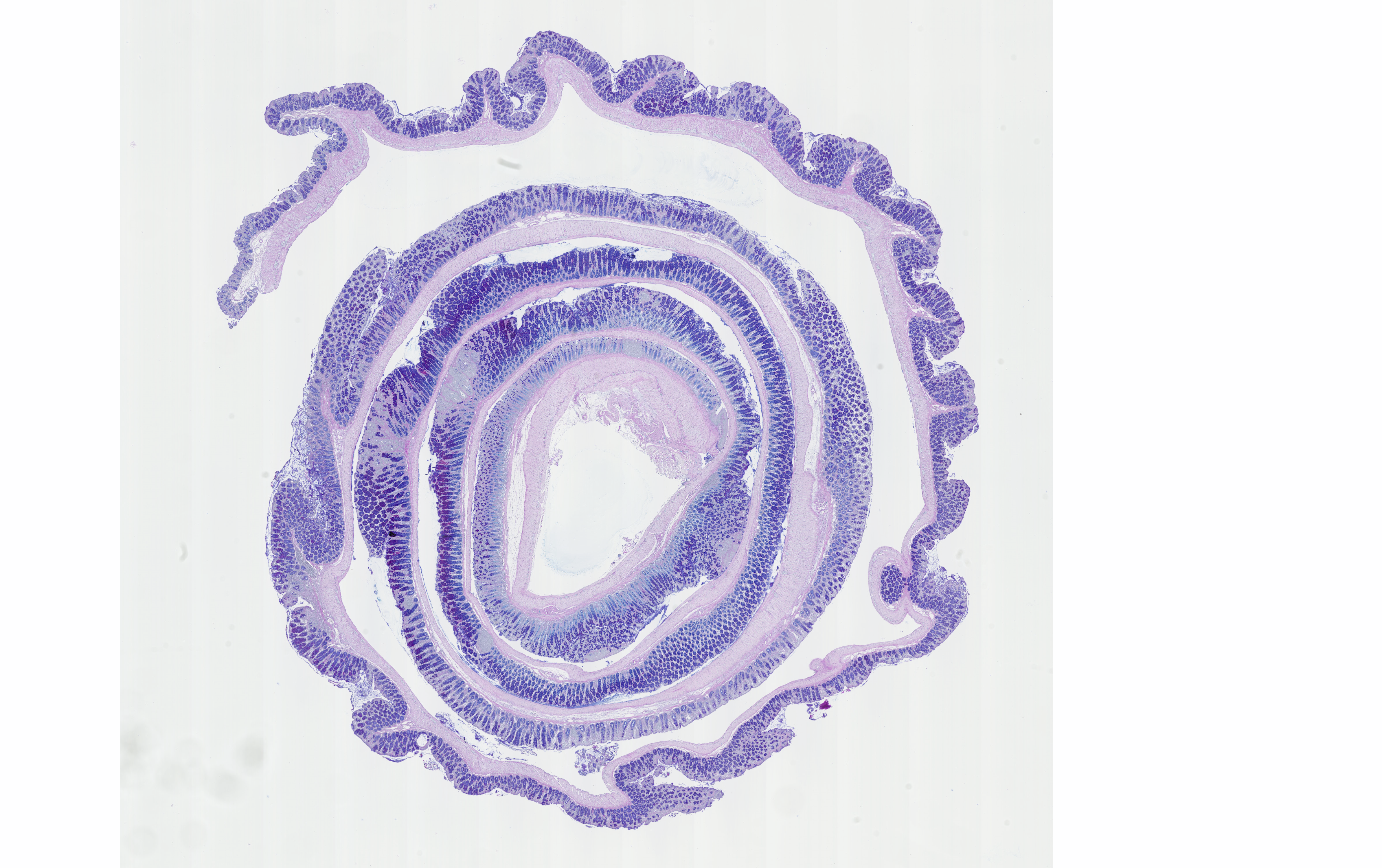

Supplement: Supplementary file 9 — Source data Fig. 4 [file 44321_2026_409_MOESM9_ESM.zip › Fig4/Fig4F/Fig4F IHC/Fig 4F WT.tif]

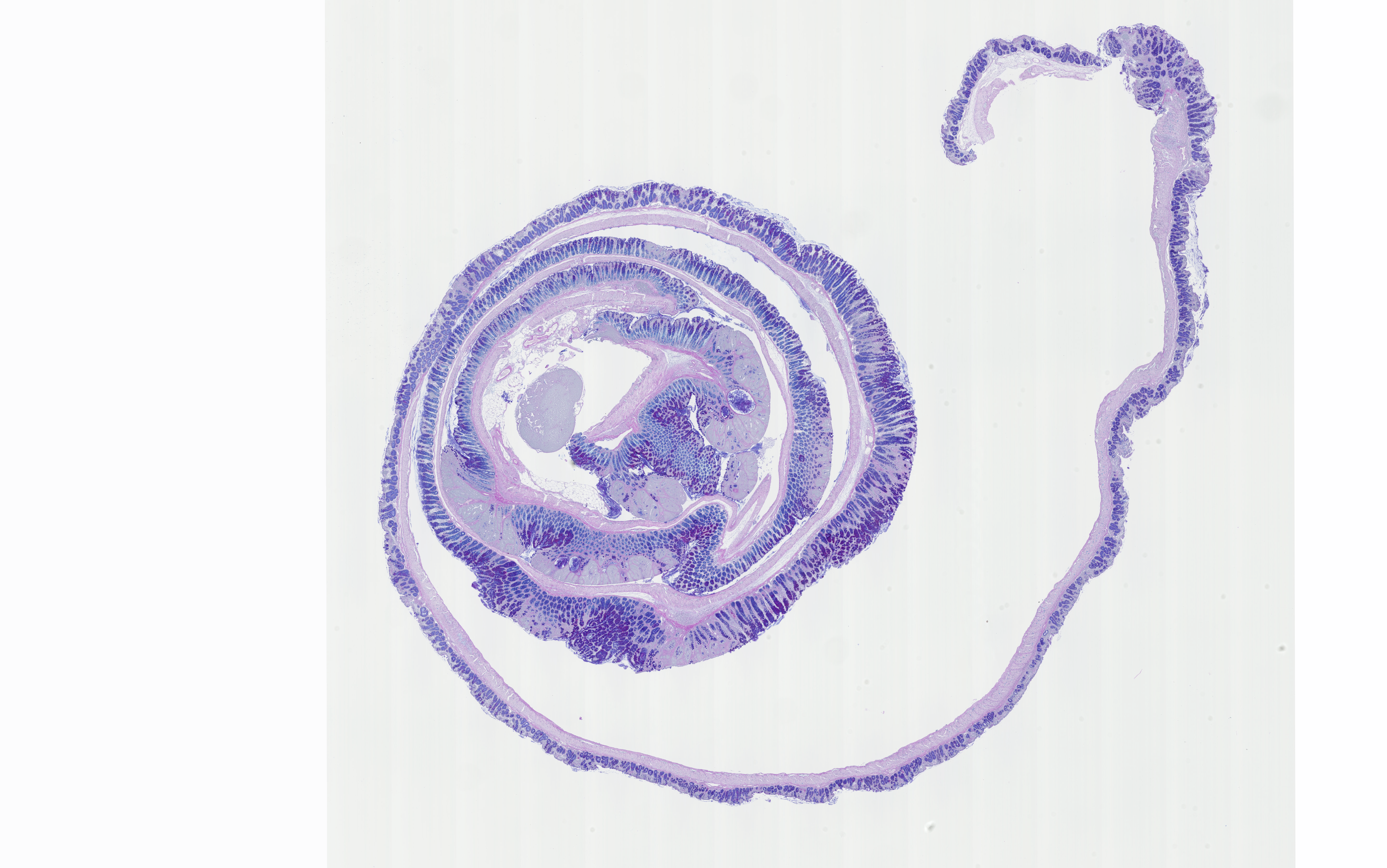

Supplement: Supplementary file 9 — Source data Fig. 4 [file 44321_2026_409_MOESM9_ESM.zip › Fig4/Fig4F/Fig4F IHC/Fig4F KO.tif]

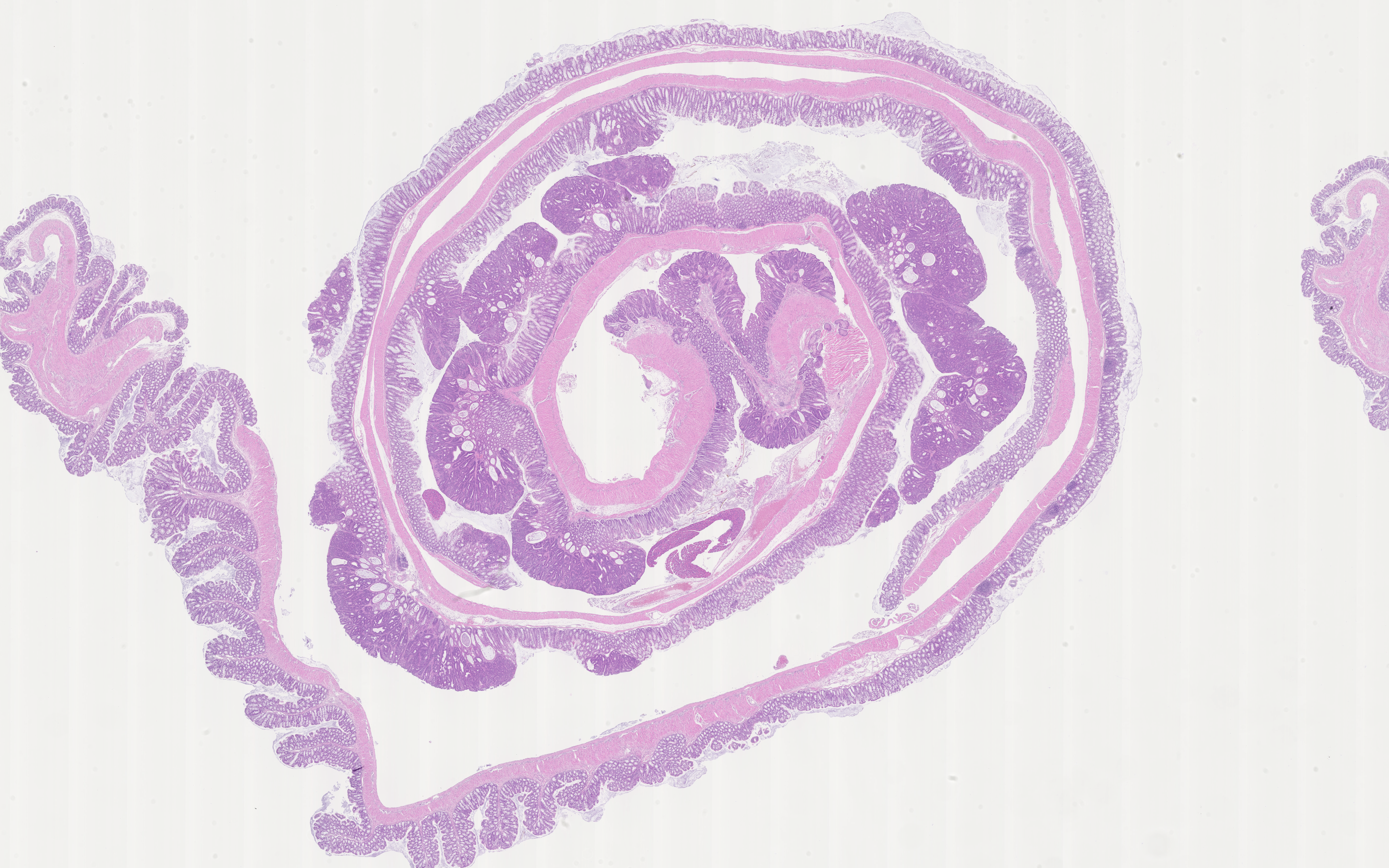

Supplement: Supplementary file 9 — Source data Fig. 4 [file 44321_2026_409_MOESM9_ESM.zip › Fig4/Fig4G/Fig 4G IHC/Fig 4G KO.tif]

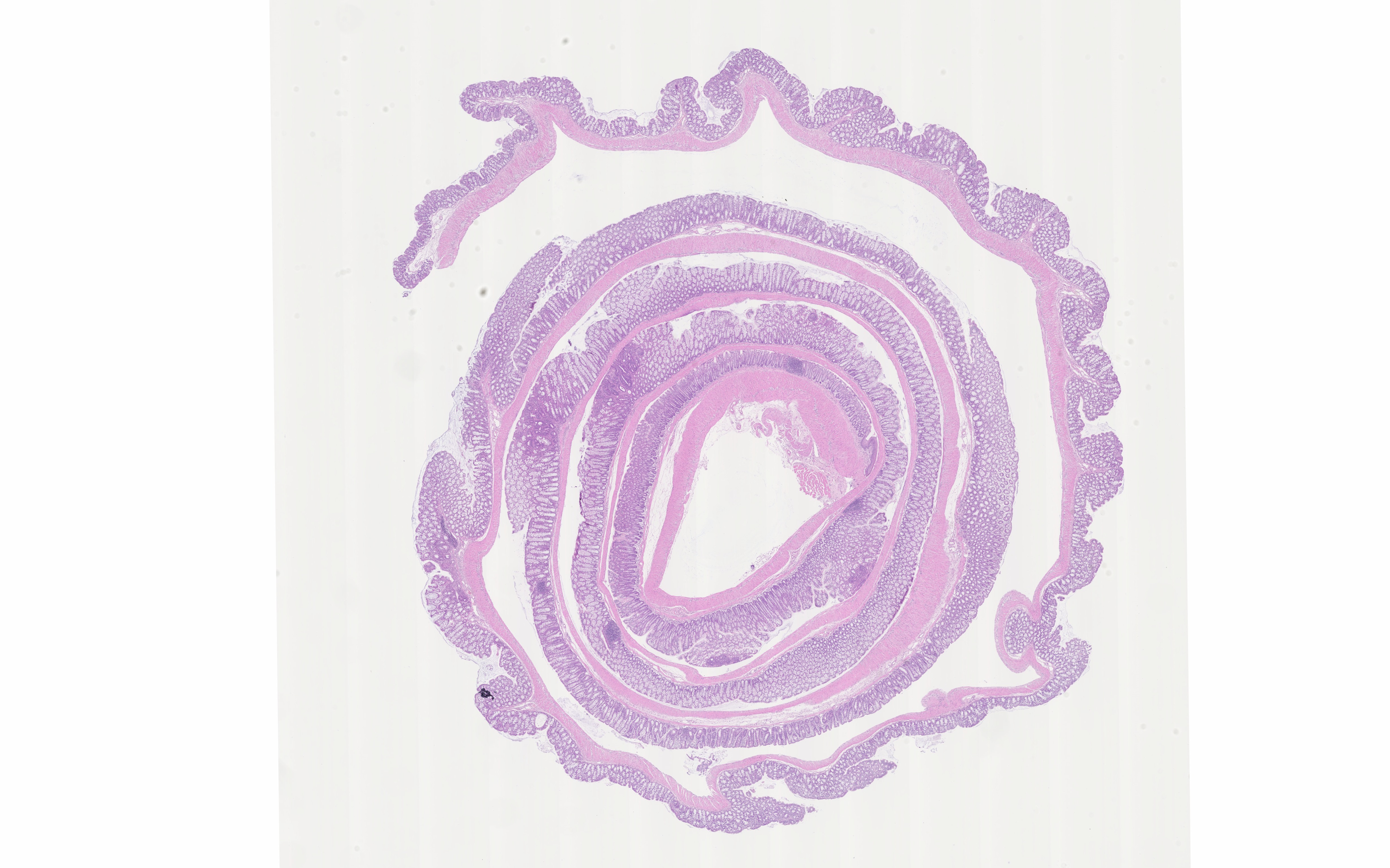

Supplement: Supplementary file 9 — Source data Fig. 4 [file 44321_2026_409_MOESM9_ESM.zip › Fig4/Fig4G/Fig 4G IHC/Fig4G WT.tif]

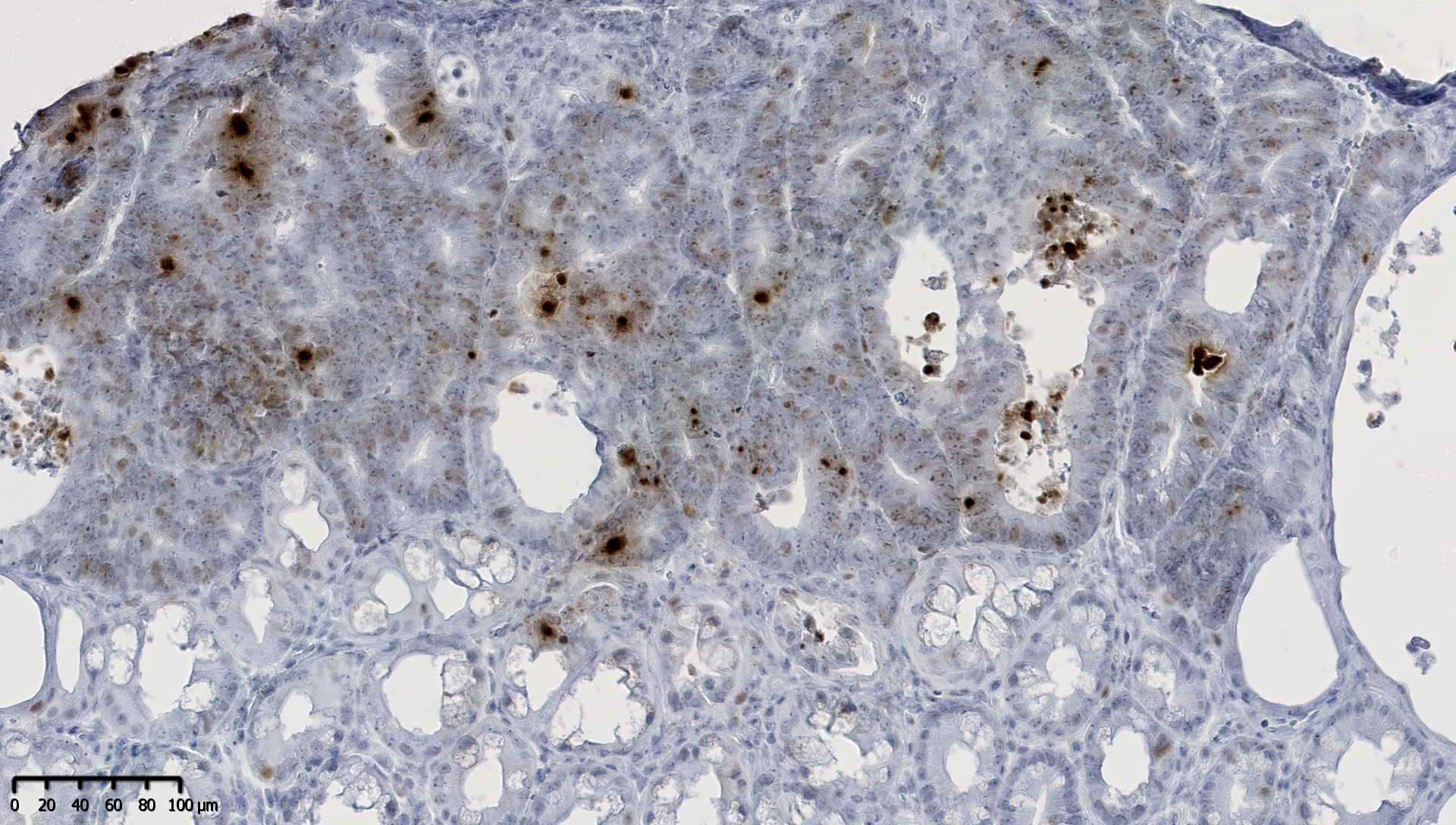

Supplement: Supplementary file 10 — Source data Fig. 5 [file 44321_2026_409_MOESM10_ESM.zip › Fig5/Fig5G/Fig 5G IHC/Figure 5G KO.tif]

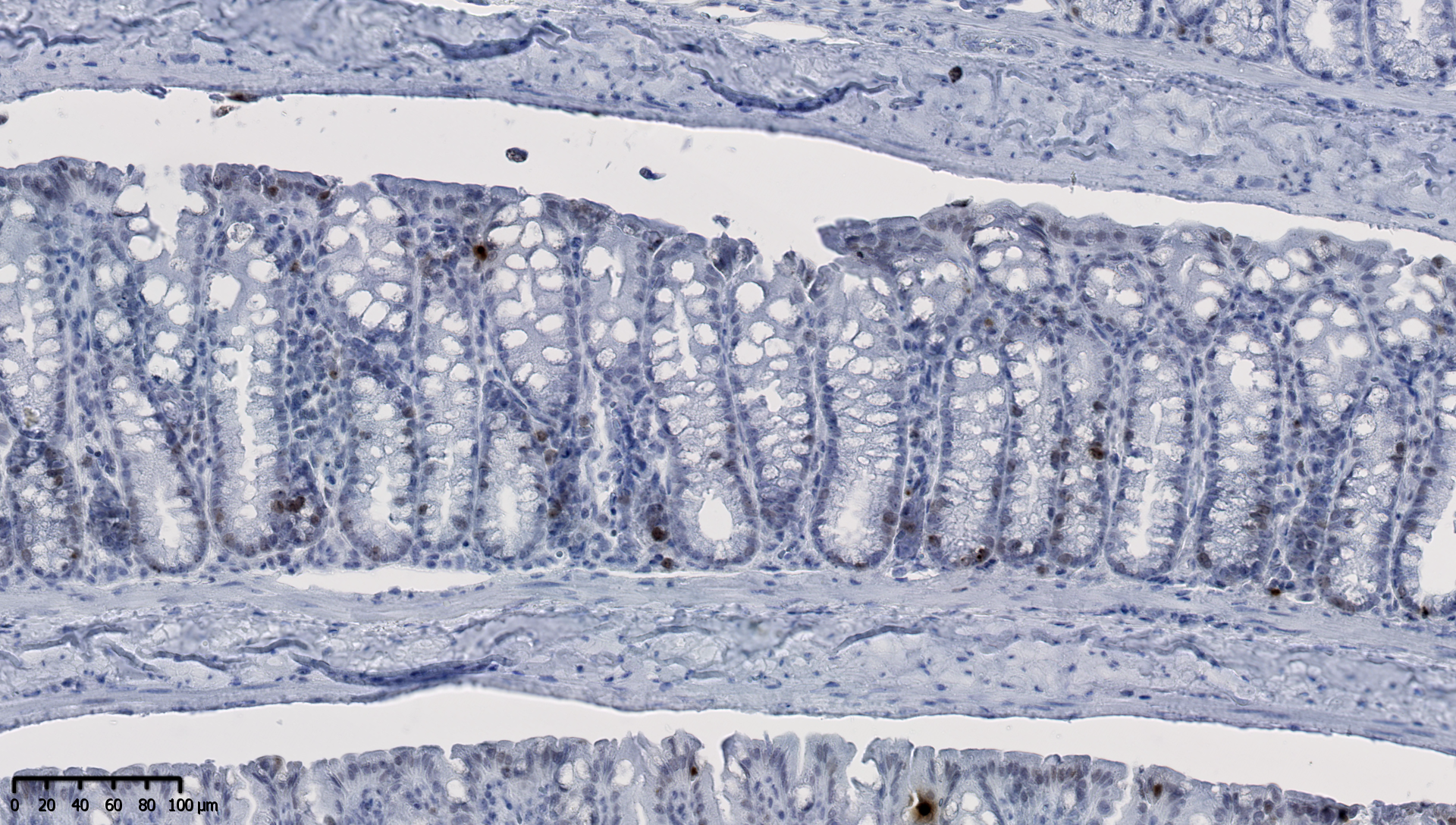

Supplement: Supplementary file 10 — Source data Fig. 5 [file 44321_2026_409_MOESM10_ESM.zip › Fig5/Fig5G/Fig 5G IHC/Figure 5G WT.tif]

## Slide 1
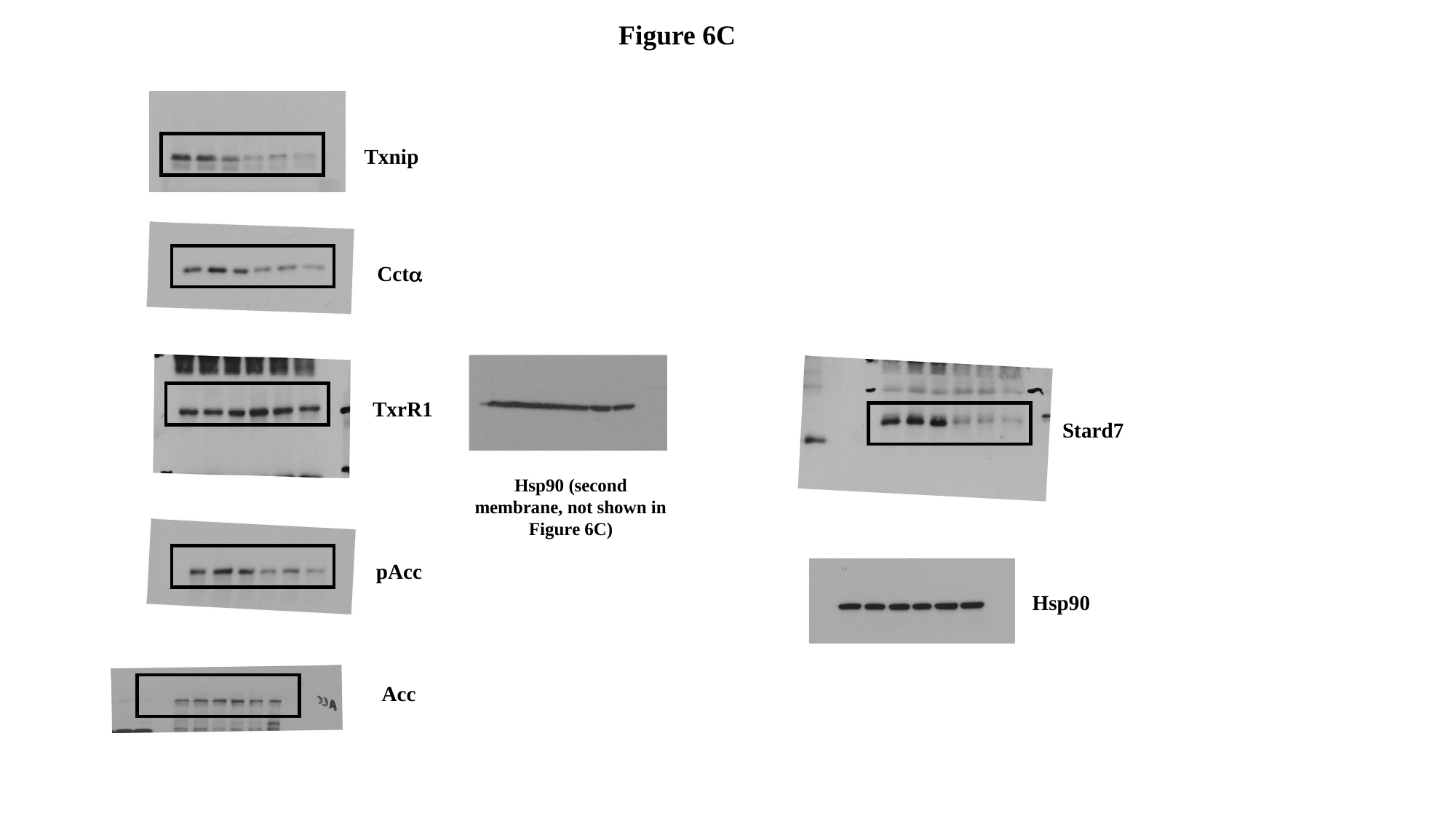

Figure 6C
Txnip
Ccta
TxrR1
Stard7
Hsp90 (second membrane, not shown in Figure 6C)
pAcc
Hsp90
Acc

Supplement: Supplementary file 11 — Source data Fig. 6 [file 44321_2026_409_MOESM11_ESM.zip › Fig6/Fig6C/Fig6C.pptx]

## Slide 1
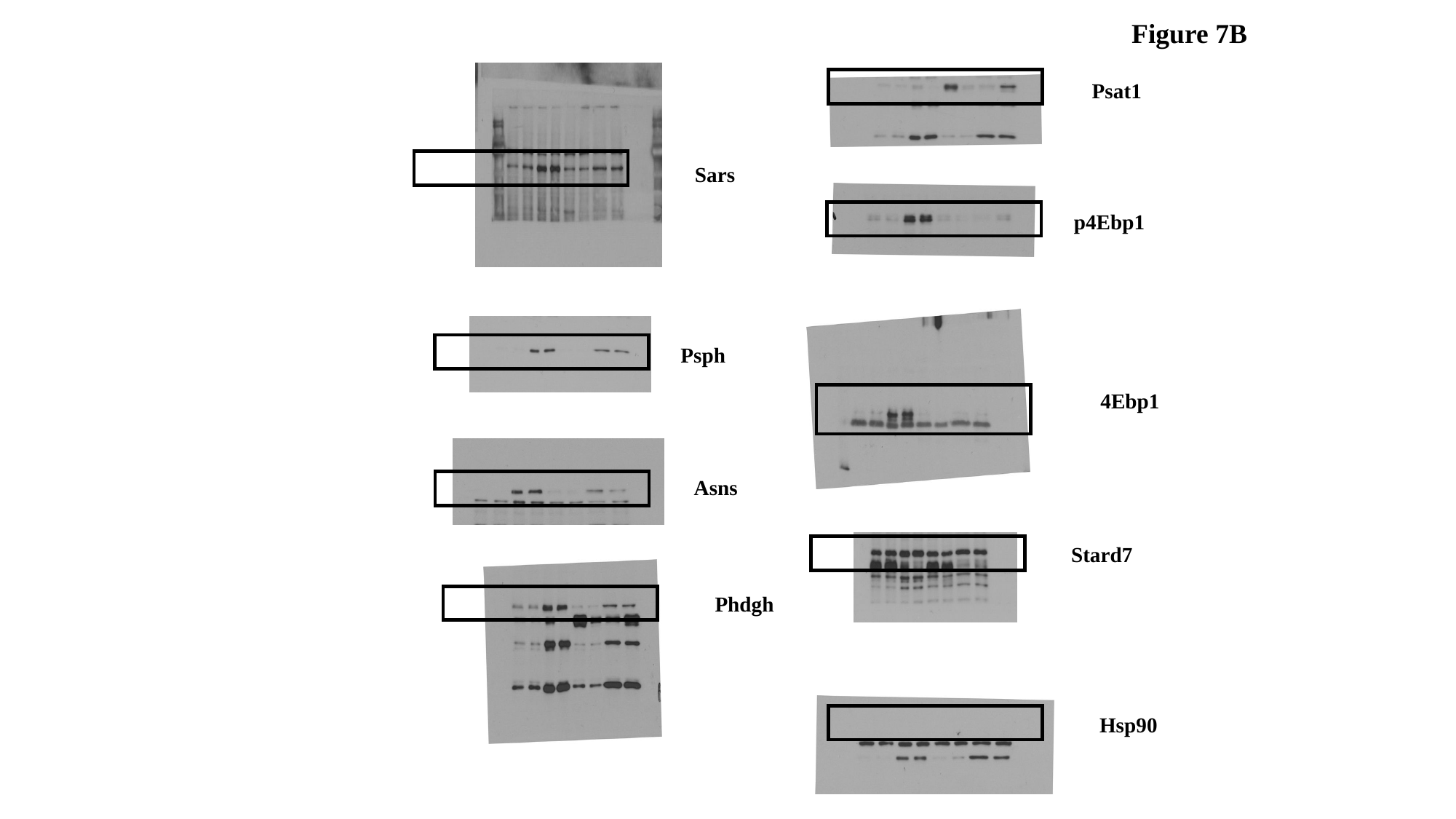

Figure 7B
Psat1
Sars
p4Ebp1
Psph
4Ebp1
Asns
Stard7
Phdgh
Hsp90

Supplement: Supplementary file 12 — Source data Fig. 7 [file 44321_2026_409_MOESM12_ESM.zip › Fig7/Fig7B/Fig7B.pptx]

## Slide 1
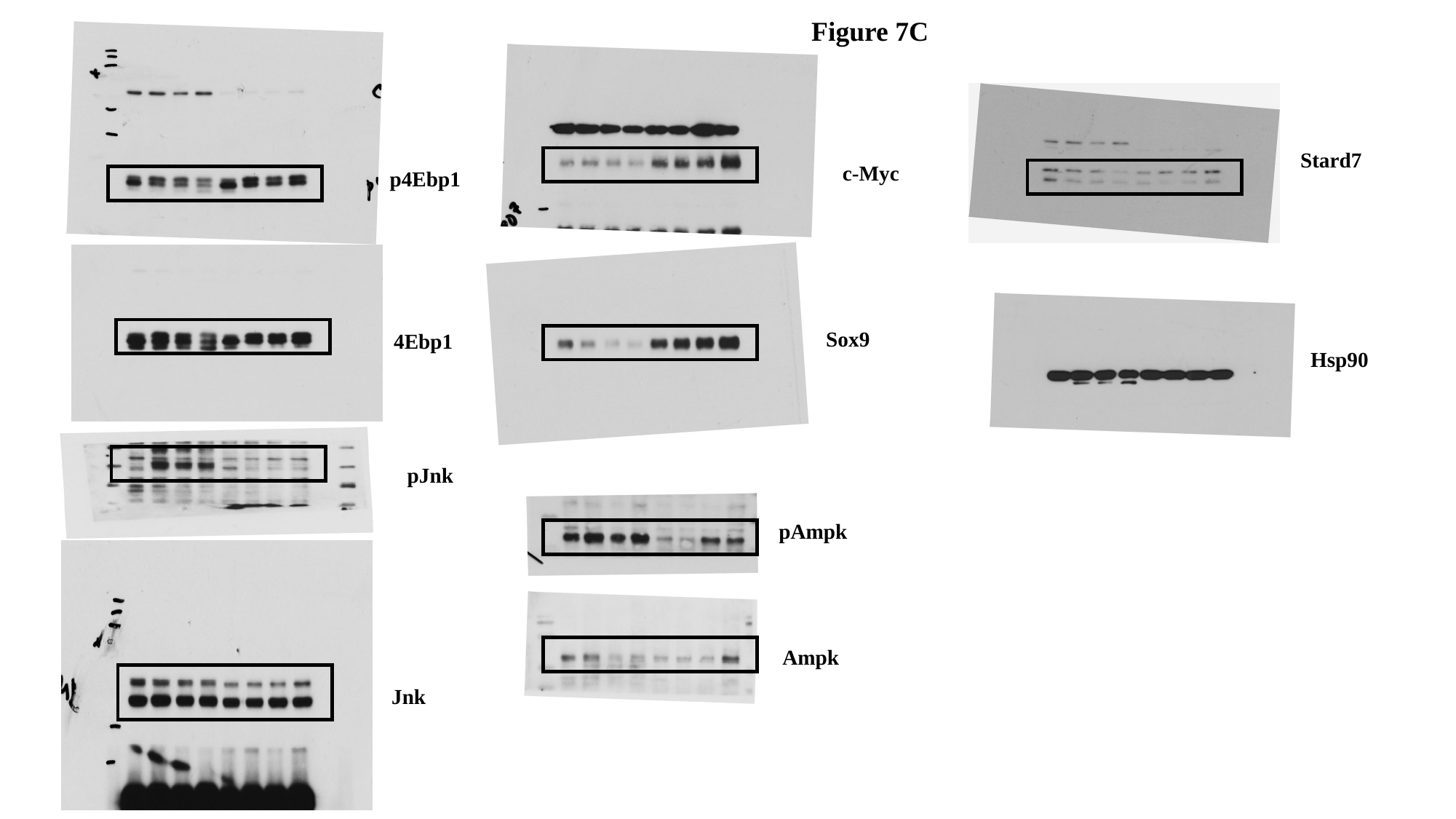

Figure 7C
Stard7
c-Myc
p4Ebp1
Sox9
4Ebp1
Hsp90
pJnk
pAmpk
Ampk
Jnk

Supplement: Supplementary file 12 — Source data Fig. 7 [file 44321_2026_409_MOESM12_ESM.zip › Fig7/Fig7C/Fig7C.pptx]

## Slide 1
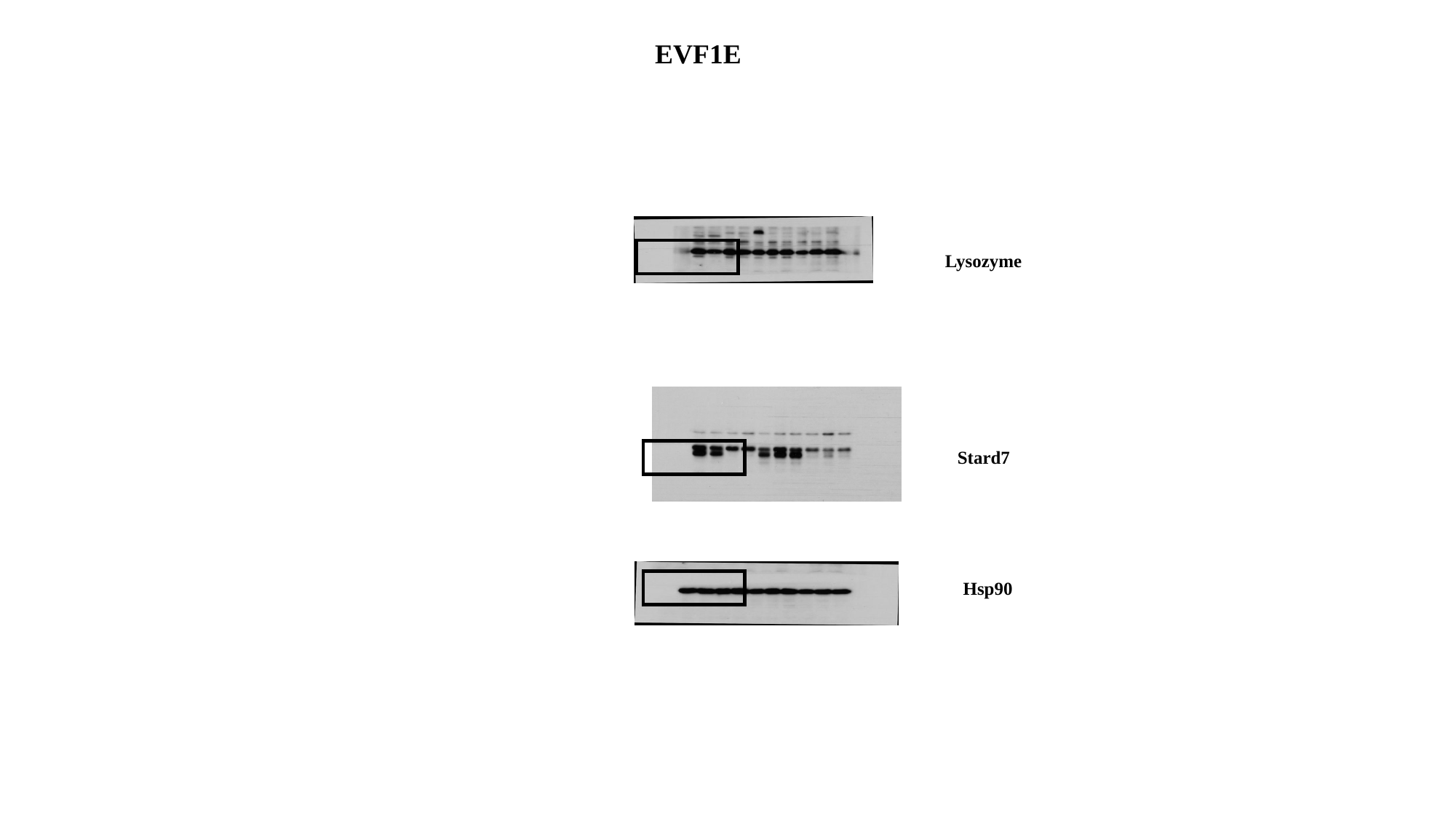

EVF1E
Lysozyme
Stard7
Hsp90

Supplement: Supplementary file 14 — Figure EV1 Source Data [file 44321_2026_409_MOESM14_ESM.zip › EVF1/EVF1E/EVF1E.pptx]

## Slide 1
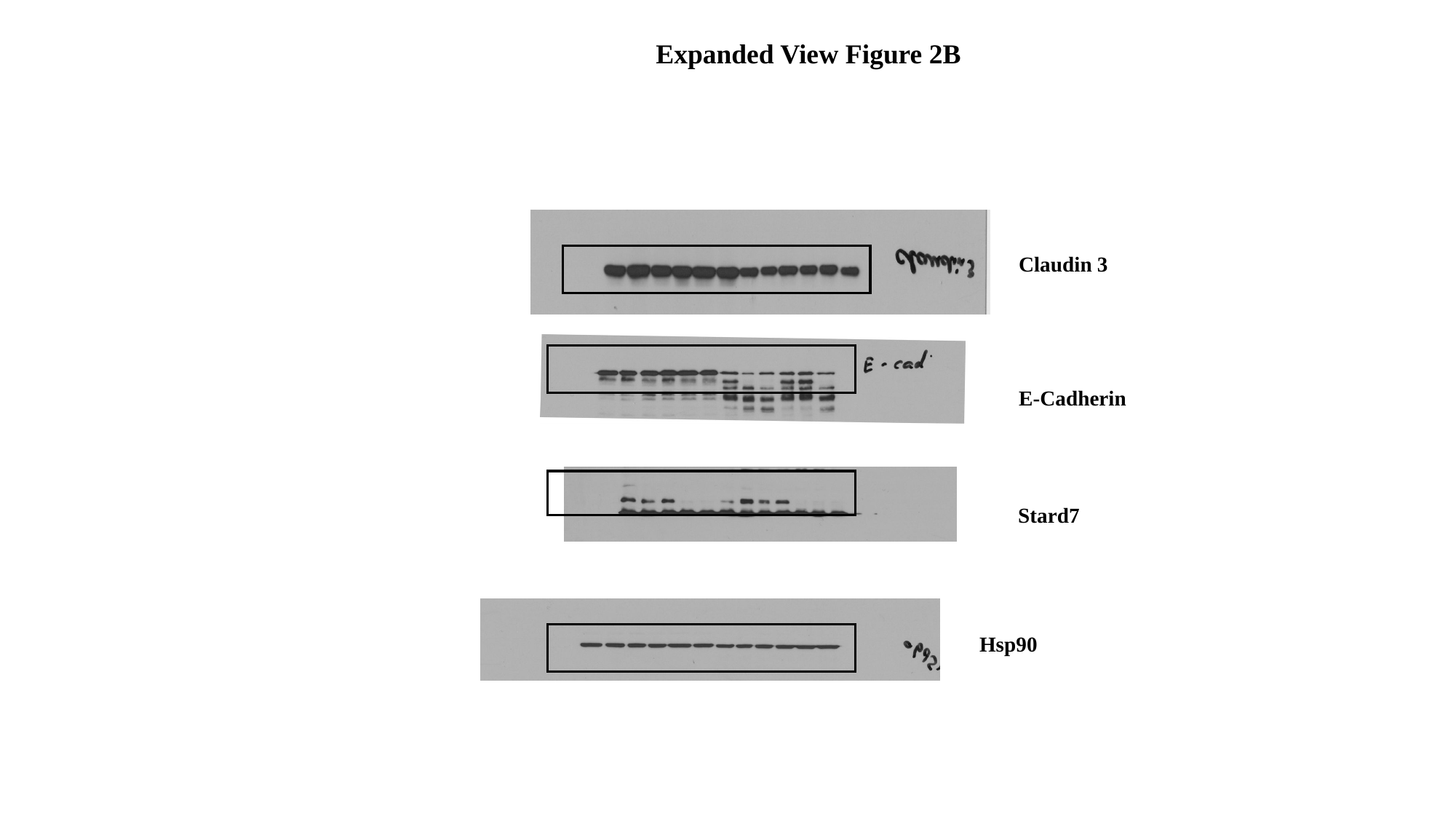

Expanded View Figure 2B
Claudin 3
E-Cadherin
Stard7
Hsp90

Supplement: Supplementary file 15 — Figure EV2 Source Data [file 44321_2026_409_MOESM15_ESM.zip › EVF2/EVF2B/EVF2B.pptx]

## Slide 1
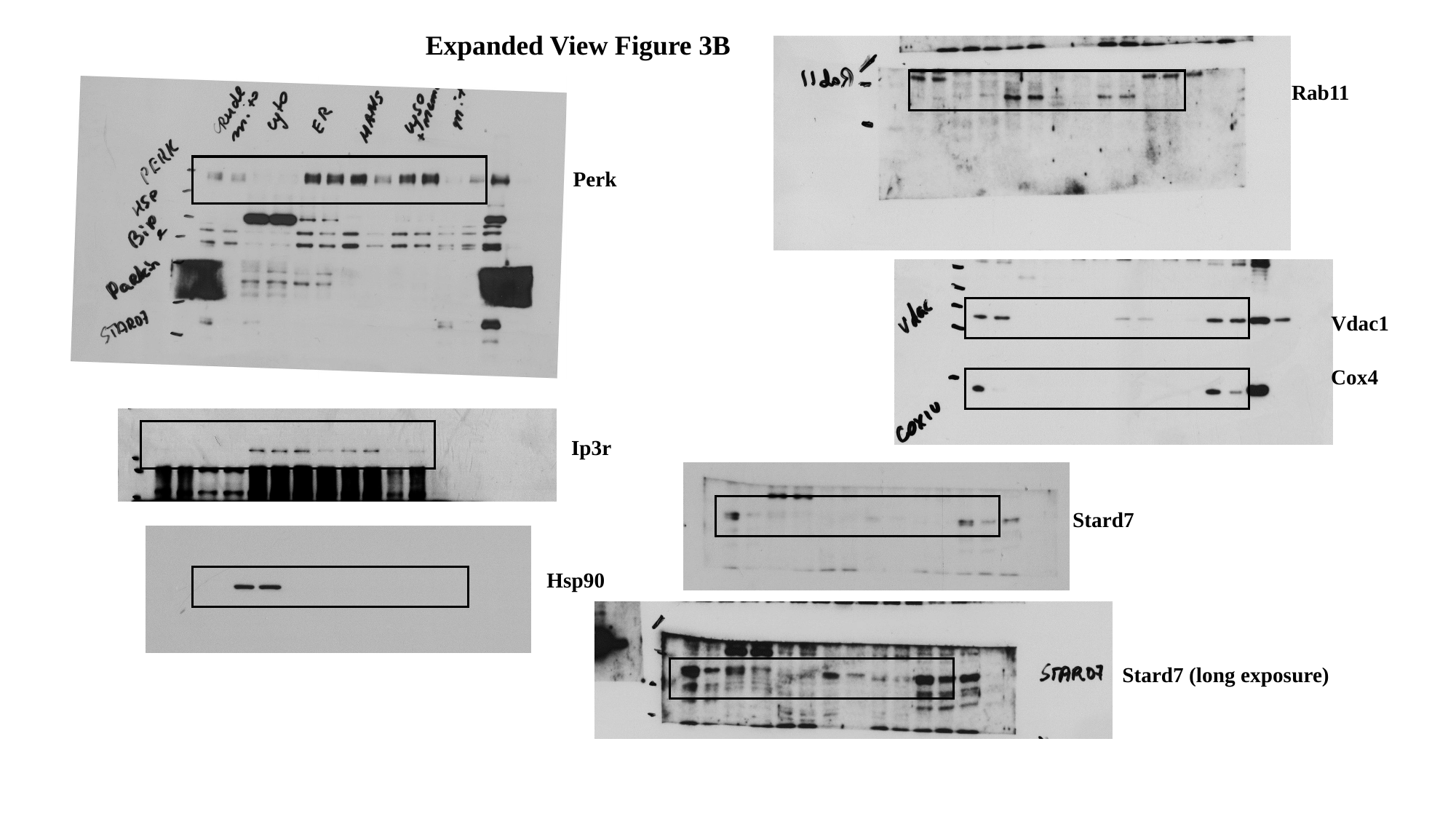

Expanded View Figure 3B
Rab11
Perk
Vdac1
Cox4
Ip3r
Stard7
Hsp90
Stard7 (long exposure)

Supplement: Supplementary file 16 — Figure EV3 Source Data [file 44321_2026_409_MOESM16_ESM.zip › EVF3/EVF3B/EVF3B.pptx]

## Slide 1
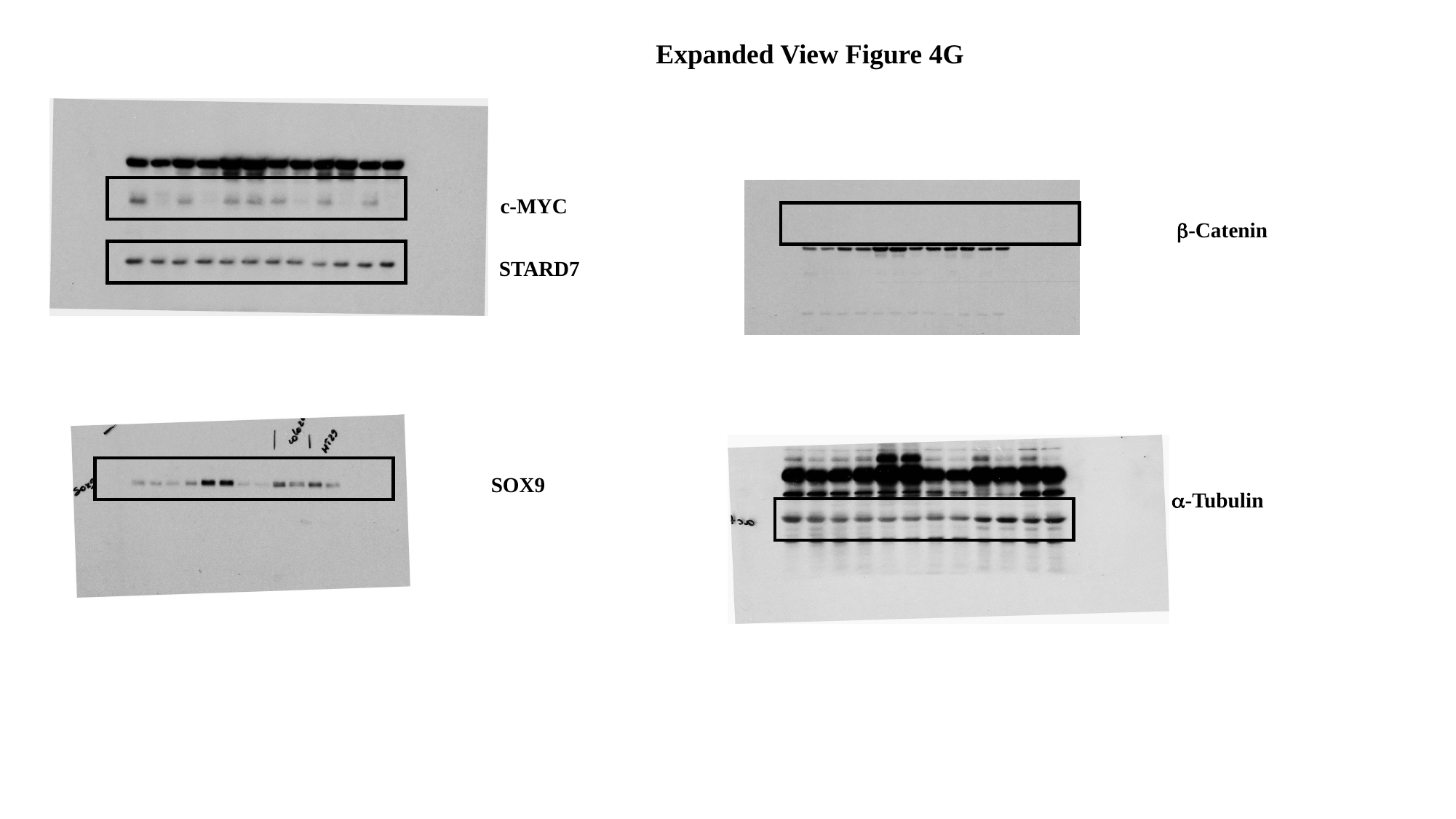

Expanded View Figure 4G
c-MYC
b-Catenin
STARD7
SOX9
a-Tubulin

Supplement: Supplementary file 17 — Figure EV4 Source Data [file 44321_2026_409_MOESM17_ESM.zip › EVF4/EVF4G/EVF4G.pptx]

## Slide 1
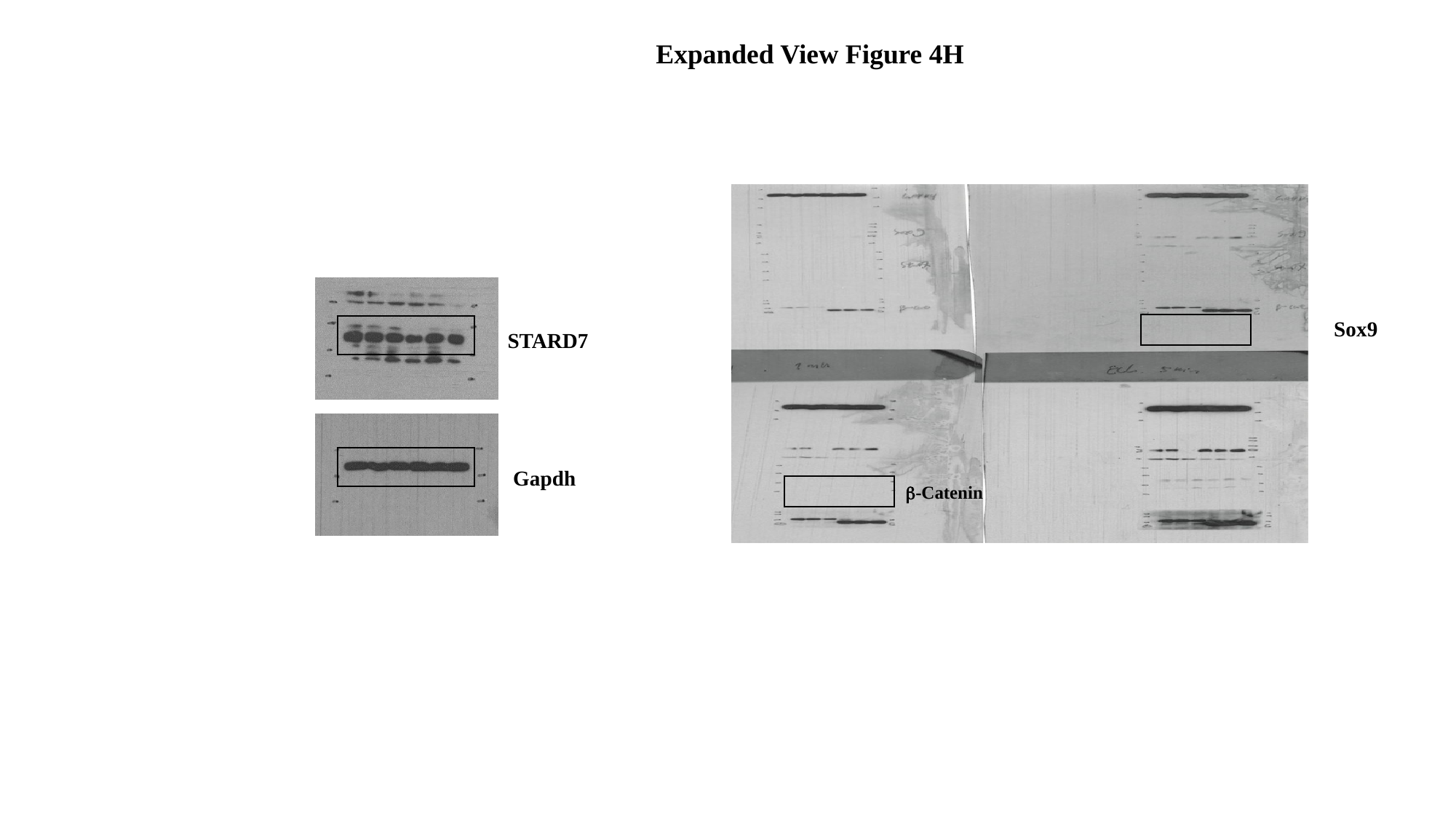

Expanded View Figure 4H
Sox9
STARD7
Gapdh
b-Catenin

Supplement: Supplementary file 17 — Figure EV4 Source Data [file 44321_2026_409_MOESM17_ESM.zip › EVF4/EVF4H/EVF4H.pptx]

## Slide 1
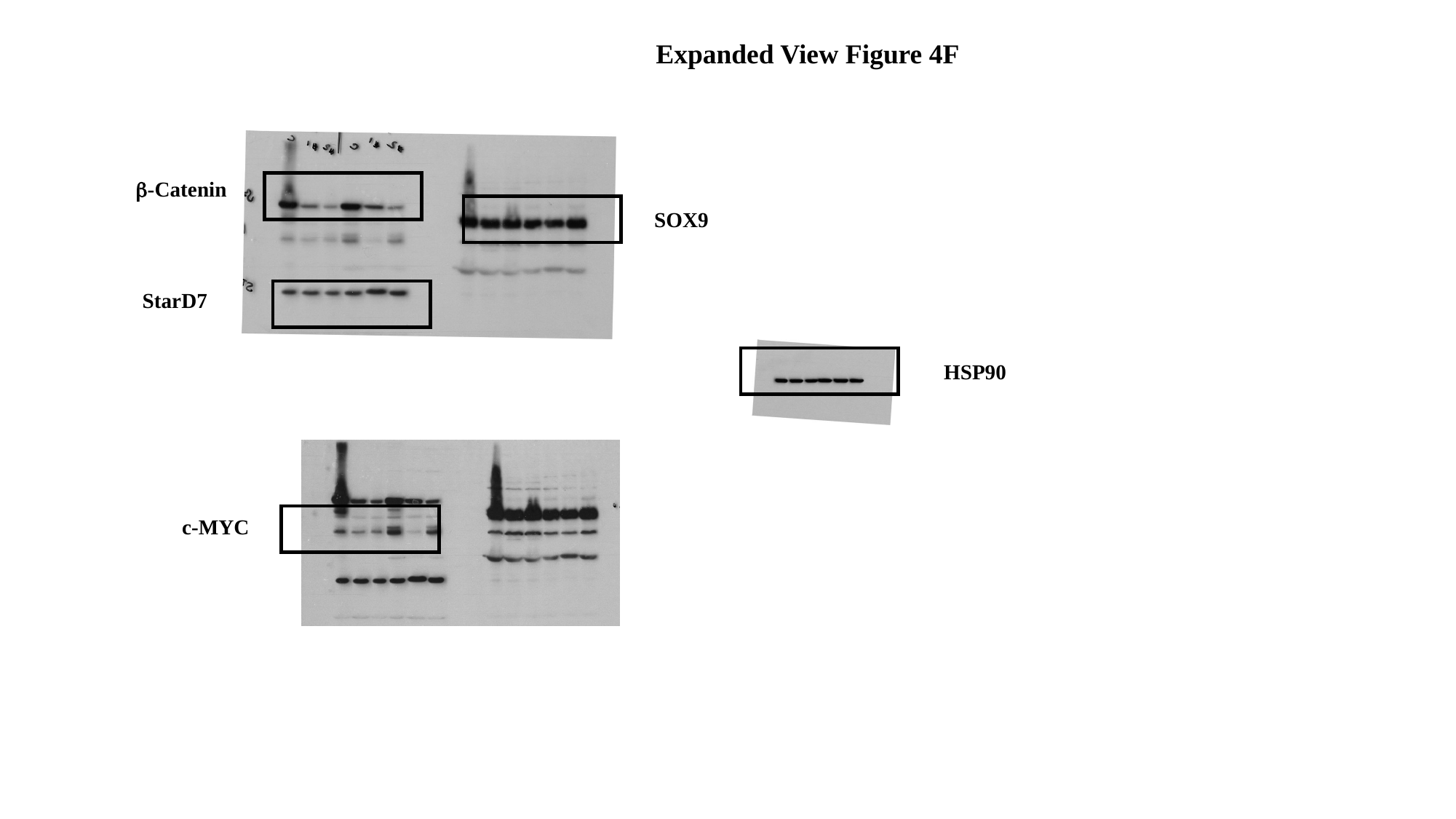

Expanded View Figure 4F
b-Catenin
SOX9
StarD7
HSP90
c-MYC

Supplement: Supplementary file 17 — Figure EV4 Source Data [file 44321_2026_409_MOESM17_ESM.zip › EVF4/EVF4F/EVF4F.pptx]

## Slide 1
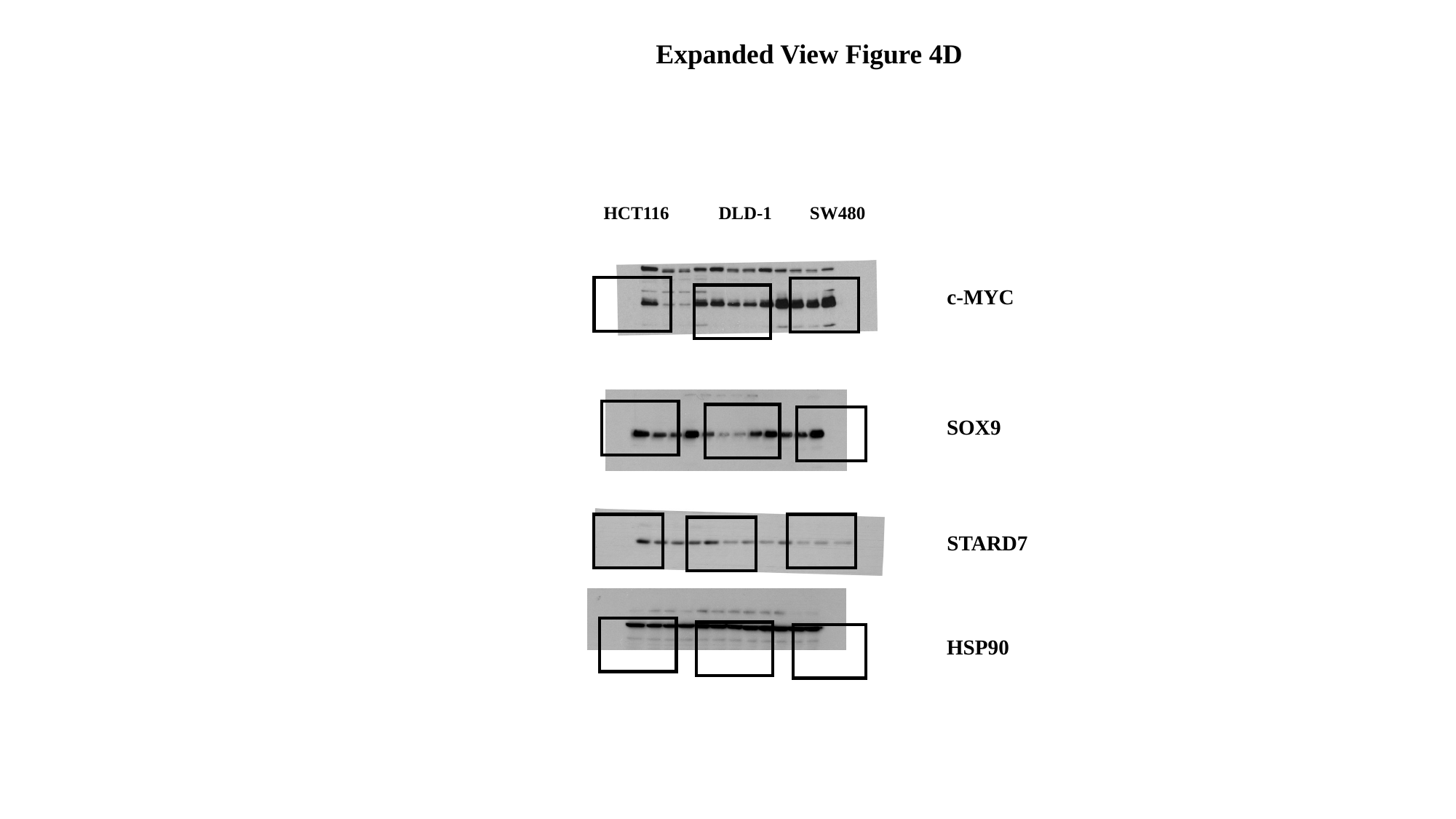

Expanded View Figure 4D
HCT116
DLD-1
SW480
c-MYC
SOX9
STARD7
HSP90

Supplement: Supplementary file 17 — Figure EV4 Source Data [file 44321_2026_409_MOESM17_ESM.zip › EVF4/EVF4D/EVF4D.pptx]

## Slide 1
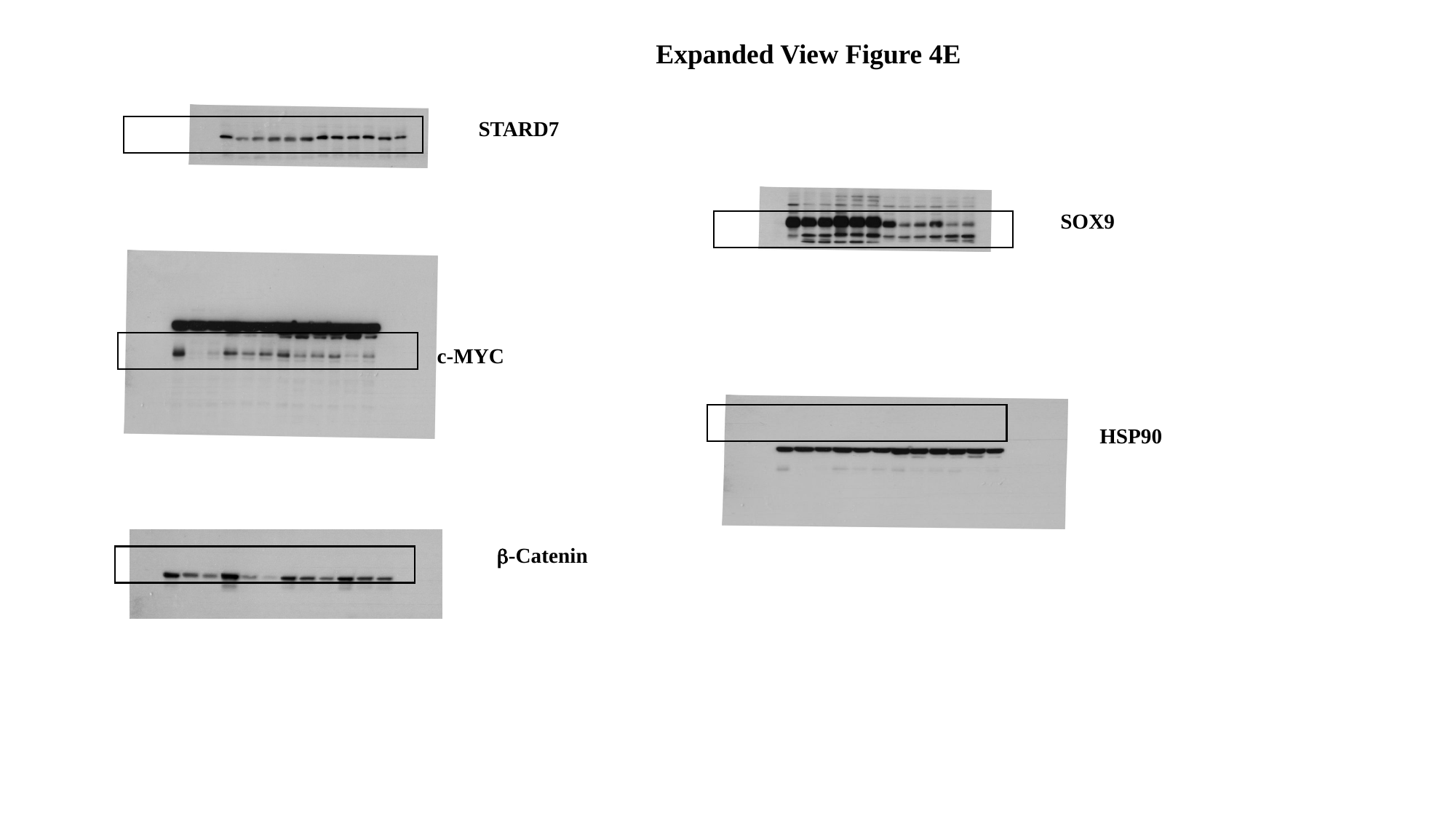

Expanded View Figure 4E
STARD7
SOX9
c-MYC
HSP90
b-Catenin

Supplement: Supplementary file 17 — Figure EV4 Source Data [file 44321_2026_409_MOESM17_ESM.zip › EVF4/EVF4E/EVF4E.pptx]

## Slide 1
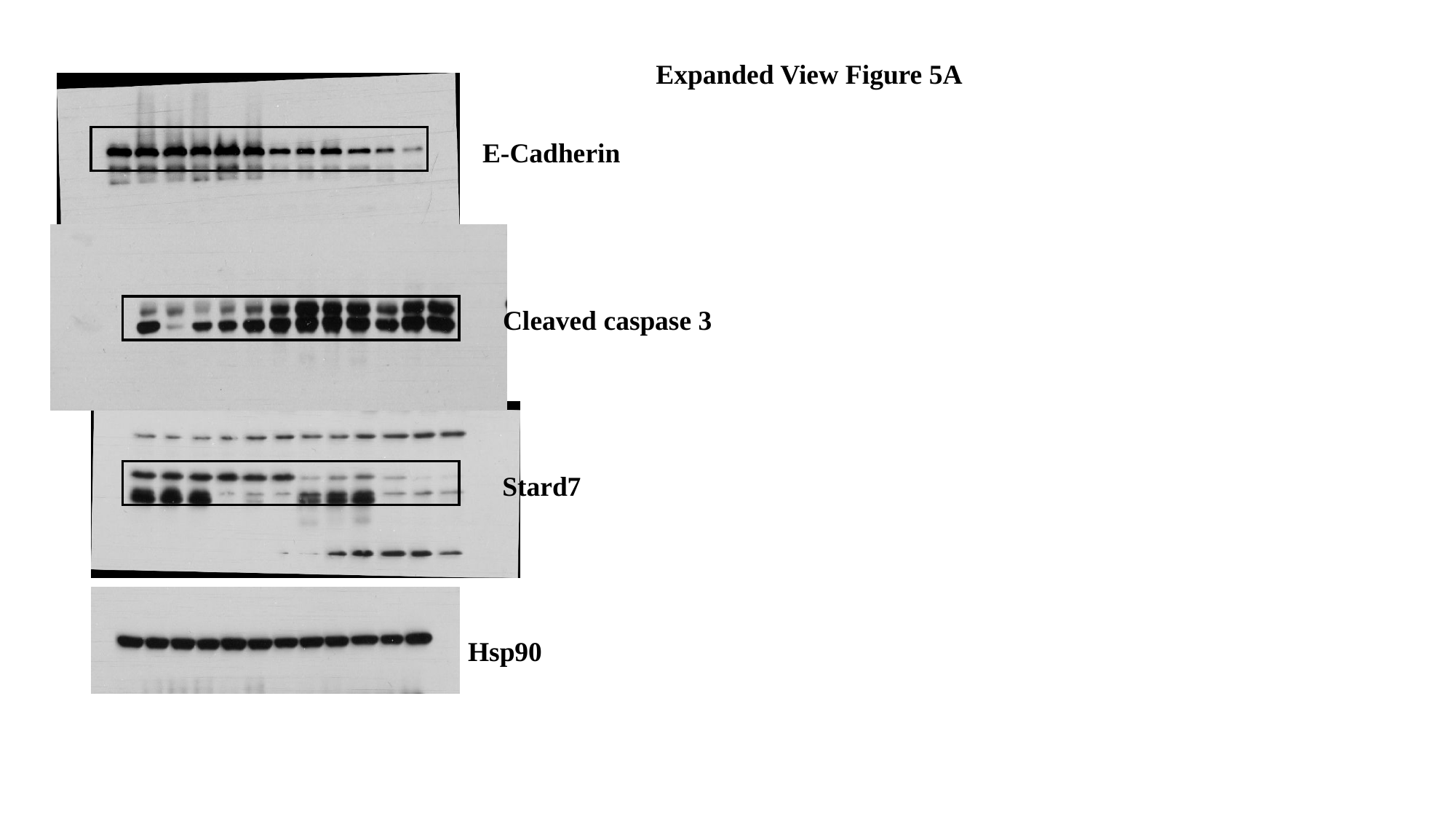

Expanded View Figure 5A
E-Cadherin
Cleaved caspase 3
Stard7
Hsp90

Supplement: Supplementary file 18 — Figure EV5 Source Data [file 44321_2026_409_MOESM18_ESM.zip › EVF5/EVF5A/EVF5A.pptx]
