# Supplementary material for: The lipid transfer protein STARD7 controls intestinal tumor development in a context-dependent manner
Source: EMBO Mol Med. 2026 Mar 30;18(5):1771–811. doi: 10.1038/s44321-026-00409-5 (PMC13179355; doi:10.1038/s44321-026-00409-5)
Supplement: Supplementary file 8 — Source data Fig. 3 [file 44321_2026_409_MOESM8_ESM.zip › Fig3/Fig3C/Fig3C.pptx]

## Slide 1
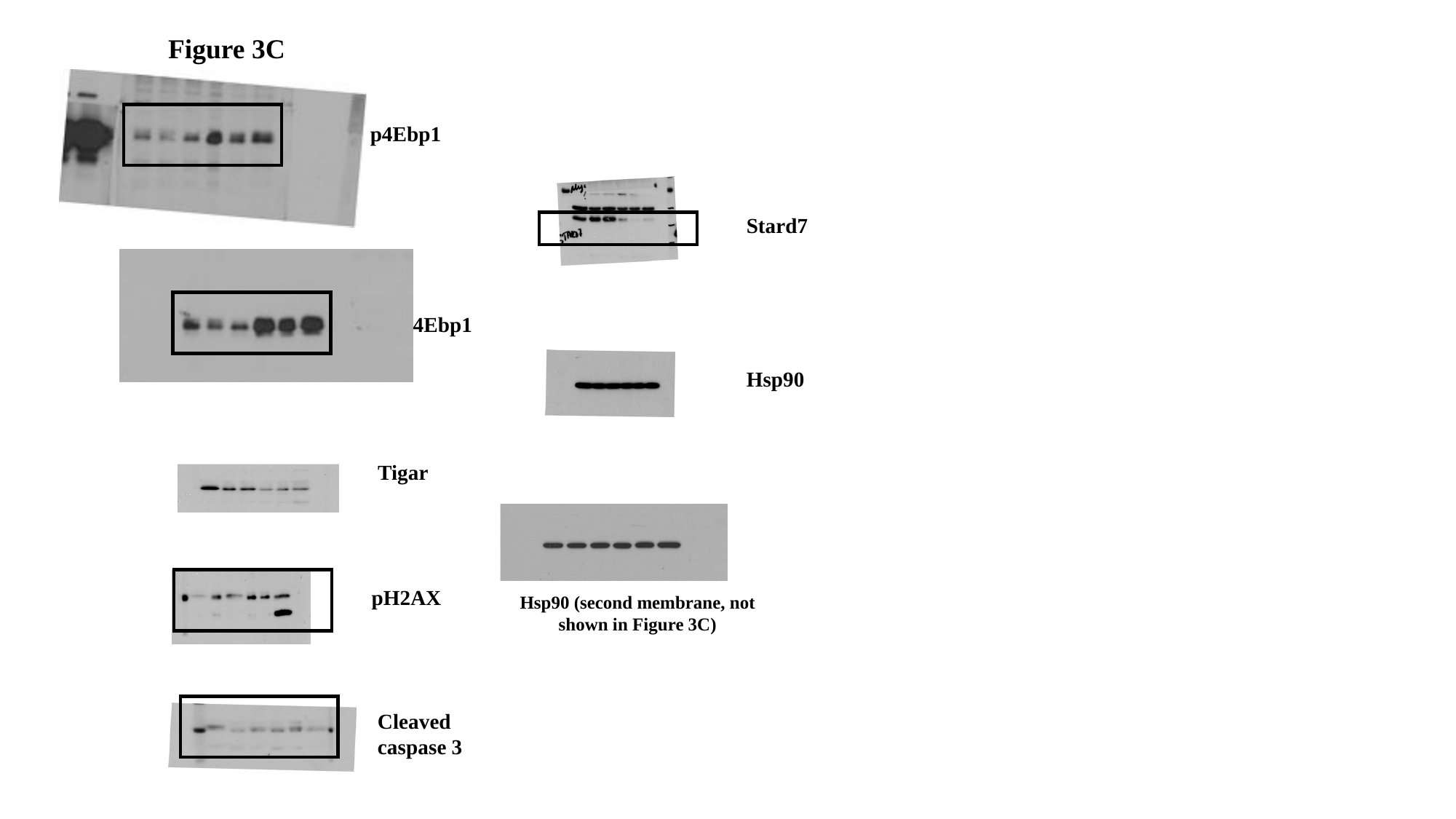

Figure 3C
p4Ebp1
Stard7
4Ebp1
Hsp90
Tigar
pH2AX
Hsp90 (second membrane, not shown in Figure 3C)
Cleaved
caspase 3
