# Supplementary material for: The lipid transfer protein STARD7 controls intestinal tumor development in a context-dependent manner
Source: EMBO Mol Med. 2026 Mar 30;18(5):1771–811. doi: 10.1038/s44321-026-00409-5 (PMC13179355; doi:10.1038/s44321-026-00409-5)
Supplement: Supplementary file 10 — Source data Fig. 5 [file 44321_2026_409_MOESM10_ESM.zip › Fig5/Fig5F/Fig5F.pptx]

## Slide 1
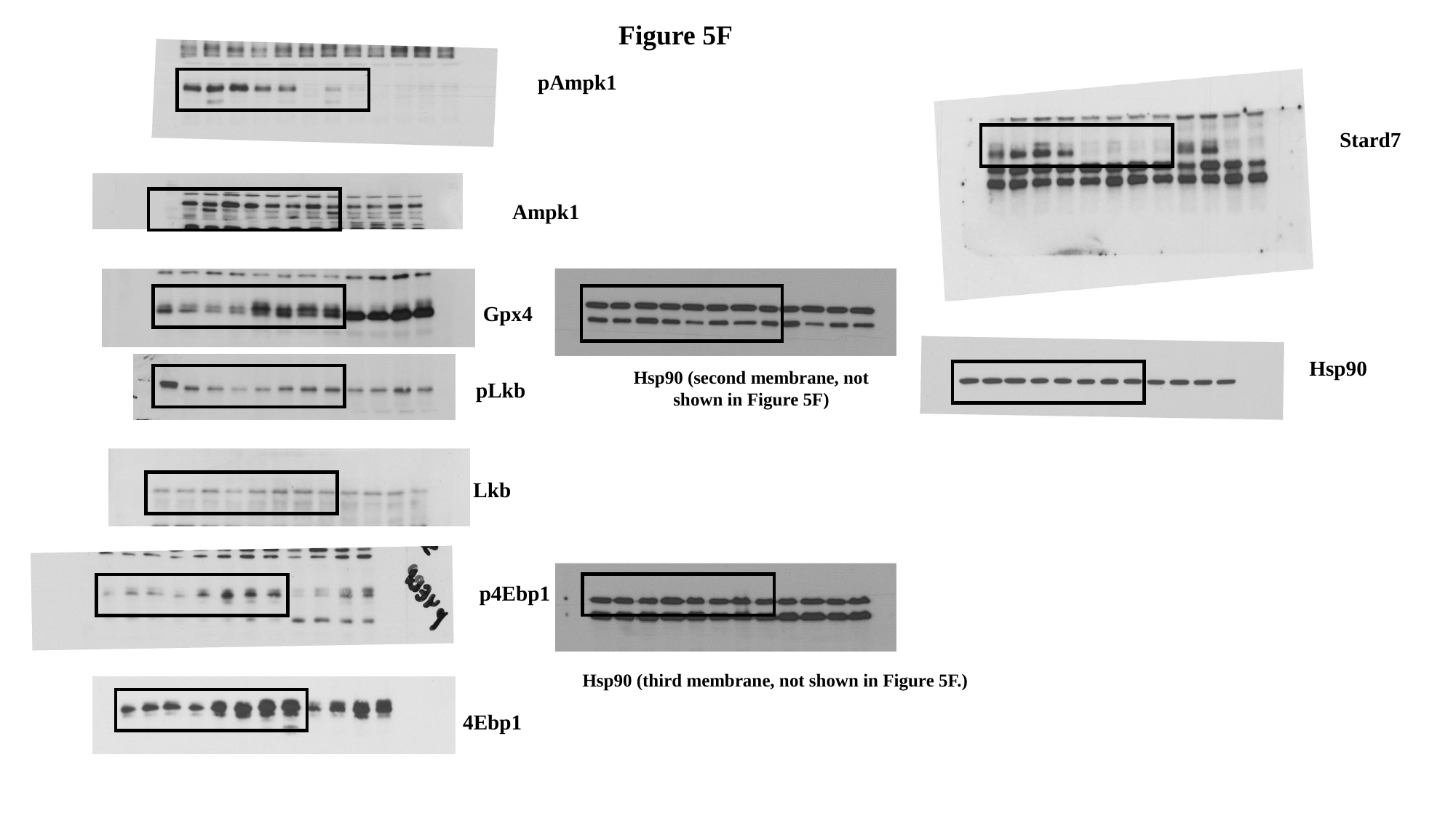

Figure 5F
pAmpk1
Stard7
Ampk1
Gpx4
Hsp90
Hsp90 (second membrane, not shown in Figure 5F)
pLkb
Lkb
p4Ebp1
Hsp90 (third membrane, not shown in Figure 5F.)
4Ebp1
