# Supplementary material for: The lipid transfer protein STARD7 controls intestinal tumor development in a context-dependent manner
Source: EMBO Mol Med. 2026 Mar 30;18(5):1771–811. doi: 10.1038/s44321-026-00409-5 (PMC13179355; doi:10.1038/s44321-026-00409-5)
Supplement: Supplementary file 11 — Source data Fig. 6 [file 44321_2026_409_MOESM11_ESM.zip › Fig6/Fig6E/Fig6E.pptx]

## Slide 1
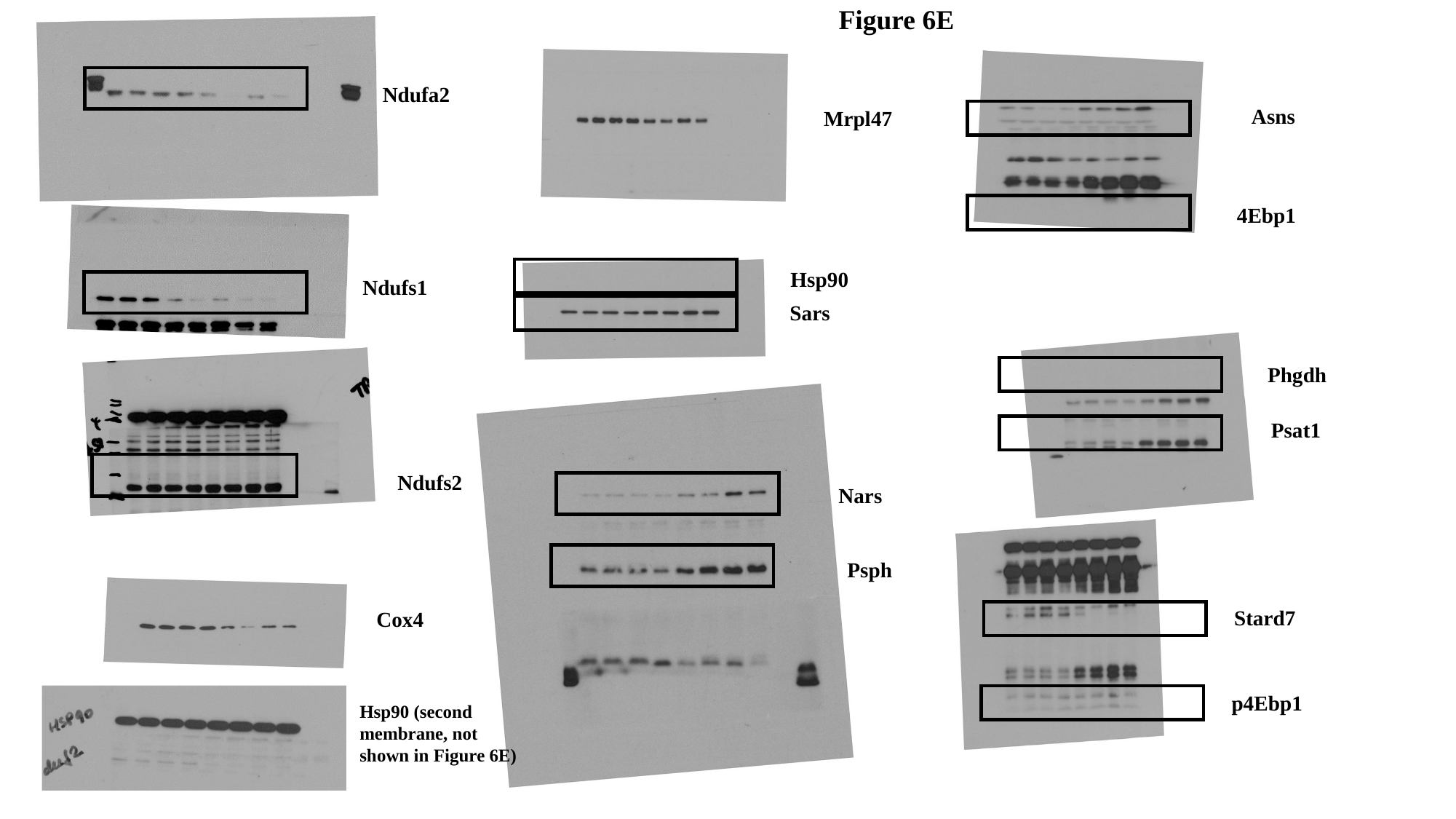

Figure 6E
Ndufa2
Asns
Mrpl47
4Ebp1
Hsp90
Ndufs1
Sars
Phgdh
Psat1
Ndufs2
Nars
Psph
Stard7
Cox4
p4Ebp1
Hsp90 (second membrane, not shown in Figure 6E)
