# Supplementary material for: The lipid transfer protein STARD7 controls intestinal tumor development in a context-dependent manner
Source: EMBO Mol Med. 2026 Mar 30;18(5):1771–811. doi: 10.1038/s44321-026-00409-5 (PMC13179355; doi:10.1038/s44321-026-00409-5)
Supplement: Supplementary file 19 — Figure EV8 Source Data [file 44321_2026_409_MOESM19_ESM.zip › EVF8/EVF8A/EVF8A.pptx]

## Slide 1
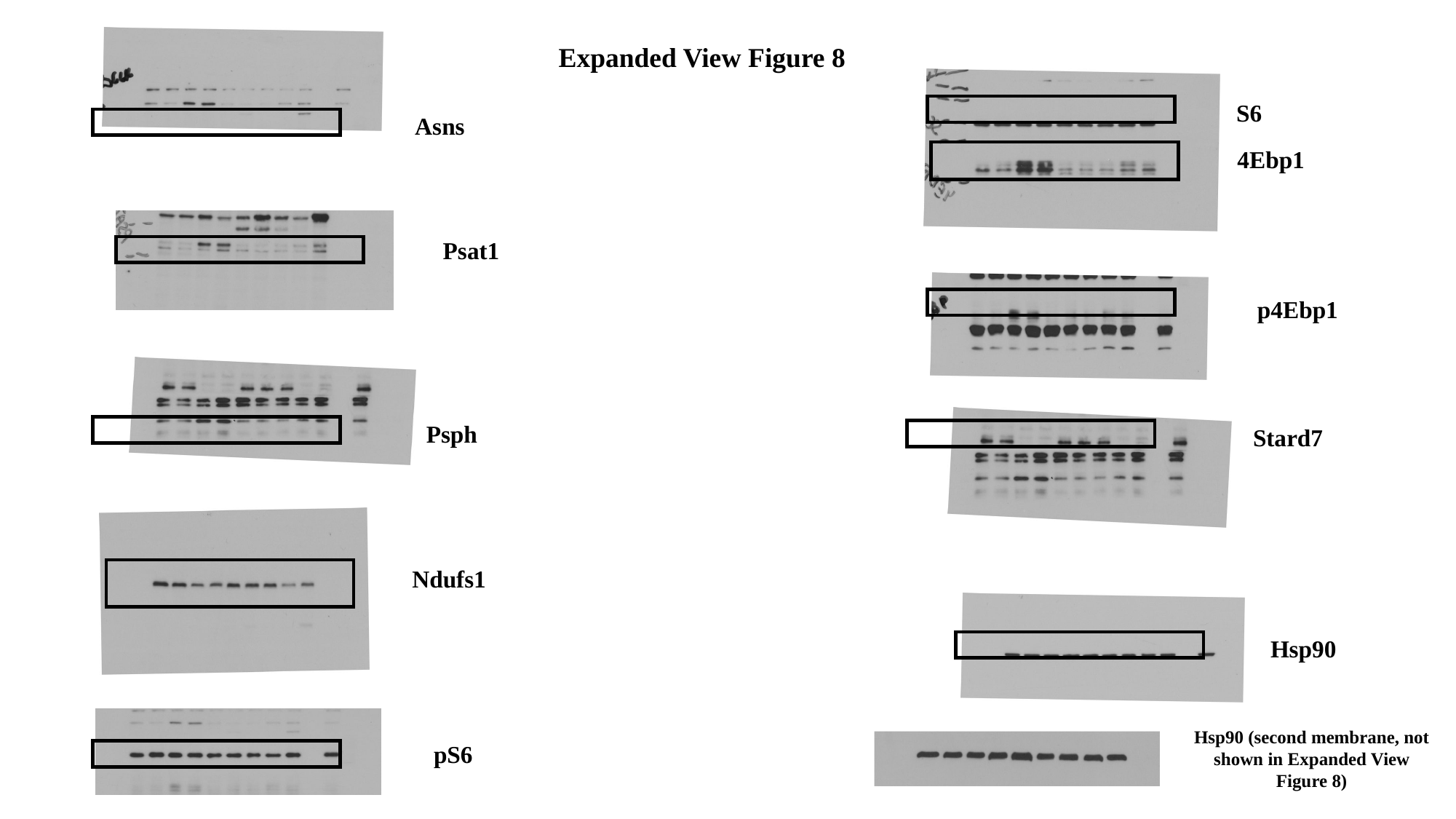

Expanded View Figure 8
S6
Asns
4Ebp1
Psat1
p4Ebp1
Psph
Stard7
Ndufs1
Hsp90
Hsp90 (second membrane, not shown in Expanded View Figure 8)
pS6
