# Supplementary material for: The lipid transfer protein STARD7 controls intestinal tumor development in a context-dependent manner
Source: EMBO Mol Med. 2026 Mar 30;18(5):1771–811. doi: 10.1038/s44321-026-00409-5 (PMC13179355; doi:10.1038/s44321-026-00409-5)
Supplement: Supplementary file 20 — Figure EV9 Source Data [file 44321_2026_409_MOESM20_ESM.zip › EVF9/EVF9.pptx]

## Slide 1
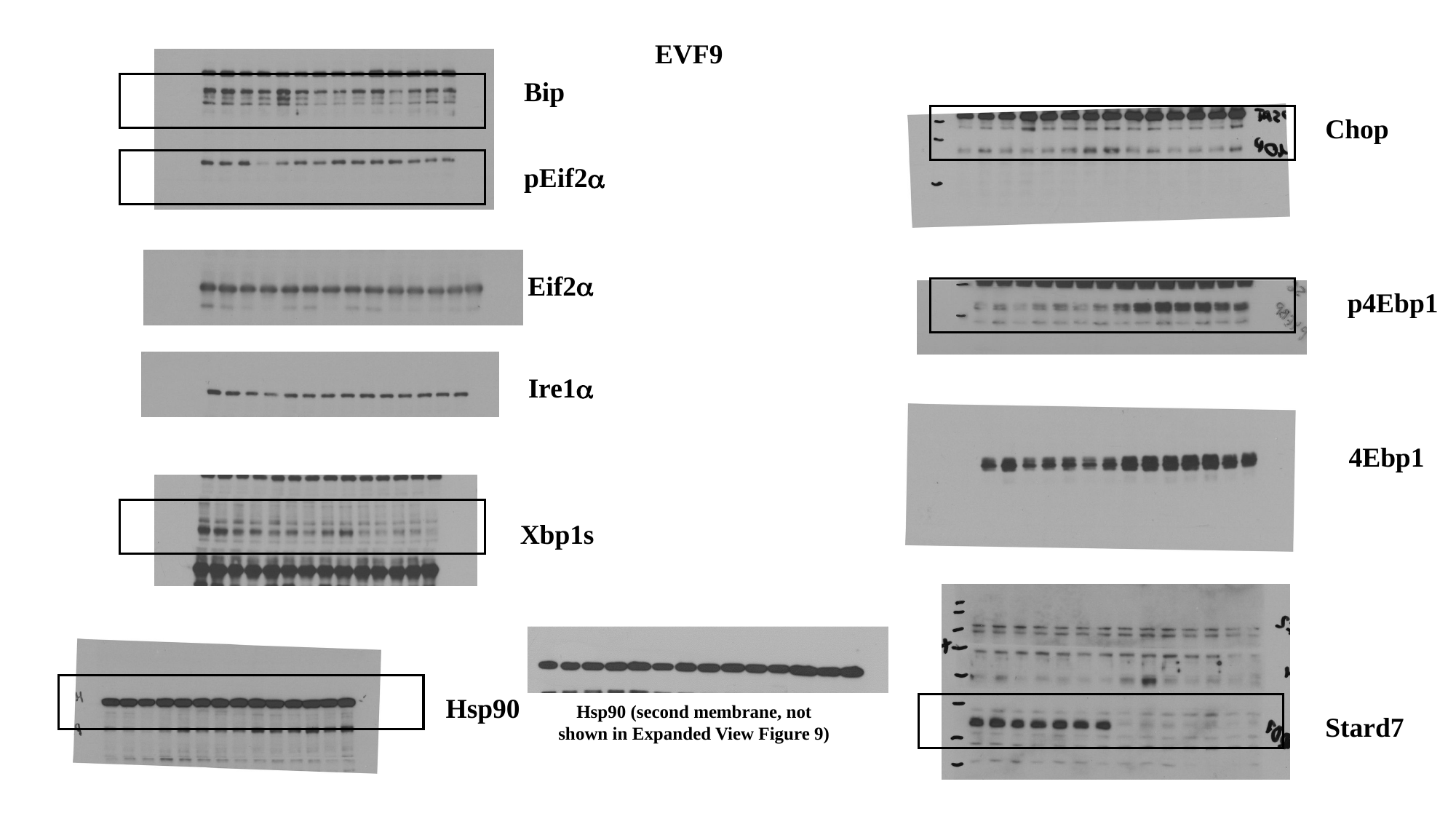

EVF9
Bip
Chop
pEif2a
Eif2a
p4Ebp1
Ire1a
4Ebp1
Xbp1s
Hsp90
Hsp90 (second membrane, not shown in Expanded View Figure 9)
Stard7
