# Supplementary material for: The lipid transfer protein STARD7 controls intestinal tumor development in a context-dependent manner
Source: EMBO Mol Med. 2026 Mar 30;18(5):1771–811. doi: 10.1038/s44321-026-00409-5 (PMC13179355; doi:10.1038/s44321-026-00409-5)
Supplement: Supplementary file 21 — Expanded View Figures [file 44321_2026_409_MOESM21_ESM.pdf]

Expanded View Figures

Figure EV1. Stard7 is dispensable in intestinal homeostasis.

(**A**, **B**) Cell proliferation in the intestinal epithelium is not regulated by Stard7. Anti-Ki67 immunofluorescence or immunochemistry analyses (**A**, **B**, respectively) were conducted in mouse small intestinal or colon sections from 8 to 12-weeks old Stard7$^{Lox/lox}$ ("WT") and Stard7$^{\Delta IEC}$ ("KO") mice (all males). The percentage of Ki67^{+} cells was quantified in both genotypes ($n = 10$ mice for immunofluorescence analyses) (**A**). For immunohistological analyses (**B**), 5 mice per genotype were used and 2–3 fields per slide were randomly selected. 16 and 14 fields, which include 98 and 95 crypts from Stard7$^{\Delta IEC}$ and Stard7$^{Lox/lox}$ mice, respectively, were analysed (means ± S.D.). (**C**, **D**) Stard7 deficiency in the mouse small intestine and in the colon does not influence its architecture nor the number of goblet cells (**C**, **D**, respectively). Immunofluorescence analyses for Mucin 2^{+} cells are illustrated (**C**). Sections from 3 WT and 3 KO mice were analysed. 5 random fields of both WT and KO mice were taken. 37 WT villi and 41 KO villi were analysed (means ± S.D.). Immunohistochemistry analyses (H&E stainings, top panels) showing the colon of the indicated genotypes are illustrated (**D**). Alcian blue stainings (lower panels) to visualize goblet cells in the colon of the indicated genotypes (8 weeks old mice) are illustrated. Alcian blue^{+} cells were counted in 281 crypts/villi from 14 fields and in 340 crypts/villi from 15 fields (5 WT and 5 KO mice, respectively, means ± S.D., Student *T* test, ns = not significant). (**E**) Stard7 deficiency in the mouse small intestinal epithelium does not impact on Paneth cell differentiation, as assessed by immunofluorescence and western blot (WB) analyses for Lysozyme (left and right panels, respectively). Extracts from a total of 5 mice per genotype were analysed and one representative blot is illustrated. The arrow depicts the specific band for Stard7. For anti-Lysozyme immunofluorescences carried out with sections from 3 WT and 3 KO mice, 7 and 8 random fields were taken (WT and KO mice, respectively). 78 WT crypts and 99 KO crypts were analysed (means ± S.D.). (**F**) Markers of epithelial subtypes are properly expressed in the intestinal epithelium of Stard7$^{\Delta IEC}$ mice. Quantitative Real-Time PCR analyses were conducted with extracts from the intestinal epithelium of the indicated mouse genotypes. mRNA levels in one randomly selected Stard7$^{Lox/lox}$ mice were set to 1 and levels in other mice were relative to that after normalization with Gapdh mRNA levels ($n \geq 7$ for both genotypes, means ± S.D., *T* test with Welch correction, Stard7: $^{**}P = 0.0033$; Lgr5: $P = 0.7593$; c-Myc: $P = 0.9974$; Cd44: $P = 0.5355$; Sox9: $P = 0.4429$; Dclk1: $P = 0.1437$; Bmi1: $P = 0.1999$; Olfm4: $P = 0.2518$; Epha2: $P = 0.2895$, ns = not significant. Source data are available online for this figure.

 © The Author(s)

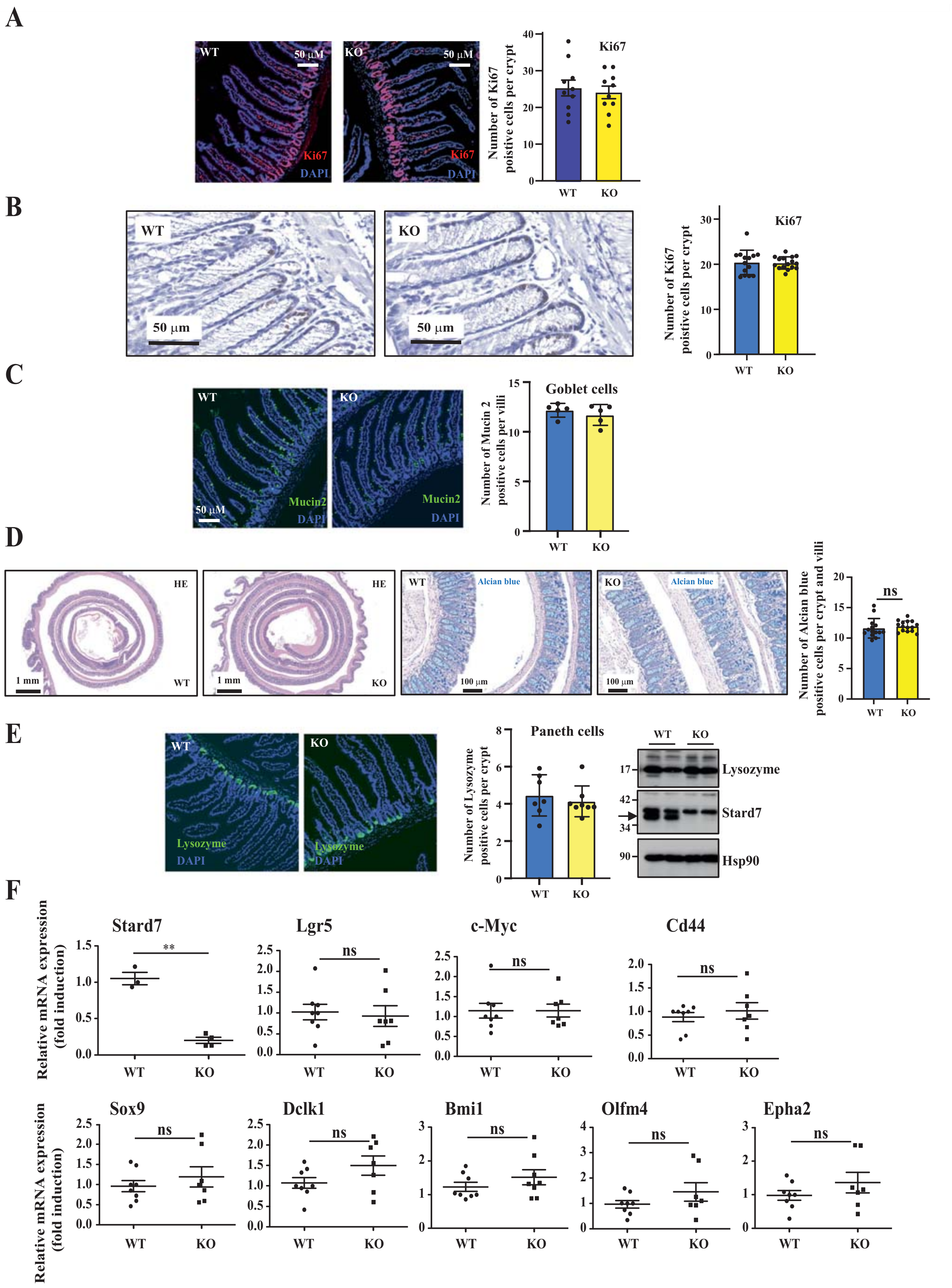

A
WT
50 µM
Ki67
DAPI
KO
50 µM
Ki67
DAPI
Ki67
Number of Ki67 poistive cells per crypt
WT
KO
B
WT
50 µm
KO
50 µm
Ki67
Number of Ki67 poistive cells per crypt
WT
KO
C
WT
50 µM
Mucin2
DAPI
KO
Mucin2
DAPI
Goblet cells
Number of Mucin 2 positive cells per villi
WT
KO
D
HE
1 mm
WT
HE
1 mm
KO
WT
Alcian blue
100 µm
KO
Alcian blue
100 µm
ns
Number of Alcian blue positive cells per crypt and villi
WT
KO
E
WT
Lysozyme
DAPI
KO
Lysozyme
DAPI
Paneth cells
Number of Lysozyme positive cells per crypt
WT
KO
WT
KO
Lysozyme
Stard7
Hsp90
F
Relative mRNA expression (fold induction)
Stard7
**
Lgr5
ns
c-Myc
ns
Cd44
ns
Sox9
ns
Dclk1
ns
Bmi1
ns
Olfm4
ns
Epha2
ns
WT
KO

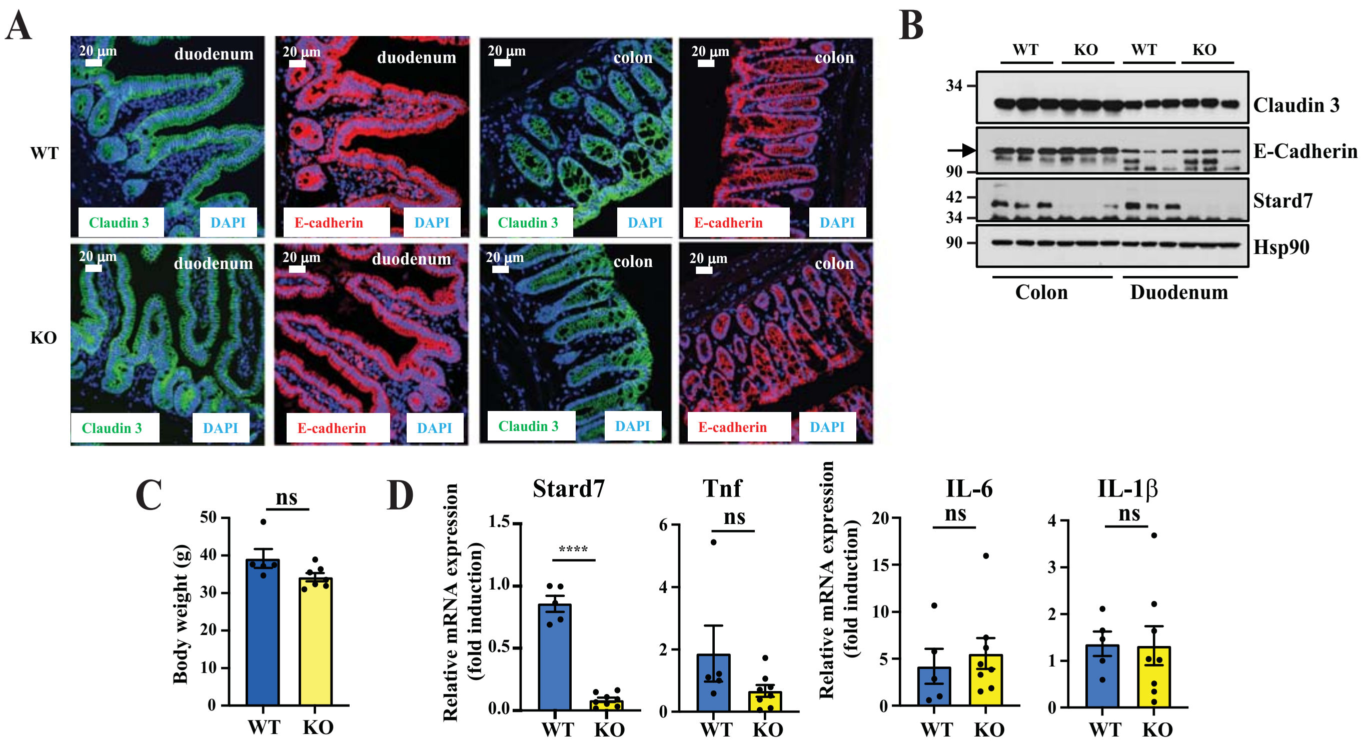

Figure EV2. Stard7 deficiency does not impair the architecture of the intestine and does not lead to spontaneous inflammation.

(**A**, **B**) Stard7 deficiency does not impair the localization and expression levels of tight junctions proteins. Immunofluorescence analyses were conducted to detect the indicated proteins in the intestine of both Stard7$^{Lox/lox}$ and Stard7$^{\Delta IEC}$ mice (**A**). Western blot analyses were also conducted with extracts from the intestinal epithelium (colon and duodenum) of both 8–12 weeks old Stard7$^{Lox/lox}$ and Stard7$^{\Delta IEC}$ mice (3 WT males, 2 KO males and 1 KO female) (**B**). The arrow depicts the specific band for E-Cadherin. (**C**) Stard7 deficiency in the intestine does not change the body weight of aged mice. The body weight of 21-month-old mice of each indicated genotype was quantified ($n = 5$ and 8 for Stard7$^{Lox/lox}$ and Stard7$^{\Delta IEC}$ mice (all males), respectively, means ± S.D., *T* test with Welch correction, NS = no significance). (**D**) Stard7 deficiency in the intestine does not lead to any spontaneous inflammation. Quantitative Real-Time PCR analyses were conducted with extracts from the intestinal epithelium of the indicated mouse genotypes (21-month-old mice, all males). mRNA levels in one randomly selected Stard7$^{Lox/lox}$ mice were set to 1 and levels in other mice were relative to that after normalization with Gapdh mRNA levels ($n = 5$ and 8 for Stard7$^{Lox/lox}$ and Stard7$^{\Delta IEC}$ mice, respectively, means ± S.D., *T* test with Welch correction, Stard7: ****$P < 0.0001$; Tnf: $P = 0.2566$; IL-6: $P = 0.6957$; IL-1β: $P = 0.9350$, ns = not significant). Source data are available online for this figure.

 © The Author(s)

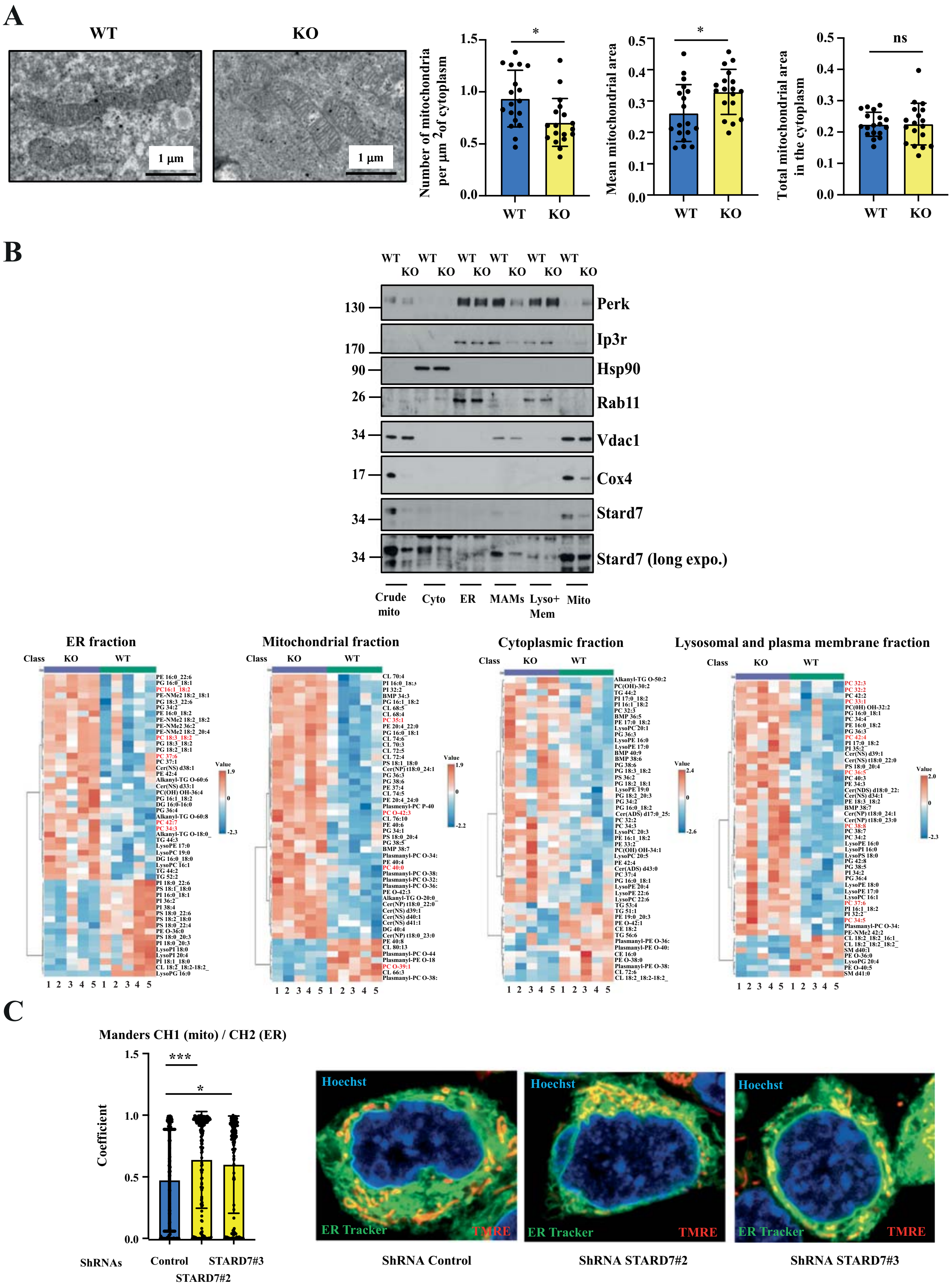

A
WT
KO
1 µm
1 µm
Number of mitochondria per µm 2of cytoplasm
Mean mitochondrial area
Total mitochondrial area in the cytoplasm
*
*
ns
WT KO
B
WT KO WT KO WT KO WT KO WT KO WT KO
130
170
90
26
34
17
34
34
Perk
Ip3r
Hsp90
Rab11
Vdac1
Cox4
Stard7
Stard7 (long expo.)
Crude mito
Cyto
ER
MAMs
Lyso+ Mem
Mito
ER fraction
Mitochondrial fraction
Cytoplasmic fraction
Lysosomal and plasma membrane fraction
Class KO WT
Value
C
Manders CH1 (mito) / CH2 (ER)
Coefficient
***
*
ShRNAs
Control
STARD7#2
STARD7#3
Hoechst
ER Tracker
TMRE
ShRNA Control
ShRNA STARD7#2
ShRNA STARD7#3

Figure EV3. Dramatic lipids redistribution and enhanced mitochondrial-associated membrane contacts (MAMs) in intestinal epithelial cells lacking Stard7.

(**A**) Intestinal epithelial cells lacking Stard7 show enlarged mitochondria. Tissues from 8-week-old Stard7$^{Lox/lox}$ and Stard7$^{\Delta IEC}$ mice were used in transmission electronic microscopy to quantify mitochondrial areas in both experimental conditions (3 WT males; 2 KO males and 1 KO female). On the left, representative images in cells from Stard7$^{Lox/lox}$ and Stard7$^{\Delta IEC}$ mice (WT and KO, respectively). On the right, the number of mitochondria per μm^2 of cytoplasm, the mean mitochondrial area as well as the total mitochondrial area in the cytoplasm were quantified in both experimental conditions (means ± SD, unpaired *T* test with Welch correction. Histogram on the left: *$P = 0.0101$; Histogram on the middle: *$P = 0.0191$; Histogram on the right: $P = 0.9588$, ns = not significant). (**B**) Deregulation of the lipid landscape in organelles upon Stard7 deficiency. ER, mitochondrial, lysosomal and cytoplasmic extracts from the intestine of 8–12 weeks old WT and KO mice ($n = 5$ per genotype) (2 WT males, 3 WT females, 3 KO males, 2 KO females) were subjected to a lipidomic analysis. Phosphatidylcholine derivatives whose levels significantly change in KO versus WT mice are highlighted in red (Student *T* test, $P < 0.05$, 5 mice per genotype). The quality of our organelle-specific protein extracts was assessed through western blot analyses (top panels). Extracts from two independent experiments were analysed and one representative western blot is illustrated. Crude mito = crude mitochondria, Cyto = cytoplasm, ER = endoplasmic reticulum, MAMs = mitochondria-associated membranes, Lyso + Mem = Lysosomal and plasma membrane fractions, Mito = mitochondria. (**C**) Enhanced MAMs in intestinal epithelial cells lacking Stard7. Immunofluorescence analyses were carried out in control versus Stard7-depleted mIECs to quantify the number of MAMs. On the left is illustrated the quantification of the Manders' coefficient M1 (fraction of mitochondria overlapping with the ER) in all experimental conditions. Analyses were carried out with 126, 165 and 123 control, STARD7#2 and STARD7#3 cells, respectively (means ± SD, Dunnett's multiple comparisons test. ShRNAs Control versus STARD7#2: ***$P = 0.0010$; ShRNAs Control versus STARD7#3: *$P = 0.0225$. Higher intensity of yellow corresponds to higher co-localization. Source data are available online for this figure.

 © The Author(s)

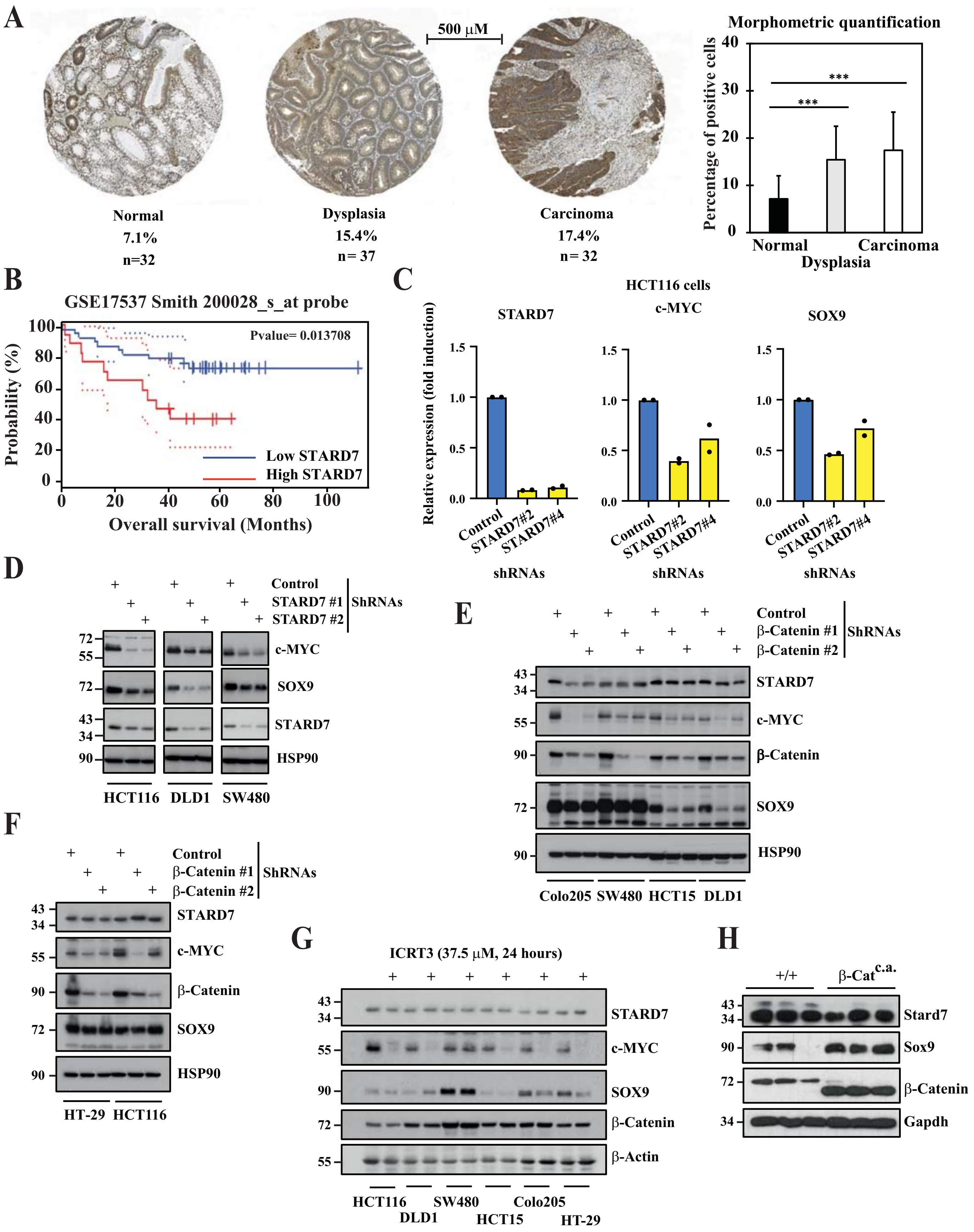
A
500 μM
Normal
7.1%
n=32
Dysplasia
15.4%
n= 37
Carcinoma
17.4%
n= 32
Morphometric quantification
Percentage of positive cells
***
***
Normal
Dysplasia
Carcinoma
B
GSE17537 Smith 200028_s_at probe
Pvalue= 0.013708
Probability (%)
Low STARD7
High STARD7
Overall survival (Months)
C
HCT116 cells
STARD7
c-MYC
SOX9
Relative expression (fold induction)
Control
STARD7#2
STARD7#4
shRNAs
D
Control
STARD7 #1
STARD7 #2
ShRNAs
c-MYC
SOX9
STARD7
HSP90
HCT116
DLD1
SW480
E
Control
β-Catenin #1
β-Catenin #2
ShRNAs
STARD7
c-MYC
β-Catenin
SOX9
HSP90
Colo205
SW480
HCT15
DLD1
F
Control
β-Catenin #1
β-Catenin #2
ShRNAs
STARD7
c-MYC
β-Catenin
SOX9
HSP90
HT-29
HCT116
G
ICRT3 (37.5 μM, 24 hours)
STARD7
c-MYC
SOX9
β-Catenin
β-Actin
HCT116
DLD1
SW480
HCT15
Colo205
HT-29
H
+/+
β-Cat c.a.
Stard7
Sox9
β-Catenin
Gapdh

◀ **Figure EV4. Enhanced expression of STARD7 in colon cancer.**

(**A**) Enhanced STARD7 expression in colon carcinomas. A morphometric quantification was carried out with immunohistochemistry (IHC) data obtained with clinical cases of normal intestinal tissues, dysplasia cases and carcinomas. The percentage of STARD7 positive cells is quantified in these three groups on the right (means + S.D., two-tailed Student *T* tests; Normal versus dysplasia: ****$P < 0.0001$; Normal versus Carcinoma: ****$P < 0.0001$). (**B**) Decreased survival for patients suffering from colon cancer showing high STARD7 mRNA levels (Kaplan–Meier survival plot). Vertical bars denote patients who were censored (i.e. patients still alive at last follow-up or lost to follow-up). (**C**) STARD7 controls levels of candidates induced by Wnt signaling. mRNAs extracted from control or STARD7-depleted HCT116 cells were subjected to quantitative Real Time PCRs to assess levels of STARD7, c-MYC and SOX9. Levels in control cells were set to 1 and levels in other experimental conditions were relative to that after normalization with GAPDH mRNAs. Data from two independent experiments performed in triplicates are shown. (**D**) STARD7 acts upstream of Wnt signalling. Protein extracts from control or STARD7-depleted colon cancer cell lines were subjected to western blot analyses using the indicated antibodies. Extracts from three independent experiments were analysed and one representative western blot is illustrated. (**E**, **F**) β-Catenin does not promote STARD7 expression in colon cancer cell lines. Extracts from control or β-Catenin-depleted cells were subjected to western blot analyses using the indicated antibodies. (**G**) Pharmacological inhibition of Wnt signalling does not downregulate STARD7 expression. Multiple colon cancer cell lines were treated or not with ICRT3 at the indicated concentration for 24 hours and extracts from the resulting cells were subjected to western blot analyses. (**H**) Stard7 expression is not upregulated in the mouse intestinal epithelium from mice showing constitutive Wnt signalling. Extracts from intestinal epithelial cells of control or β-Catenin$^{c.a.}$ mice were subjected to western blot analyses. Source data are available online for this figure.

 © The Author(s)

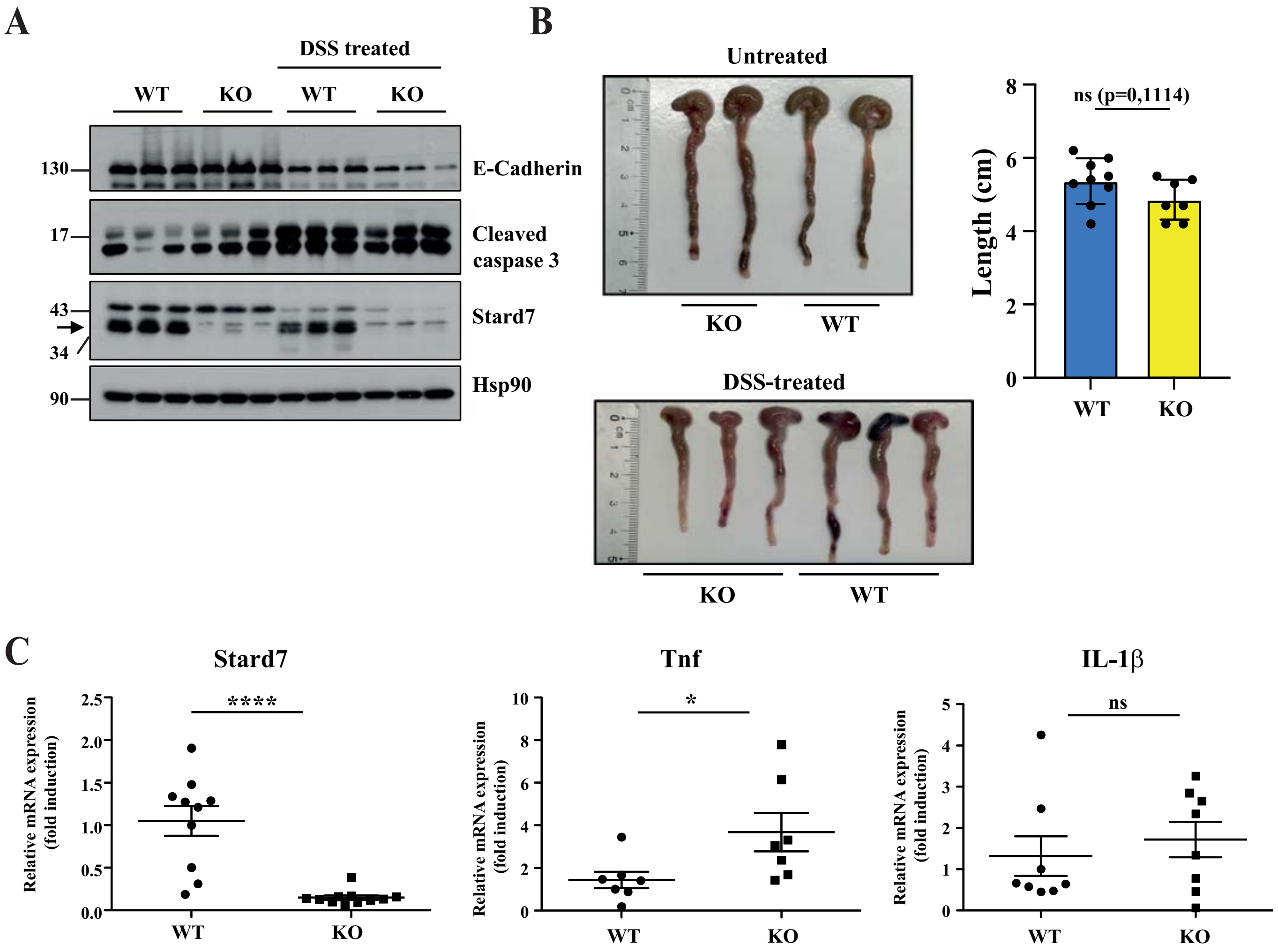

Figure EV5. Stard7 expression in intestinal epithelial cells does not protect from DSS-induced colitis.

(**A**) Stard7 deficiency in IECs does not interfere with cell apoptosis and slightly potentiates the decrease in E-cadherin protein levels upon DSS treatment. In all, 8–12 weeks old mice (all males) were treated or not with a 3% DSS solution in the drinking water for 6 consecutive days. 3 $Stard7^{Lox/lox}$ (WT) and 3 $Stard7^{\Delta IEC}$ (KO) mice were left untreated while 9 $Stard7^{Lox/lox}$ mice and 7 $Stard7^{\Delta IEC}$ mice were subjected to DSS. After sacrifice, IECs from the colon were collected and the resulting extracts were subjected to western blot analyses using the indicated antibodies. The arrow depicts the specific band for Stard7. (**B**) Stard7 deficiency does not potentiate the decrease of the colon length upon DSS administration. Pictures of the colon of the indicated mice genotypes subjected or not to DSS are illustrated. On the right, a quantification of the intestinal length of the indicated mice genotypes subjected to DSS is shown (means ± S.D., *T* test with Welch correction, ns = not significant, $n = 9$ and 7 for WT and KO mice, respectively). (**C**) Stard7 deficiency in IECs potentiates the production of TNF but not IL-1β upon DSS administration. Mice of the indicated genotypes were subjected to DSS as explained in (**A**). The resulting mRNA extracts were subjected to quantitative Real-Time PCR experiments to quantify the indicated candidates. mRNA levels in one randomly selected $Stard7^{Lox/lox}$ (WT) mice were set to 1 and levels in other mice were relative to that after normalization with Gapdh ($n = 9$ and 7 for WT and KO mice, respectively, means ± S.D., Mann–Whitney test. Stard7: ****$P < 0.0001$, Tnf: $P = 0.0262$; IL-1β: $P = 0.5054$, ns = no significance). Source data are available online for this figure.

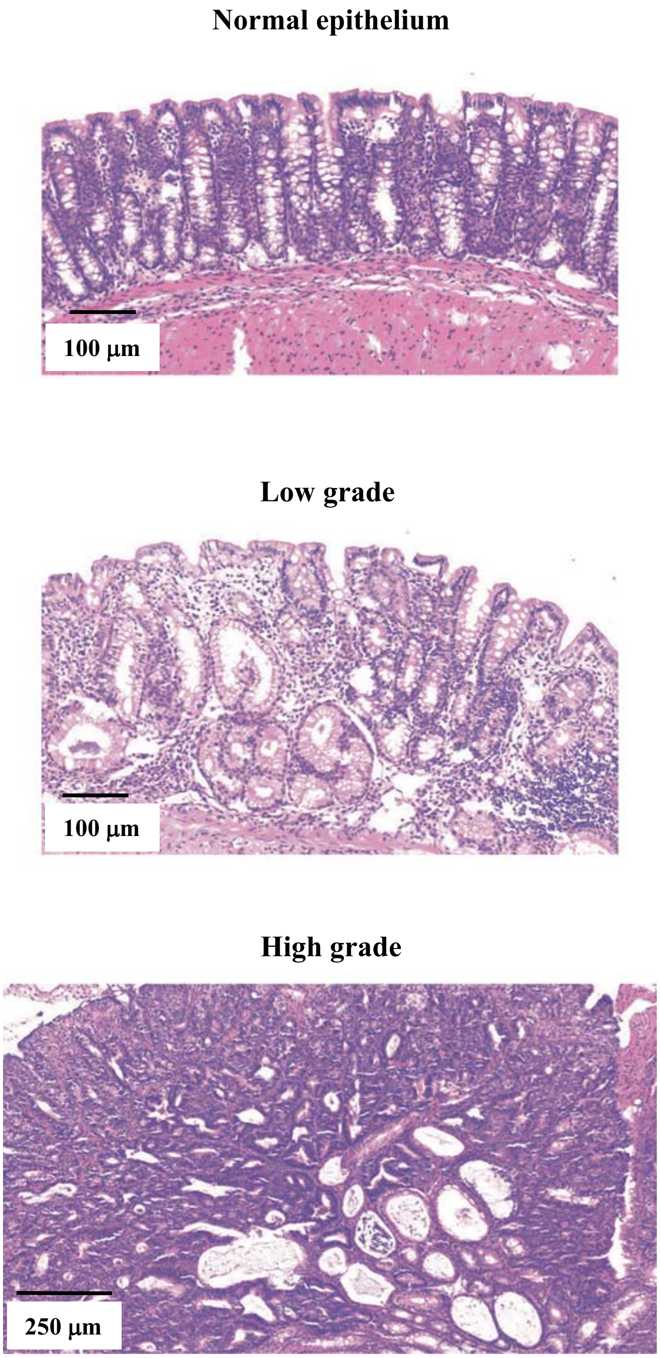

Figure EV6. Stard7 deficiency impairs inflammation-drive tumor development in the intestine.

Representative pictures of colon from AOM/DSS-treated mice are illustrated. These images were used as classification standards for tumor grading (see Fig. 2B).

© The Author(s)

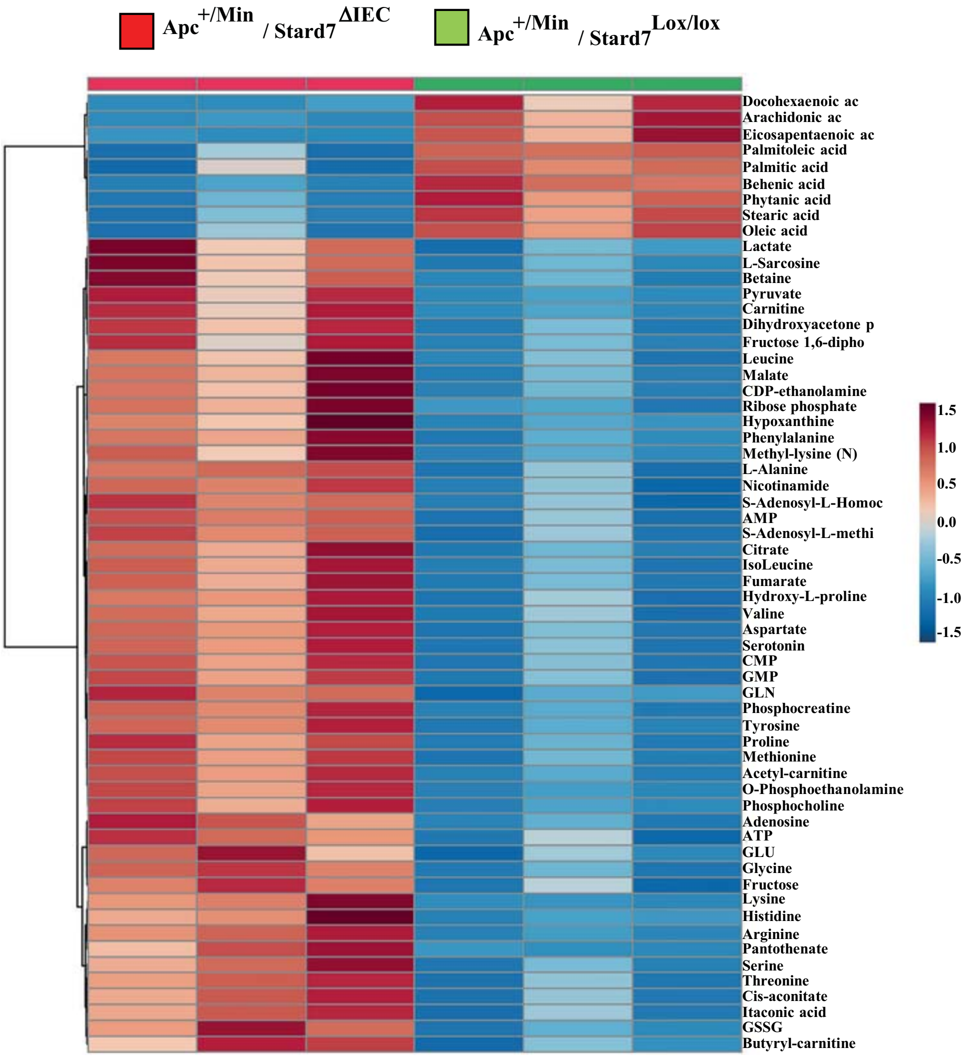

Figure EV7. Metabolic reprogramming upon epithelial Stard7 deficiency in Apc$^{+/Min}$ mice.

The metabolic signature of extracts from the colon of the indicated mouse genotypes (100 days old) was established ($n = 3$ per genotype, all males). This signature reveals decreased levels of fatty acids as well as an accumulation of both TCA intermediates and multiple amino acids in IECs from Apc$^{+/Min}$/Stard7$^{\Delta IEC}$ mice.

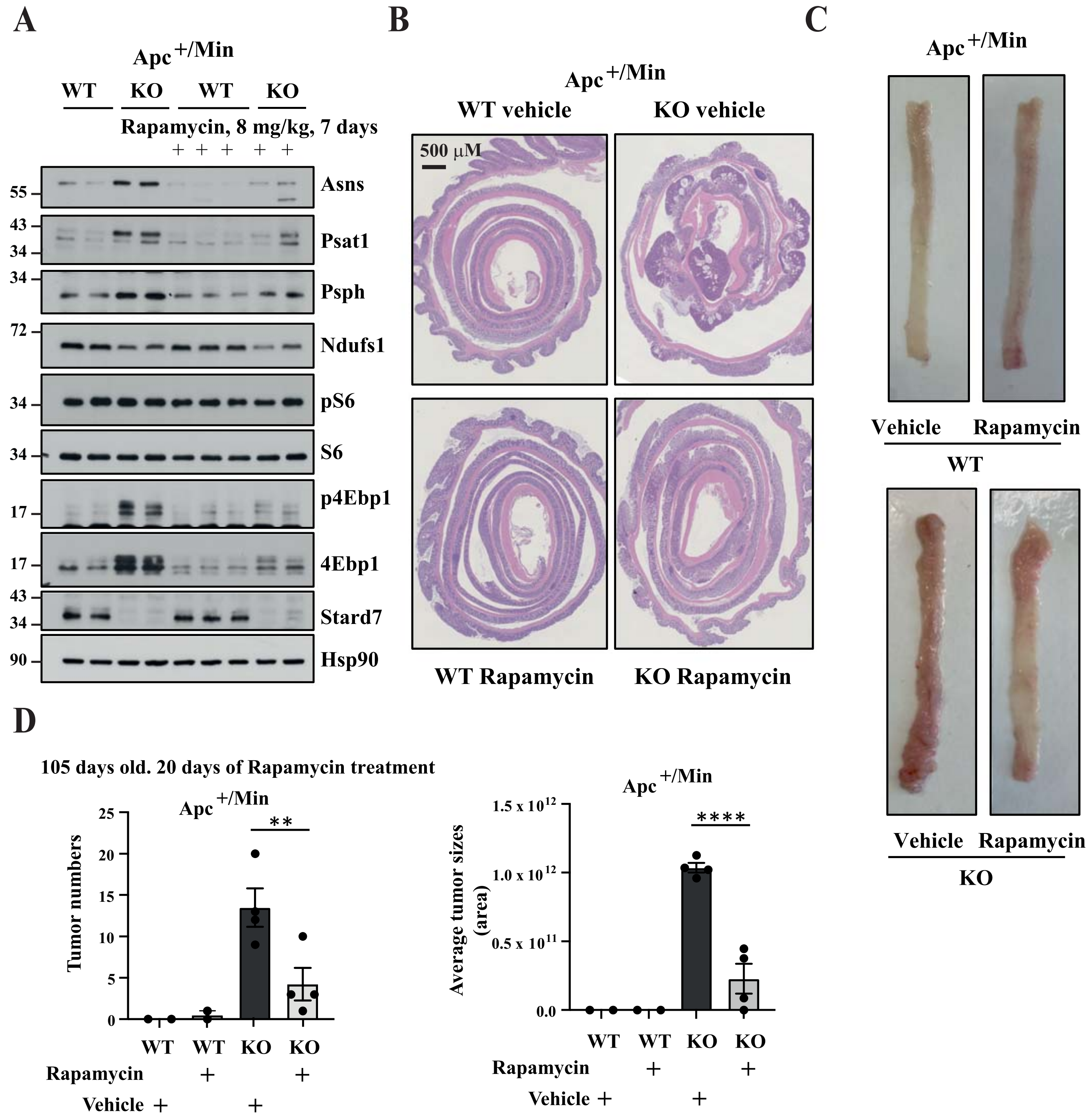

Figure EV8. Wnt-driven intestinal tumors lacking epithelial Stard7 are sensitive to mTORC1 inhibition.

(**A**) Enhanced expression of enzymes involved in serine biosynthesis seen upon Stard7 deficiency in IECs showing or not constitutive Wnt signalling is driven by mTORC1. 105 days old Apc$^{+/Min}$ mice lacking or not Stard7 in IECs (1 WT male, 3 WT females; 4 KO males, 4 KO females) were treated or not with Rapamycin (8 mg/kg) for 7 days and extracts from IECs were subjected to western blot analyses. p4Ebp1 phosphorylation was assessed on Threonine 70. (**B–D**) mTORC1 pharmacological inhibition in Apc$^{+/Min}$/Stard7$^{\Delta IEC}$ mice efficiently triggers tumor regression in the distal colon. Mice of the indicated genotype (1 WT male, 3 WT females; 4 KO males, 4 KO females) were treated or not with Rapamycin for 20 days and consequences on both tumor sizes and numbers were assessed by IHC analyses (**B**). Representative colons of mice of the indicated genotype and treated or not with Rapamycin are illustrated (**C**). The histograms show quantitative data on both tumor numbers and sizes in the indicated experimental conditions (**D**) (means ± S.D., $n = 4$ mice (Rapamycin-treated mice: 4 males; Vehicle-treated mice: 1 male and 3 females), Dunnett's multiple comparisons test. Histogram on the left: Untreated versus Rapamycin-treated KO mice: $^{**}P = 0.0084$; Histogram on the right: Untreated versus Rapamycin-treated KO mice: $^{****}P < 0.0001$). Source data are available online for this figure.

 © The Author(s)

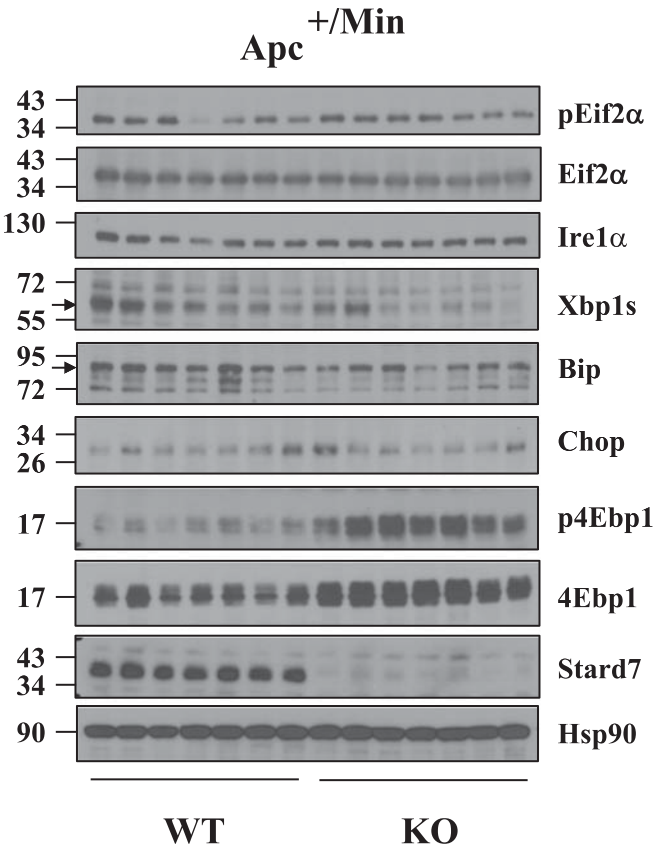

Figure EV9. Epithelial Stard7 deficiency in Apc$^{+/Min}$ mice does not influence protein levels of UPR effectors.

Extracts from 105 days old Apc$^{+/Min}$ mice lacking or not Stard7 in IECs (3 WT males, 4 WT females; 4 KO males, 3 KO females) were subjected to WB analyses using the indicated antibodies. The arrows depict the specific band for Xbp1s and Bip. Source data are available online for this figure.

© The Author(s)
